# SMAD4 and KRAS Status Shape Cancer Cell-Stromal Crosstalk and Therapeutic Response in Pancreatic Cancer

**DOI:** 10.1158/0008-5472.CAN-24-2330

**Published:** 2025-04-15

**Authors:** Eloise G. Lloyd, Muntadher Jihad, Judhell S. Manansala, Wenlong Li, Priscilla S.W. Cheng, Gianluca Mucciolo, Marta Zaccaria, Sara Pinto Teles, Joaquín Araos Henríquez, Sneha Harish, Rebecca Brais, Sally Ashworth, Weike Luo, Paul M. Johnson, Lisa Veghini, Mireia Vallespinos, Vincenzo Corbo, Giulia Biffi

**Affiliations:** 1 University of Cambridge, Cancer Research UK Cambridge Institute, Li Ka Shing Centre, Robinson way, CB2 0RE, Cambridge, UK; 2 Histopathology, Cambridge University Hospitals NHS Foundation Trust, Addenbrooke’s Hospital, Cambridge, UK; 3 Department of Engineering for Innovation Medicine, University of Verona, Verona, Italy; 4 ARC-Net Research Centre, University of Verona, Verona, Italy

## Abstract

Pancreatic ductal adenocarcinoma (PDAC) contains an extensive stroma that modulates response to therapy, contributing to the dismal prognosis associated with this cancer. Evidence suggests that PDAC stromal composition is shaped by mutations within malignant cells, but most previous work has focused on pre-clinical models driven by *Kras^G12D^* and mutant *Trp53*. Elucidation of the contribution of additional known oncogenic drivers, including *Kras*^*G12V*^ mutation and *Smad4* loss, is needed to increase understanding of malignant cell-stroma crosstalk in PDAC. Here, we used single-cell RNA-sequencing to analyze the cellular landscape of *Trp53*-mutant mouse models driven by *Kras^G12D^* or *Kras*^*G12V*^ in which *Smad4* was wild-type or deleted. *Kras^G12D^ Smad4*-deleted PDAC developed a fibro-inflammatory rich stroma with increased malignant JAK/STAT cell signaling and enhanced therapeutic response to JAK/STAT inhibition. SMAD4 loss in *Kras*^*G12V*^ PDAC differently altered the tumor microenvironment compared to *Kras^G12D^* PDAC, and the malignant compartment lacked JAK/STAT signaling dependency. Thus, malignant cell genotype impacts cancer cell and stromal cell phenotypes in PDAC, directly affecting therapeutic efficacy.

## Introduction

Pancreatic ductal adenocarcinoma (PDAC) is the fourth most common cause of cancer-related death and >85% of patients succumb to their disease within 5 years (1). PDAC is characterized by an extensive stroma that contributes to this dismal prognosis. Cancer-associated fibroblasts (CAFs) are abundant stromal cells that modulate PDAC progression and therapy response (2). Distinct populations of CAFs have been described in PDAC and other malignancies (2), including myofibroblastic (myCAFs), inflammatory (iCAFs) and antigen-presenting (apCAFs) CAFs (3–5). Additional subsets have been identified within or across these CAF states due to distinct cells of origin, reprogramming and/or functions (2–4,6–11). Moreover, other stromal cells, including macrophages and neutrophils, have been shown to be heterogenous (12,13). Thus, improving PDAC survival for all patients will likely require a better understanding of the heterogenous nature of the microenvironment across tumors (14). Determining malignant cell-stroma vulnerabilities in distinct patient-relevant contexts may guide the design of precision therapies for PDAC.

PDAC can be classified in basal/squamous and classical/progenitor subtypes, which contain different tumor microenvironments (TMEs) (15–18). However, this classification has not yet translated into improved treatment design, perhaps because these subtypes can co-exist (19–21). Identifying distinct groups of PDAC with specific malignant cell-stroma characteristics and therapeutic sensitivities thus remains a priority. Four main genetic drivers of PDAC have been described: *KRAS* mutations (in >90% patients) - with G12D (>40%) and G12V (>30%) mutations being the most abundant, *TP53* mutations (>70%), *SMAD4* loss (>30%) and *CDKN2A* loss (>30%) (22,23). However, most current knowledge of stroma composition comes from KRAS^G12D^, p53 mutant KPC (*Kras*^*LSL-G12D/+*^; *Trp53*^*LSL-R172H/+*^; *Pdx1*-*Cre*) genetically engineered mouse models (GEMMs) (24), which recapitulate features of patient disease progression but may not capture stromal differences across human PDAC. Indeed, evidence indicates that mutations in PDAC malignant cells can differently shape the stroma (25–28). For example, increased fibrosis and reduced CD8^+^ T cell infiltration has been associated with *TP53*-mutated PDAC, while *BRCA*-mutated and *BRCA*-WT PDACs have different CAF composition (25,26). Moreover, the stroma affects PDAC progression and therapy response (2). Therefore, distinct PDAC groups may respond differently to therapies depending on their genotype and associated TME.

Malignant cell transforming growth factor beta (TGF-β) signaling is a key player in shaping the PDAC stroma since it drives myCAF formation (5,11). Loss of *SMAD4*, which is involved in TGF-β signaling, is typically a late event in PDAC development and correlates with metastasis formation and worse prognosis (23,29). This effect may be in part dependent on the ability of TGF-β signaling-deficient malignant cells to remodel epithelial and stromal compartments (28). To determine how SMAD4 deficiency tunes the PDAC TME and identify candidate dependencies of these aggressive tumors, we established *Trp53*-mutant mouse models driven by *Kras^G12D^* or *Kras*^*G12V*^ in which *Smad4* was wild-type (WT) or deleted.

## Materials and Methods

### Human PDAC tissues

Human PDAC tissues used in this study were obtained from surgical resections of patients (both males and females) treated at the University and Hospital Trust of Verona (Azienda Ospedaliera Universitaria Integrata, AOUI) for curative intent. Written informed consent was acquired from patients before specimens’ acquisition. Specimens were acquired under protocols approved by the AOUI Ethics Committee (Comitato Etico Azienda Ospedaliera Universitaria Integrata): approval number 1911 (Prot. n 61413, Prog 1911 on 19/09/2018) and approval number 3456 (Prot. n. 55859, Prog 3456 on 22/09/2021). Clinical information of the human PDAC tissues used, including sex, age, histological diagnosis, staging and genotype information for *KRAS, TP53, CDKN2A, SMAD4* and *TGFBR2*, is provided in Supplementary Table S1. Genotype information for PDA-XX samples has been previously published and is based on targeted sequencing approach of organoids derived from those tissues (30). The genotype for all VR-Px samples was assessed in the same way. All tissues analyzed in this study were treatment naïve.

### Mouse models

Male and female C57BL/6J (strain number 632, Charles River, RRID:IMSR_JAX:000664), athymic nude nu/nu (strain number 490, Charles River, RRID:IMSR_CRL:490) and NOD SCID gamma (NSG) mice (strain number 614, Charles River, RRID:IMSR_JAX:005557) were purchased from the Charles River Laboratory (7-9-week-old at arrival). All animals are housed in accordance with the UK Home Office “Code of Practice for the Housing and Care of Experimental Animals” guidelines. All animal procedures and studies were reviewed by the Cancer Research UK Cambridge Institute (CRUK-CI) Animal Welfare Ethics Review Board (AWERB), approved by the Home Office and conducted under project license number PP4778090 in accordance with relevant institutional and national guidelines and regulations.

### Orthotopic transplantation models of PDAC

Pancreas injections were conducted as previously described (5,11). Briefly, single cells (10,000 murine cells/mouse or 30,000-700,000 human cells/mouse (700,000 cells were injected for NSG cohorts 5 and 6) prepared from PDAC organoid cultures were resuspended as a 35 μL suspension of 50% Matrigel in PBS and injected into the pancreas of 8-10-week-old mice. Pancreatic tumors were imaged using the Vevo 2100 Ultrasound at two different orientations with respect to the transducer. Tumor volumes were measured at two or three angles, whenever possible, using the Vevo LAB software program (version 5.7.0). Tumor volume analyses were performed blindly prior to plotting the data for visualization. Only mice with successful (i.e., non-leaked) orthotopic injections were included for tumor volume and metastases analyses. For each experiment, SMAD4 WT and SMAD4-deficient/knockout (KO) cohorts were transplanted on the same day and later imaged by ultrasound on the same day. NSG mice were used for transplantation of human PDAC organoids, nu/nu mice were used for transplantation of KvPC (i.e. from the *Kras*^*FRT-LSL*-G12V*-FRT/+*^; *Trp53*^*LSL*-R172H^; *Pdx1-Cre*; *Rosa26-FlpO*^*ERT2*^ GEMM) organoids since this GEMM had a mixed, not pure C57BL/6J, background (31). Nu/nu mice were also used for transplantation of KPC PDAC organoids that were generated by stable expression of CAS9, rather than CAS9 electroporation (see below for details about the two CRISPR technologies used to generate *Smad4* WT and *Smad4* KO KPC organoid lines), and to enable comparison with KvPC PDAC models. C57BL/6J mice were used for all other transplant models. Tumors were collected at similar sizes and weights for downstream analyses.

### *In vivo* AZD1480 treatment study

The drug was prepared daily as a suspension in 0.1% Tween80, 0.5% hydroxyl propyl methyl cellulose in sterile water, and sonicated before administration. Palpable pancreatic tumors in C57BL/6J mice were imaged prior to enrolment (day -1) and at endpoint (day 14) using the Vevo 2100 Ultrasound at two-three different orientations, whenever possible, with respect to the transducer. Mice with tumor diameters of 5 to 8 mm were randomized and enrolled 1 day after scanning (i.e. day 0). Mice were administered vehicle or 50-60 mg/kg of AZD1480 (S2162, CAS 935666-88-9; Selleck or HY-10193, CAS 935666-88-9; MedChem Express) for 14 days, once a day (in the AM) via oral gavage. Tumor volumes were measured at two or three angles, whenever possible, using the Vevo LAB software program (version 5.7.0), and tumor growth based on these measurements was calculated by dividing the volume at day 14 for the volume at day -1. Tumor volume analyses were performed blindly prior to plotting the data for visualization.

### Cell lines and cell culture

Murine pancreatic stellate cells (PSCs) (SV40-immortalized, C57BL/6J background), apart from PSC21, and murine PDAC KPC organoid lines (C57BL/6J background) were previously described (4,32). The PSC21 line was generated, immortalized and characterized as previously described (4,11). Briefly, to establish PSC21, we utilized two and a half pancreata from male C57BL/6J mice, and a density gradient centrifugation method with Histodenz (D2158; Sigma-Aldrich) and Gey’s Balanced Salt Solution (G9779; Sigma-Aldrich). Murine KvPC (i.e. FPC organoids from the *Kras*^*FRT-LSL*-G12V*-FRT/+*^; *Trp53*^*LSL*-R172H^; *Pdx1-Cre*; *Rosa26-FlpO*^*ERT2*^ GEMM, not pure C57BL/6J background) organoids were kindly obtained via Material Transfer Agreement (MTA) from Professor Tuveson (Cold Spring Harbor Laboratory, CSHL) and have been previously published (31). They were then cultured for three passages in complete organoid media with 10 μM Nutlin-3a (SML0580; Sigma-Aldrich) to enrich for organoids that undergone p53 loss of heterozygosity (LOH) and generate T-LOH organoid lines, as previously done for KPC organoids (32). By inhibiting the interaction between p53 and the E3 ubiquitin protein ligase MDM2, Nutlin-3a leads to WT p53 activation, consequential depletion of cells that retain the WT *Trp53* allele and upregulation of mutant p53 levels (32–34). Mouse PSCs were cultured in DMEM (41966029; Gibco) containing 5% FBS. All cells were typically cultured up to 40 passages, whenever possible, at 37°C with 5% CO2. Cell line authentication was previously performed at the CRUK-CI for murine PSC4 and PSC5. Mycoplasma testing for murine PSCs was performed prior to each freezing. Human organoids were kindly obtained via MTA from Professor Tuveson (CSHL) and have been previously published (35). All tissue donations to generate these organoids had been reviewed and approved by the Institutional Review Board of CSHL and all clinical institutions. Written informed consent was obtained prior to acquisition of tissue from all patients. The studies were conducted in accordance with recognized ethical guidelines (Declaration of Helsinki). Clinical information of the human PDAC organoids used is provided in Supplementary Table S2.

### *In vitro* cell treatments

PSCs were treated in Matrigel in 5% FBS DMEM with PDAC organoid conditioned media (CM) for as long as specified in the figure legends. KPC and KvPC PDAC organoids were treated with 8 μM AZD1480 for 48 hours prior to Western blot analysis of phospho-STAT3 (p-STAT3) and STAT3 levels or for 144 hours for proliferation assays. PDAC organoids were treated with 5-20 ng/mL human TGF-β1 (T7039-2UG; Sigma), 2 μM A83-01 (SML0788; Sigma), 10 ng/mL interleukin 1 alpha (IL-1α, 400-ML-005/CF; R&D Systems), 3-5 μg/mL IL-1α-neutralizing antibody (MAB4001; R&D Systems; RRID:AB_2124216) or IgG control (400902; BioLegend) in reduced media (i.e. 5% FBS DMEM) for 48-72 hours prior to collecting protein or RNA, as indicated in the figure legends. Organoids were treated with 1-2 nM Trametinib (HY-10999, CAS 871700-17-3; MedChemExpress) and 1 μM Pexmetinib (HY-16782, CAS 945614-12-0; MedChemExpress) in 5% FBS DMEM media for 72 hours prior to collecting protein or RNA.

### PCR-based genotyping of *Trp53* 1loxP

PCR-based genotyping of *Trp53* 1loxP was previously described (32). Briefly, organoids were harvested and centrifuged at 1,000 rpm for 5 min at 4°C. Genomic DNA was extracted from organoids with DNEasy Blood & Tissue Kit (69504; Qiagen). *Trp53* 1loxP genotyping PCR reaction was performed in a 20 μL reaction using AmpliTaq Gold 360 master mix (4398881; Thermo Fisher Scientific), 0.5 μM each primer (p53loxF AGCCTGCCTAGCTTCCTCAGG and p53loxR CTTGGAGACATAGCCACACTG) and 100 ng of template DNA. The PCR cycling conditions were 95°C for 10 min, followed by 40 cycles at 95°C for 30 s, 56°C for 30 s, and 72°C for 30 s, then 72°C for 7 min (BioRad T100 Thermocycler). PCR products were separated on a 2% agarose gel in TAE buffer. Gel imaging was performed with a Syngene U:Genius 3.

### *Smad4* CRISPR/Cas9 knockout

#### We utilized two different strategies to generate *Smad4* KO organoids

To knock out *Smad4* in organoids for KPC T6-LOH and T69A KO 1 and KO 2 clones/pools, lenti-Cas9-Blast plasmids (52962; Addgene) for stable expression of CAS9 protein were used as previously described (5). Briefly, organoids were prepared as single cells and infected and selected using 2 μg/mL blasticidin (A11139-03; Thermo Fisher Scientific). Single guide RNAs (sgRNAs) targeting exon 2 (for KO 1) or exon 3 (for KO 2) were designed using Benchling (RRID:SCR_013955) and cloned into the LRGN (LentisgRNA-EFS-GFP-neo) plasmid. Thus, organoids expressing these guides are green fluorescent protein (GFP)-positive and were used for the establishment of organoid-derived mouse models used for single-cell RNA-sequencing (scRNA-seq). Organoids were plated as single clones in the presence of geneticin (10131035; Thermo Fisher Scientific). Knockout was confirmed by western blot analysis (4-5 clones/guide/organoid line). sgRNAs against the *Rosa26* locus were included to generate control (i.e. WT) lines, and these *Smad4* WT controls were kept as pools.

To knock out *Smad4* in organoids for KPC T6-LOH and T69A 263 and 264 clones/pools (i.e. KO 3 and KO 4) and KvPC T93-LOH and T95-LOH 263 and 264 clones/pools (i.e. KO 3 and KO 4), CRISPR guides (phosphorothionate-modified sgRNA, 263: ATCAGGCCACCTCCACAGAC, 264: AGACGGGCATAGATCACATG) were designed against exon 3 of the murine *Smad4* gene (ENSMUST00000025393.14). A guide which targets the *Rosa26* locus was also included to generate control (i.e. WT) lines (265: GAAGATGGGCGGGAGTCTTC), and these *Smad4* WT controls were kept as pools. Mouse PDAC organoids were dissociated into single cells and 100,000 cells were electroporated using an Amaxa 4D Nucleofector unit (Lonza) with 4 μg TrueCut spCas9 protein V2 (A36498; Invitrogen) and 80 pmol guide RNA (Synthego), using program CM-137 and P3 nucleofector solution (V4XP-3032; Lonza). A cell pellet was taken 3- and 10-days post electroporation, and genomic DNA was extracted using the DNeasy blood and tissue kit (69506; Qiagen). Exon 3 of *Smad4* was amplified by PCR using the Q5 High Fidelity DNA polymerase (M0491S; NEB). *Smad4* primers used were FWD: TTCCCTTCAGCAGAAGCTGG, and REV: TGCTTCCCATACTGTTTGCA. Amplicons were subjected to Sanger sequencing and analyzed using Synthego ICE web tool to calculate the percent editing in a pool. Organoids were plated as single clones and knockout was confirmed by western blot analysis (6-7 clones/guide/ KPC organoid line; 5-7 clones/guide/ KPC organoid line).

### Western blot analyses

PSCs and organoids were harvested in Cell Recovery Solution (354253; Corning) supplemented with complete, mini protease inhibitors (11836170001; Roche) and a phosphatase inhibitor cocktail (4906837001; Roche) and incubated for 30 min at 4°C. Cells were pelleted at 1500 rcf for 5 min and lysed in 0.1% Triton X-100, 15 mmol/L NaCl, 0.5 mmol/L EDTA, 5 mmol/L Tris, pH 7.5, supplemented with complete, mini protease inhibitors (11836170001; Roche) and a phosphatase inhibitor cocktail (4906837001; Roche). Cells were incubated on ice for 30 min briefly vortexed and pelleted at 13,200 rpm for 10 min at 4°C. Concentration of protein collected in the supernatant was determined using DC protein assay (5000113-5; Bio-Rad). Standard procedures were used for western blotting. Primary antibodies used were ACTIN (8456; Cell Signaling Technology; RRID:AB_10998774), HSP90 (07-2174;Millipore; RRID:AB_10807022), SMAD4 (sc-7966; Santa Cruz; RRID:AB_627905), p-STAT3 (9145; Cell Signaling Technology; RRID:AB_2491009), STAT3 (9139; Cell Signaling Technology; RRID:AB_331757), p53 (P53-CM5P-L; Leica; RRID:AB_2744683), HIF-1α (14179; Cell Signaling Technology; RRID:AB_2622225), p-p44/42 (p-Erk1/2) (4370; Cell Signaling Technology; RRID:AB_2315112), p44/42 (Erk1/2) (4695; Cell Signaling Technology; RRID:AB_390779), p-p38 (4511; Cell Signaling Technology; RRID:AB_2139682), p38 (9212; Cell Signaling Technology; RRID:AB_330713), HSP60 (12165; Cell Signaling Technology; RRID:AB_2636980), SMAD2 (5339; Cell Signaling Technology; RRID:AB_10626777), p-SMAD2/SMAD3 (8828; Cell Signaling Technology; RRID:AB_2631089) and GFP (ab6673; Abcam; RRID:AB_305643). Proteins were detected using appropriate HRP-conjugated secondary antibodies (Jackson ImmunoResearch Laboratories). All western blots are representative examples and have been repeated for at least two biological replicates.

### Proliferation assays

For proliferation assays of PDAC organoids, 5,000 single cells were plated in 50 μL of 100% Matrigel on 24-well plates (Corning/Nunc) and cultured in 500 μL of reduced media (i.e. 5% FBS DMEM) or complete mouse organoid media (36). Organoid proliferation was followed for 96-144 hours with an Incucyte organoid module (Sartorius) with measurement of the organoid area per well every 3 hours (with 4 technical replicates per measurement). Data were normalized to the first measurement (at 3 hours post-plating on day 0). For proliferation assays of PDAC organoids with 8 μM of the JAK inhibitor (JAKi) AZD1480, 2,000-3,000 single cell organoids were plated in 25 μL of 100% Matrigel on 48-well plates (Corning/Nunc) and cultured in 250 μL of reduced media. Organoid proliferation was followed as before, data were normalized to the first measurement (i.e. average of 6 technical replicates, at 3 hours post-plating on day 0) and used to calculate cell viability in comparison to the respective DMSO-treated controls.

### Immunohistochemical and histological analyses

Human and murine organoid-derived *Smad4* WT and *Smad4* KO tumors were collected at comparable tumor sizes and weights. Standard procedures were used for immunohistochemistry (IHC). Primary antibodies for IHC were alpha smooth muscle actin (αSMA, ab5694; Abcam; RRID:AB_2223021), SMAD4 (sc-7966; Santa Cruz; RRID:AB_627905), E-cadherin (ECAD, 610182; BD Biosciences; RRID:AB_397581), Ki67 (12202; Cell Signaling Technology; RRID:AB_2620142), pH3 (9701S; Cell Signaling Technology; RRID:AB_331535) and p-STAT3 (9145; Cell Signaling Technology; RRID:AB_2491009). Hematoxylin (H-3404-100, Vector Lab) was used as nuclear counterstain. Masson’s trichrome and Hematoxylin & Eosin stains were performed according to standard protocols by the Histology core at the CRUK-CI. FOXP3 and Ly6G stains were also performed by the Histology core. Briefly, after baking at 60°C for 1 hour, sections were dewaxed and rehydrated on Leica’s automated ST5020. The staining was performed on Leica’s automated Bond-III platform in conjunction with their Polymer Refine Detection System (DS9800) and a modified version of their standard template, with protein block (X090930-2; Dako), anti-rat secondary antibody (A110-322A; Bethyl Laboratories; RRID:AB_10681533) and DAB Enhancer (AR9432; Leica). Primary antibodies used were Ly6G (127601; BioLegend; RRID:AB_1089179) and FOXP3 (14-5773; eBioscience; RRID:AB_467576). Antigen retrieval was performed at 100 °C for 20 min in citrate or Tris EDTA for Ly6G or FOXP3, respectively. De-hydration and clearing were performed on Leica’s automated ST5020 before sections were mounted on Leica’s coverslipper, CV5030. Stained sections were scanned with Aperio ScanScope CS and analyzed using ImageScope software (RRID:SCR_014311) Positive Pixel Count algorithms or a Nuclear v9 algorithm, depending on the marker quantified.

Images of tissue slides were obtained with an Axio Vert.A1 (ZEISS) apart for human PDAC tumors, which were snapshotted from the ImageScope software. The percentage of collagen area was determined by calculating the percentage of blue pixels relative to the entire stained area. To quantify αSMA stain, the percentage of positive pixels was calculated relative to the entire section. To quantify p-STAT3, pH3 and Ki67 stains, the percentage of positive nuclei was calculated relative to the total number of nuclei. For Masson’s trichrome and αSMA quantification of human PDAC tissues, only the PDAC area was included for analysis, following annotation from a pathologist. Human PDAC organoids hT1 and hT108 did not generate tumors within one year, following transplantation, and could not be analyzed. Tumor differentiation analysis was performed blindly by a pathologist by scoring the percentage of differentiated or undifferentiated area per tumor. We then defined tumors as ‘differentiated’ if > 60% was differentiated, ‘undifferentiated’ if < 40% was differentiated and ‘mixed’ if 40-60% was differentiated. Analysis of epithelial/stroma proportion was done by calculating the percentage of ECAD^+^ area relative to the entire section. Stains and quantifications were performed blindly prior to plotting the data for visualization.

### Flow cytometry analyses

Tumors were collected at comparable tumor sizes and weights, and processed as previously described (5). Cells were blocked for 15 min on ice with CD16/CD32 Pure 2.4G2 (553142, BD Bioscience; RRID:AB_394657). For flow cytometric analysis of endothelial cells, immune cells, epithelial cells and iCAFs, myCAFs, CD90^+^ CAFs, CD90^-^ CAFs and apCAFs, cells were stained for 30 min on ice with anti-mouse CD31-PE/Cy7 (102418; BioLegend; RRID:AB_830757), CD45-PerCP/Cy5.5 (103132; BioLegend; RRID:AB_893344), CD326 (EpCAM)-AlexaFluor 488 (118210; BioLegend; RRID:AB_1134099), PDPN-APC/Cy7 (127418; BioLegend; RRID:AB_2629804), MHCII-BV785 (107645; BioLegend; RRID:AB_2565977), Ly6C-APC (128015; BioLegend; RRID:AB_1732087) and CD90-PE (ab24904; Abcam; RRID:AB_448474).

For flow cytometric analysis of CD56^+^ and CD49E^+^ CAFs of KPC cohorts 1 and 2 in nu/nu mice, cells were stained for 30 min on ice with anti-mouse CD56-APC (FAB7820A; Bio-Techne), CD49e-PE (557447; BD Biosciences; RRID:AB_396710), CD326 (EpCAM)-AlexaFluor 488 (118210; BioLegend; RRID:AB_1134099), PDPN-APC/Cy7 (127418; BioLegend; RRID:AB_2629804), CD45-BV785 (103149; BioLegend; RRID:AB_2564590), CD26 PerCP/Cy5.5 (45-0261-82; Thermo Fisher Scientific;, RRID:AB_1548738) and CD34 PE/Cy7 (25-0349-41; Thermo Fisher Scientific; RRID:AB_1963577). For flow cytometric analysis of CD105^+^ CAFs of KPC cohorts 1 and 2 in nu/nu mice, cells were stained for 30 min on ice with anti-mouse CD105-PE/Cy7 (120409; BioLegend; RRID:AB_1027702), CD45-PerCP/Cy5.5 (103132; BioLegend; RRID:AB_893344), CD326 (EpCAM)-AlexaFluor 488 (118210; BioLegend; RRID:AB_1134099), PDPN-APC/Cy7 (127418; BioLegend; RRID:AB_2629804), Ly6C BV785 (128041;BioLegend; RRID:AB_2565852) and CD140a PE (135906; BioLegend; RRID:AB_1953269).

For flow cytometric analysis of CD56^+^, CD49E^+^ and CD105^+^ CAFs of KPC cohort 3 in nu/nu mice and all other KPC and KvPC cohorts, cells were stained for 30 min on ice with anti-mouse CD56-APC (FAB7820A; Bio-Techne), CD49e-PE (557447; BD Biosciences; RRID:AB_396710), CD105-PE/Cy7 (120409; BioLegend; RRID:AB_1027702), CD326 (EpCAM)-AlexaFluor 488 (118210; BioLegend; RRID:AB_1134099), PDPN-APC/Cy7 (127418; BioLegend; RRID:AB_2629804), CD45-BV785 (103149; BioLegend; RRID:AB_2564590) and CD26 PerCP/Cy5.5 (45-0261-82; Thermo Fisher Scientific; RRID:AB_1548738).

For flow-cytometric analysis of macrophages and neutrophils of all KPC and KvPC cohorts, cells were stained for 30 min on ice with anti-mouse CD45-PerCP/Cy5.5 (103132; BioLegend; RRID:AB_893344), CD11b-PE/Cy7 (101215; BioLegend; RRID:AB_312798), Ly6C-Alexa488 (128021; BioLegend; RRID:AB_10640820), F4/80-BV785 (123141; BioLegend; RRID:AB_2563667), MHCII-APC/Cy7 (107627; BioLegend; RRID:AB_1659252), CD11c-APC (117309; BioLegend; RRID:AB_313779) and Gr1-PE (108407; BioLegend; RRID:AB_313372).

For flow-cytometric analysis of B cells, T cells and NK cells of KPC cohorts in C57BL/6J mice, cells were stained for 30 min on ice with anti-mouse CD45-PerCP/Cy5.5 (103132; BioLegend; RRID:AB_893344), CD19-PE/Cy7 (115520; Biolegend; RRID:AB_313655), NK1.1-BV785 (108749; Biolegend; RRID:AB_2564304), TCRβ-Alexa488 (109215; Biolegend; RRID:AB_493344); CD3ε-Alexa488 (100321; Biolegend; RRID:AB_389300), CD4-APC (100516; Biolegend; RRID:AB_312719) and CD8a-APC/Cy7 (100713; Biologend; RRID:AB_312752).

Cells were resuspended in PBS with DAPI and analyzed on a BD FACSymphony cell analyzer. Flow analyses were performed blindly using FlowJo 10.8.2 (RRID:SCR_008520) prior to plotting the data for visualization.

### Cell sorting of PDAC organoid/PSC co-cultures for RNA-sequencing

Sorting of PDAC organoid/PSC co-cultures was performed following 3.5 days culture in reduced media (i.e. 5% FBS DMEM). Following single cell digestion of co-cultures, cells were stained for 30 min on ice with anti-mouse CD326 (EpCAM)-PE (118205; BioLegend; RRID:AB_1134176) and PDPN-AlexaFluor 488 (156208; BioLegend; RRID:AB_2814080). Cells were resuspended in PBS with DAPI and sorted with a BD FACSMelody cell sorter.

### RNA-sequencing analyses of PDAC organoids and PSCs flow-sorted from co-cultures

RNA-seq data of KPC monocultures and co-cultures are available at the Gene Expression Omnibus (GEO) under the accession number GSE263080. RNA-seq data of KvPC monocultures and co-cultures are available at the GEO under the accession number GSE263081. Samples were collected in 1 mL of TRIzol Reagent (15596018; Invitrogen). RNA was extracted using the PureLink RNA mini kit (12183018A; Invitrogen). RNA concentration was measured using a Qubit and RNA quality was assessed on a TapeStation 4200 (Agilent) using the Agilent RNA ScreenTape kit. mRNA library preparations were performed using 55 μL of 10 ng/mL per sample (RNA integrity number, RIN > 8, except for one sample with RIN 7.2). Illumina libraries were then sequenced on 1 lane of SP flowcell on NovaSeq6000. FASTQ files were aligned, and the expression levels of each transcript were quantified using Salmon (v1.4.0) (37) with the annotation from ENSEMBL (GRCH39, release 109) with recommended settings. Transcript-level expression was loaded and summarized to the gene level by using tximport (38). Differential gene expression analysis was performed using DESeq2 (v2) (39) by applying lfcshrink function (40). The principal components for variance-stabilized data were estimated using plotPCA function, available in DESeq, and ggplot2 (https://ggplot2.tidyverse.org). Genes with adjusted p-value < 0.05 were selected as significantly differentiated between conditions. Following differential gene expression, genes were pre-ranked based on the negative logarithmic p-value and the sign of the log2 fold change. GSEA was performed using clusterprofiler against the Hallmark, Reactome, and C2 canonical pathway collection (C2.cp.v5.1) downloaded from the Molecular Signatures Database (MSigDB) (41).

#### NicheNet

NichenetR (42) was used to infer the ligands activity and their regulation potential of a sender group by considering the expression of downstream genes in the receiver group (i.e. target genes). Ligands were ranked based on their area under the precision-recall curve (AUPR) to prioritize ligands inferred to regulate the target genes in the receiver population. NicheNet was applied to infer the interaction between PDAC organoids and PSCs in KPC *Smad4* WT and KPC *Smad4* KO conditions. “Target genes” were defined by using significant differentially expressed genes (DEGs, p.adj < 0.05, log2FC > 1) in each condition of the receiver group, and the “ligands” were defined the same way in the sender group. All expressed genes in the dataset were considered as “background expressed genes”.

#### Transcription Factor (TF) activity analysis

TF activity analysis was performed with CollecTRI network (43) using a univariate linear model as implemented in decouplerR package (44). The stat column from the DESeq2 output was used as input. TFs with p-value < 0.05 were considered to have significant activity.

### RNA-sequencing analyses of *Smad4* KO and *Smad4* WT KPC PDAC tumors

Tumors from C57BL/6J mice were collected at comparable tumor sizes and weights. RNA-seq data of *Smad4* KO and *Smad4* WT KPC PDAC tumors are available at the GEO under the accession number GSE274684. Tumor pieces were snap frozen in liquid nitrogen and stored at -80 °C until processing. Samples were thawed and transferred to low bind tubes with 1 mL TRIzol reagent (15596018; Invitrogen) and 1 metal bead (69989; Qiagen). Samples were then homogenized at frequency 30/s for up to 6 min using a TissueLyser II (Qiagen). RNA was extracted as above. mRNA library preparations were performed using 1 mg of RNA per sample (RIN 5-9). Illumina libraries were sequenced on 1 lane of SP flowcell on a NovaSeq6000. RNA-seq analysis was performed as above although the transcript annotation used was GRCH39 (release 109) from ENSEMBLE.

### Single-cell RNA-sequencing analyses of PDAC tumors

Tumors from nu/nu mice were collected at comparable tumor sizes and weights. scRNA-seq data of KPC tumors are available at the GEO under the accession number GSE262879. scRNA-seq data of KvPC tumors are available at the GEO under the accession number GSE262878. Cell Ranger (10x Genomics) workflow (45) was used to align FASTQ files to GRCh38 (mm10) mouse transcriptome reference to generate the raw counts of gene expression quantities. For the KPC dataset, the reference genome was modified by adding *Gfp* to it. From the raw counts of each sample, the SOLO tool that is implemented in scvi-tools was used to estimate doublets to be removed (46). The samples were integrated, and batch effect was removed using scvi-tools (47). Scanpy workflow (48) was used for dimension reductions, clustering and defining markers of each cluster. Cells that have the percentage of mitochondrial genes more than 5% were filtered out. Unique molecular identifiers (UMI) were normalized to 10,000 counts and Leiden graph clustering were used for unsupervised clustering to identify the cell populations with similar transcriptomic profile. For dimension reduction we built the top 30 principal components (PC) and nearest neighbours’ graph (k = 10) on 2,000 highly variable genes. Uniform manifold approximation and projection (UMAP) were used to visualize the datasets in 2-dimentional space. Markers of each cluster were defined using “rank_genes_groups” function from Scanpy.

#### Copy Number Variation (CNV) analysis

A python implementation of inferCNV of the Trinity CTAT Project (https://github.com/broadinstitute/inferCNV) was used to estimate the copy number status in each cell type. We used the fibroblast cells as a reference key and a 250-genes window size.

#### Abundance test

We used Milo (49) python framework to compute differentially abundant neighbourhoods within the cell type clusters across conditions (i.e. *Smad4* KO vs *Smad4* WT). The following parameters were used k = 10, min proportion (p = 0.1) and dimensionality (d = 20). SpatialFDR < 0.1 was used for significance.

#### Pseudobulk

For DE analysis we pooled all cells within a specific cell type by summing the gene expression of each gene to create a pseudo-bulk expression profile of each sample. DESeq2 uses the pseudo-bulk data to detect the differences between two sample groups. To generate the pseudo-bulk profile and perform DEA, we used the python implementation of decoupler R package (44) with the default options. GSEA was performed as with bulk RNA-seq data.

#### CellChat

CellChat R package (50) was used with the recommended setting to infer and visualize the cell-cell communication between specific cell types in scRNA-seq data.

#### NicheNet

NicheNet (42) was used to infer the ligands activity and their regulation potential of a sender group by considering the expression of downstream genes in the receiver group (i.e. target genes). “Target genes” were defined based on the DEGs using findmarkers function from Seurat in the receiver cell clusters between two conditions (e.g. KO vs WT) using MAST (51) (*P* value < 0.05, logFC > 0.25). Genes that were expressed by 5% of the receiver cell cluster were considered as background genes. “Ligands” were defined based on the DEGs using findmarkers function in the sender cell clusters between the same two conditions (e.g. KO vs WT) using MAST (*P* value < 0.05, logFC > 0.25).

### Quantitative PCR analyses

Media was removed and cells were resuspended in TRIzol. RNA was prepared using a standard TRIzol/chloroform extraction method. Briefly, samples in TRIzol were transferred to 5PRIME phase lock gel tubes (2302830, VWR International) and mixed with chloroform before centrifuging for 15 min. The supernatant was then centrifuged for 10 min with isopropanol and glycogen (R0551, Fisher Scientific). Pellets were washed in ice-cold 75% ethanol and centrifuged again. Samples were resuspended in RNAse-free water and RNA concentration was quantified using a NanoDrop (ThermoFisher). Reverse transcription was performed with 100 ng -1 μg of RNA using TaqMan reverse transcription reagents (N808-0234; Applied Biosystems) and following the manufacturer’s instructions. qPCR was performed using TaqMan master mix (4440040; Applied Biosystems) and TaqMan gene-specific probes (Applied Biosystems) on a QuantStudio 6 Flex Real-Time PCR system. Gene expression was normalised to *Hprt* housekeeping gene.

### Enzyme Linked Immunosorbent Assay (ELISA)

Organoid-conditioned media samples were collected from organoids grown in 5% FBS DMEM for 3-5 days. ELISA assays were used to detect murine IL-1α (MLA00; R&D Systems) and murine TGF-β (BMS608-4, Thermo Fisher Scientific). Media was assayed according to the manufacturer’s instructions.

### Statistical analysis

GraphPad Prism software (RRID:SCR_002798), customized R and python scripts were used for graphical representation of data. Statistical analysis was performed using non-parametric Mann-Whitney test, unpaired or paired Student’s *t* test or chi-square test. All statistical details of experiments are specified in the figure legends and/or panel figures, including the number of technical and biological replicates, and how significance was defined.

## Results

### *Smad4* loss impacts the immune TME and malignant-stromal crosstalk in KPC PDAC

**Figure 1:**
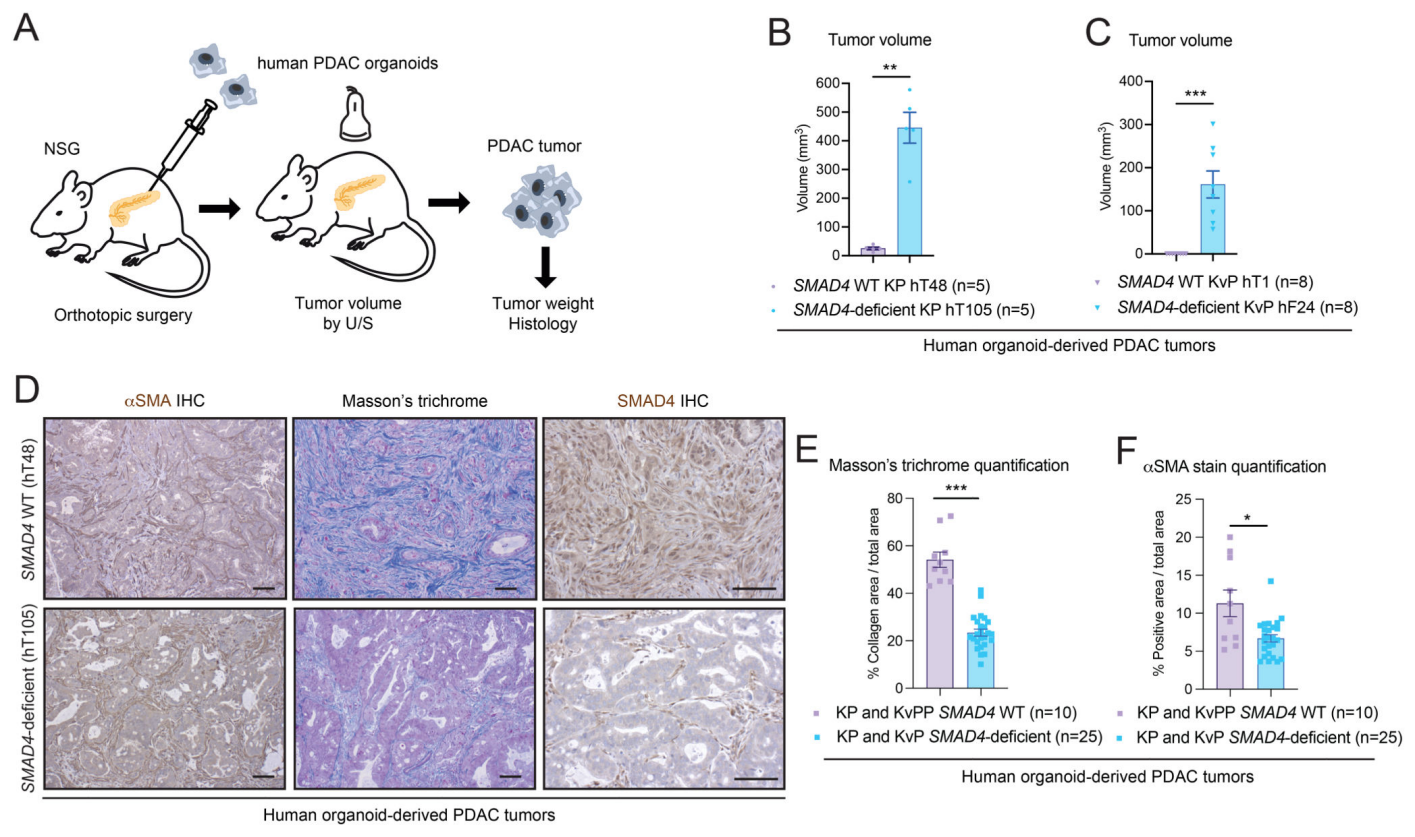
*SMAD4*-deficient human organoid-derived PDAC tumors have less fibrosis than *SMAD4* WT tumors. **(A)** Schematic of analyses of orthotopically-grafted human organoid-derived pancreatic ductal adenocarcinoma (PDAC) models in NOD SCID gamma (NSG) mice. U/S, ultrasound-based imaging. **(B-C)** Volumes, measured by U/S, of tumors derived from the transplantation of KP (58 days post-transplant) **(B)** or KvP (64 days post-transplant) **(C)** *SMAD4* wild-type (WT) or *SMAD4*-deficient human PDAC organoids with *KRAS*^G12D^ or *KRAS*^G12V^ mutation, respectively. **(D)** Representative SMAD4, Masson’s trichrome and alpha smooth muscle actin (αSMA) stains in *SMAD4* WT or *SMAD4*-deficient human organoid-derived KP PDAC. Scale bars, 50 μm. **(E-F)** Quantification of Masson’s trichrome **(E)** and αSMA **(F)** stains in *SMAD4* WT or *SMAD4*-deficient human organoid-derived PDAC. For **C, E** and **F**, results show mean ± SEM. *, *P* < 0.05, **, *P* < 0.01; ***, *P* < 0.001, Mann-Whitney test.

Human PDAC tumors display remarkable inter-tumoral clinical, histological, and genetic heterogeneity, rendering it difficult to understand how mutations cooperate to drive tumorigenesis and remodel the TME (Supplementary Fig. 1A-B; Supplementary Table S1). Therefore, to start to dissect the relationship between malignant cell genotype and stromal phenotype, we engrafted patient-derived PDAC organoids harboring *KRAS^G12D^* (K) or *KRAS*^*G12V*^ (Kv) with *TP53*-mutant (P) or *TP53*-loss (PP), and *SMAD4* WT or *SMAD4* deficient status into the pancreata of NOD SCID gamma (NSG) mice (35) (Fig. 1A; Supplementary Fig. 1C-D; Supplementary Table S2). *SMAD4* loss accelerated PDAC progression in *KRAS^G12D^* and *KRAS*^*G12V*^ *TP53*-mutant PDAC (i.e. KP and KvP) (Fig. 1B-C; Supplementary Fig. 1E-F). *SMAD4* loss also increased metastasis formation in KP and KvP PDAC compared to *SMAD4* WT KP and KvPP tumors of similar size (Supplementary Fig. 1G-H). Together, these results agree with patient data showing that SMAD4 loss typically drives an aggressive PDAC phenotype, although analysis of isogenic human organoid-derived models would better address this (29,52). Finally, *SMAD4* loss decreased the desmoplasia − measured as collagen deposition and alpha smooth muscle actin (αSMA) levels (Fig. 1D-F; Supplementary Fig. 1I). To further explore the impact of SMAD4 loss on PDAC progression, overcome inter-patient heterogeneity and study bi-directional malignant-stromal crosstalk, we generated murine isogenic organoids with *Smad4* WT or deleted.

**Figure 2:**
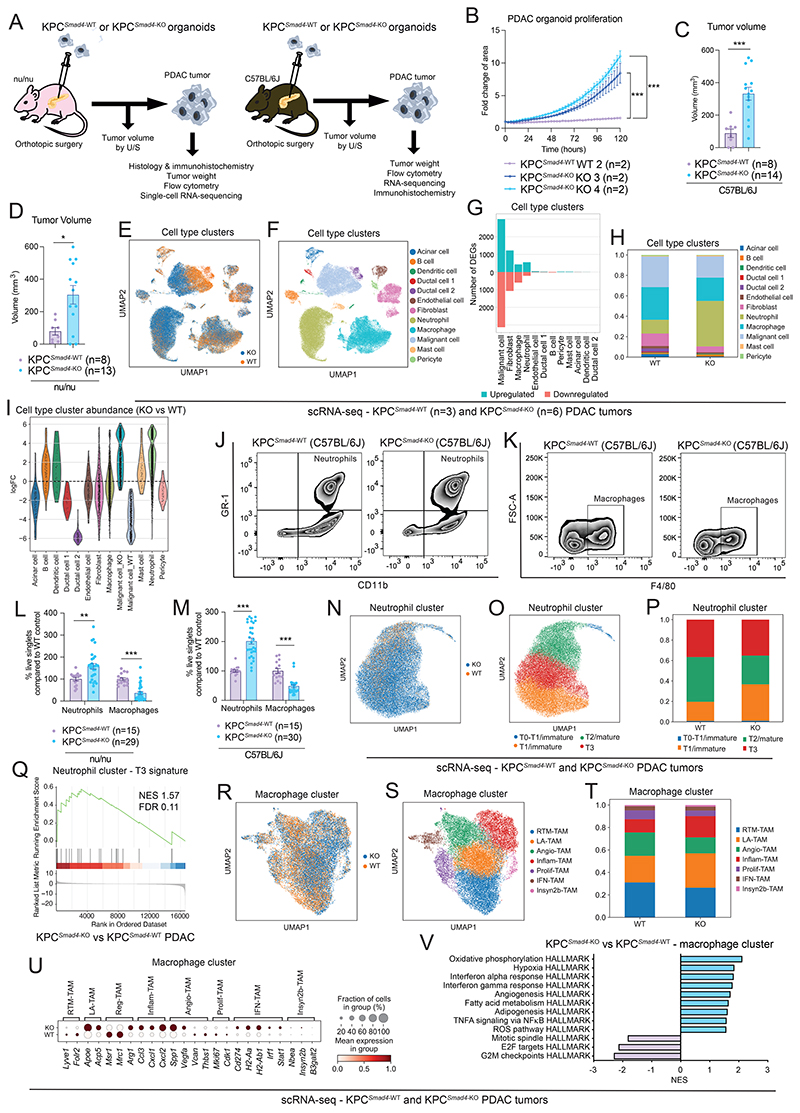
*Smad4* loss impacts the immune TME in KPC PDAC. **(A)** Schematic of analyses of KPC (i.e. *Kras*^G12D^ p53-mutant) orthotopically-grafted organoid-derived PDAC models in nu/nu (left) or C57BL/6J (right) mice. **(B)** Proliferation curves of KPC^*Smad4*-WT^ or KPC^*Smad4*-KO^ (KO3 and KO4) PDAC organoids cultured for 120 hours in reduced media (i.e., 5% FBS DMEM). Data were normalized to the first measurement (3 hours post-plating). Results show mean ± SEM of n=2 biological replicates (with n=4 technical replicates each). ***, *P* < 0.001, Mann-Whitney test calculated for the last time point. **(C)** Volumes of tumors derived from transplantation of KPC^*Smad4*-WT^ or KPC^*Smad4*-KO^ organoids in C57BL/6J mice measured by U/S. Results show mean ± SEM from 2 separate experiments, each with 1 WT group and 2 groups of KO pools from 2 different guides (32 days (n= 4 WT and n= 8 KO) or 21 days (n= 4 WT and n= 6 KO) post-transplant). ***, *P* < 0.001, Mann-Whitney test. **(D)** Volumes of tumors derived from transplantation of KPC^*Smad4*-WT^ or KPC^*Smad4*-KO^ PDAC organoids in nu/nu mice measured by U/S. Results show mean ± SEM from 2 separate experiments, each with 1 WT group and 2 groups of KO pools from 2 different guides (25 days (n= 4 WT and n= 7 KO) or day 21 days (n= 4 WT and n= 6 KO) post-transplant). *, *P* < 0.05, Mann-Whitney test. **(E-F)** Uniform manifold approximation and projection (UMAP) plot shows cell clusters from KPC^*Smad4*-WT^ (n=3) or KPC^*Smad4*-KO^ (n=6) tumors analyzed by single-cell RNA-sequencing (scRNA-seq), color-coded by **(E)** genotype or **(F)** cell type. **(G)** Upregulated and downregulated differentially expressed genes (DEGs) in each cell type identified by pseudobulk analysis from scRNA-seq of KPC^*Smad4*-WT^ or KPC^*Smad4*-KO^ tumors. False discovery rate (FDR) < 0.05. **(H)** Cell type contribution in KPC^*Smad4*-WT^ or KPC^*Smad4*-KO^ tumors. **(I)** Violin plots showing the distribution of groups of nearest neighbor cells from different cell type clusters upon the log-fold change between KPC^*Smad4*-KO^ vs KPC^*Smad4*-WT^ conditions computed with Milo. The malignant cell cluster was divided by cells from *Smad4* WT or *Smad4* KO tumors to clarify the directionality of abundance. **(J-K)** Representative flow plots of **(J)** neutrophils (CD45^+^CD11b^+^Gr1^+^) and **(K)** macrophages (CD45^+^Gr1^-^CD11b^+^F4/80^+^) from KPC^*Smad4*-WT^ or KPC^*Smad4*-KO^ tumors in C57BL/6J mice. **(L-M)** Flow cytometric analysis of neutrophils (CD45^+^CD11b^+^Gr1^+^) and macrophages (CD45^+^Gr1^-^CD11b^+^F4/80^+^) from live singlets in KPC^*Smad4*-WT^ or KPC^*Smad4*-KO^ tumors in **(L)** nu/nu or **(M)** C57BL/6J mice. Results show mean ± SEM from 3 separate experiments, each with 1 WT group and 2 groups of KO pools from 2 different guides. **, *P* < 0.01; ***, *P* < 0.001, Mann-Whitney test. **(N-O)** UMAP plot of neutrophils from KPC^*Smad4*-WT^ or KPC^*Smad4*-KO^ tumors analyzed by scRNA-seq, color-coded by **(N)** genotype or **(O)** sub-cluster. **(P)** Sub-cluster contribution in neutrophils of KPC^*Smad4*-WT^ or KPC^*Smad4*-KO^ tumors. **(Q)** Gene set enrichment analysis (GSEA) of T3 neutrophil signature in neutrophils from KPC^*Smad4*-KO^ PDAC compared to KPC^*Smad4*-WT^ PDAC. The signature from Ng et al (13) is significantly upregulated. NES, normalized enrichment score. **(R-S)** UMAP plot of macrophages from KPC^*Smad4*-WT^ or KPC^*Smad4*-KO^ tumors analyzed by scRNA-seq, color-coded by **(R)** genotype or **(S)** sub-cluster. RTM-TAM, resident-tissue macrophage-like tumor-associated macrophage (TAM); LA-TAM, lipid-associated TAM; Angio-TAM, pro-angiogenic TAM; Inflam-TAM, inflammatory cytokine-enriched TAM; Prolif-TAM, proliferating TAM; IFN-TAM, interferon-primed TAM. Macrophage subtypes are from Ma et al (12). **(T)** Sub-cluster contribution in macrophages of KPC^*Smad4*-WT^ or KPC^*Smad4*-KO^ tumors. **(U)** Dot plot visualization of scaled average expression of macrophage subtype markers in macrophages from KPC^*Smad4*-WT^ or KPC^*Smad4*-KO^ tumors analyzed by scRNA-seq. Color intensity represents expression level and dot size represents the percentage of expressing cells. **(V)** Selected significantly upregulated (i.e. NES > 1.50 and FDR < 0.25) and downregulated (i.e. NES < -1.50 and FDR < 0.25) pathways identified by GSEA of macrophages from KPC^*Smad4*-KO^ compared to KPC^*Smad4*-WT^ tumors, assessed by pseudobulk analysis from the scRNA-seq dataset.

*KRAS^G12D^* is the most frequent *KRAS* mutant allele in PDAC patients (23). Thus, to further study the role of KRAS and SMAD4 status in PDAC, we deleted *Smad4* from KPC GEMM-derived organoids (24,32,36) (Supplementary Fig. 2A-D). Isogenic *Smad4* knockout (KO) KPC (hereafter, KPC^*Smad4*-KO^) organoids grew faster than *Smad4* WT KPC (hereafter, KPC^*Smad4*-WT^) organoids both *in vitro* and as orthotopically-grafted tumors in immunocompromised nu/nu or immunocompetent C57BL/6J mice (Fig. 2A-D; Supplementary Fig. 2E-F).

To understand how SMAD4 loss in KPC malignant cells might impact the TME, we generated single-cell RNA-sequencing (scRNA-seq) profiles of similar size KPC^*Smad4*-WT^ and KPC^*Smad4*-KO^ tumors in which the malignant cells were engineered to express green fluorescent protein (GFP) as part of one of our CRISPR strategies (Fig. 2E-F; Supplementary Fig. 2G-K; Supplementary Table S3). Similar proportions of cell populations were previously found in similarly processed human PDAC tumors (3). scRNA-seq profiles of malignant cells could be readily distinguished by the expression of *Gfp* transcripts and inferred copy number variations (CNVs; Supplementary Fig. 2L-M). *Smad4* deletion markedly altered the transcriptomes of KPC^*Smad4*-KO^ relative to KPC^*Smad4*-WT^ malignant cells (6,121 differentially expressed genes, DEGs, false discovery rate, FDR < 0.05) (Fig. 2G). Remarkably, scRNA-seq profiles also revealed significant and selective changes in the transcriptomes and proportions of CAFs, macrophages and neutrophils in KPC^*Smad4*-KO^ relative to KPC^*Smad4*-WT^ tumors (Fig. 2G-I). Thus, SMAD4 loss in murine PDAC malignant cells impacts both CAFs and innate immune cells.

Additional analyses confirmed an increase in neutrophils and a decrease in macrophages in KPC^*Smad4*-KO^ relative to KPC^*Smad4*-WT^ tumors of similar size and showed a significant decrease in natural killer (NK) cells (Fig. 2J-M; Supplementary Fig. 2G and 2N-U). Of note, C57BL/6J mice with KPC^*Smad4*-KO^ PDAC showed more metastases than KPC^*Smad4*-WT^ models with comparable tumor size (Supplementary Fig. 2O and 2V). Analysis of neutrophils identified previously described immature T1, mature T2 and tumor-promoting T3 populations (13), as well as a small subset expressing T1 markers and *Mpo, Ly6g* and *Ly6c1* transcripts (Fig. 2N-P; Supplementary Fig. 3A). The gene signature of T3 neutrophils was upregulated in KPC^*Smad4*-KO^ tumors (Fig. 2Q; Supplementary Fig. 3B). Additionally, analysis of macrophages identified recently described sub-types of tumor-associated macrophages (TAMs) (12), as well as a small cluster, which we named Insyn2b-TAMs based on marker expression (Fig. 2R-T; Supplementary Fig. 3C). The expression of genes associated with interferon-primed TAMs (IFN-TAMs), lipid-associated TAMs (LA-TAMs) and inflammatory cytokine-enriched TAMs (Inflam-TAMs) were upregulated in KPC^*Smad4*-KO^ relative to KPC^*Smad4*-WT^ PDAC, while proliferating TAMs (Prolif-TAMs) were reduced (Fig. 2U-V).

**Figure 3:**
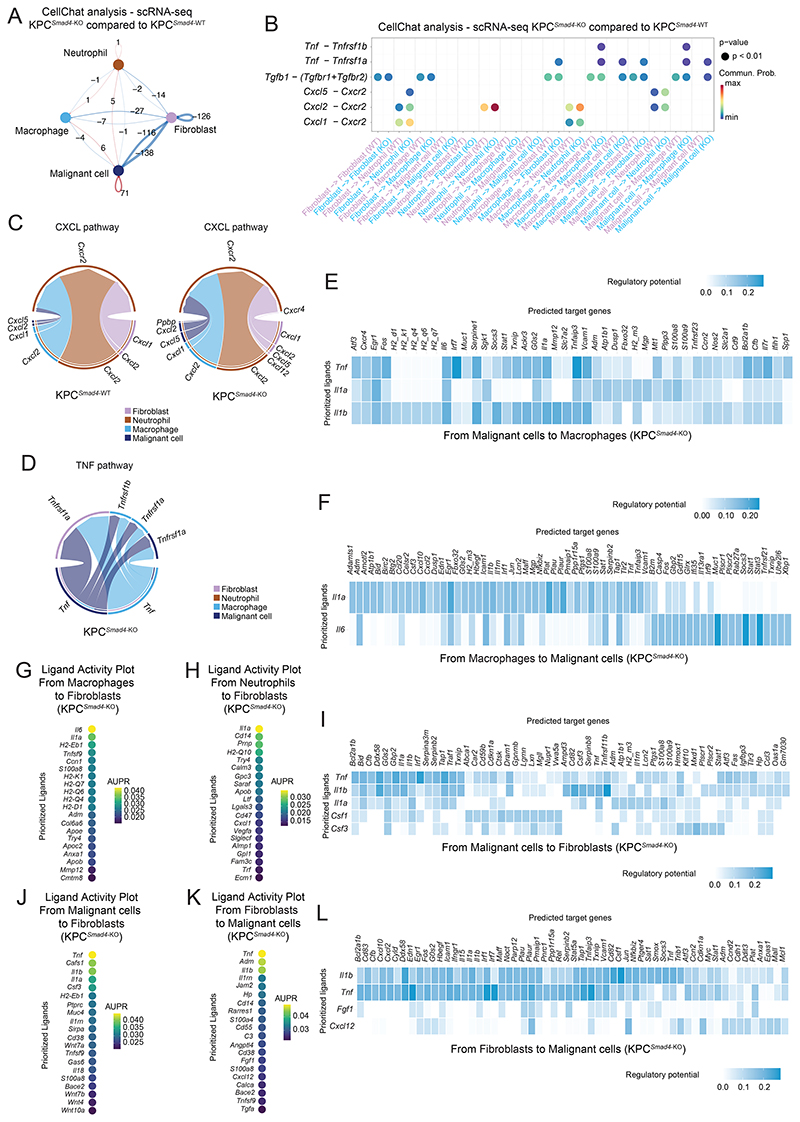
*Smad4* loss impacts malignant-stromal crosstalk in KPC PDAC. **(A)** Cell-cell communication analysis using CellChat showing the number of connections lost (blue) or gained (red) between malignant cells, fibroblasts, macrophages and neutrophils in KPC^*Smad4*-KO^ (n=6) compared to KPC^*Smad4*-WT^ (n=3) tumors, as assessed by scRNA-seq. **(B)** Selected ligand-receptor interactions and their strength based on CellChat analysis between malignant cells, fibroblasts, macrophages and neutrophils in KPC^*Smad4*-KO^ tumors compared to KPC^*Smad4*-WT^ tumors. Commun. Prob., communication probability. **(C-D)** Selected pathways with significantly different connections between malignant cells, fibroblasts, macrophages, and neutrophils in KPC^*Smad4*-KO^ tumors compared to KPC^*Smad4*-WT^ tumors. **(E-F)** Ligand-target heatmaps show top selected ligands of **(E)** malignant cells inferred to regulate target genes in macrophages and **(F)** macrophages inferred to regulate target genes in malignant cells in KPC^*Smad4*-KO^ PDAC, as assessed by NicheNet analysis of scRNA-seq. **(G-H)** Ligand activity plots show the top ligands of **(G)** macrophages or **(H)** neutrophils inferred to regulate target genes in fibroblasts in KPC^*Smad4*-KO^ PDAC, as assessed by NicheNet analysis. AUPR, area under the precision-recall curve. **(I)** Ligand-target heatmap shows top selected ligands of malignant cells inferred to regulate target genes in fibroblasts in KPC^*Smad4*-KO^ PDAC, as assessed by NicheNet analysis. **(J)** Ligand activity plot shows the top ligands of malignant cells inferred to regulate target genes in fibroblasts in KPC^*Smad4*-KO^ PDAC, as assessed by NicheNet analysis. **(K)** Ligand activity plot shows the top ligands of fibroblasts inferred to regulate target genes in malignant cells in KPC^*Smad4*-KO^ PDAC, as assessed by NicheNet analysis. **(L)** Ligand-target heatmap shows top selected ligands of fibroblasts inferred to regulate target genes in malignant cells in KPC^*Smad4*-KO^ PDAC, as assessed by NicheNet analysis.

CellChat analysis (50), which infers ligand-receptor interactions and patterns of cell-cell communication, identified potential changes in communication among malignant cells, neutrophils, macrophages and CAFs in KPC^*Smad4*-KO^ relative to KPC^*Smad4*-WT^ PDAC (Fig. 3A). These included a role for malignant cell-derived tumor necrosis factor (TNF) in dictating macrophage composition, as well as macrophage- and malignant cell- derived (C-X-C motif) ligands 1 and 2 (CXCL1 and CXCL2) in neutrophil recruitment in KPC^*Smad4*-KO^ PDAC (Fig. 3B-D). Fibroblasts in KPC^*Smad4*-KO^ tumors also appeared to be more involved in neutrophil recruitment via the CXCR2 pathway, as previously found in iCAF-rich PDAC (18). Of note, the most affected interaction in KPC^*Smad4*-KO^ PDAC appeared to be between malignant cells and fibroblasts, in line with CAFs being the most impacted stromal cell population upon SMAD4 loss (2,294 DEGs, FDR < 0.05; Fig. 3A and 2G).

To further investigate the crosstalk of these four cell populations, we applied NicheNet, which infers ligand-target relationships among cell types (42). This analysis suggested predominant TGF-β signaling in KPC^*Smad4*-WT^ tumors and interleukin 1 (IL-1) signaling in KPC^*Smad4*-KO^ tumors from malignant cells and fibroblasts to macrophages (Fig. 3E; Supplementary Fig. 3D-F; Supplementary Table S4). This analysis also confirmed the role of KPC^*Smad4*-KO^ malignant cell-produced TNF in shaping macrophage composition and inferred a role for macrophage-produced IL-1α and IL-6 in shaping malignant cells and fibroblasts (Fig. 3E-G; Supplementary Fig. 3G). Finally, it suggested that IL-1α from neutrophils contributes to the increase in iCAFs and highlighted a role of TNF and IL-1β in reciprocal malignant cell-fibroblast crosstalk in KPC^*Smad4*-KO^ tumors (Fig. 3H-L; Supplementary Fig. 3H-L).

These analyses show that loss of SMAD4 in murine KRAS^G12D^ p53-mutant PDAC malignant cells shapes the immune TME, and they suggest that CAFs are also profoundly impacted.

### *Smad4* loss drives a fibro-inflammatory stroma in KPC PDAC

**Figure 4:**
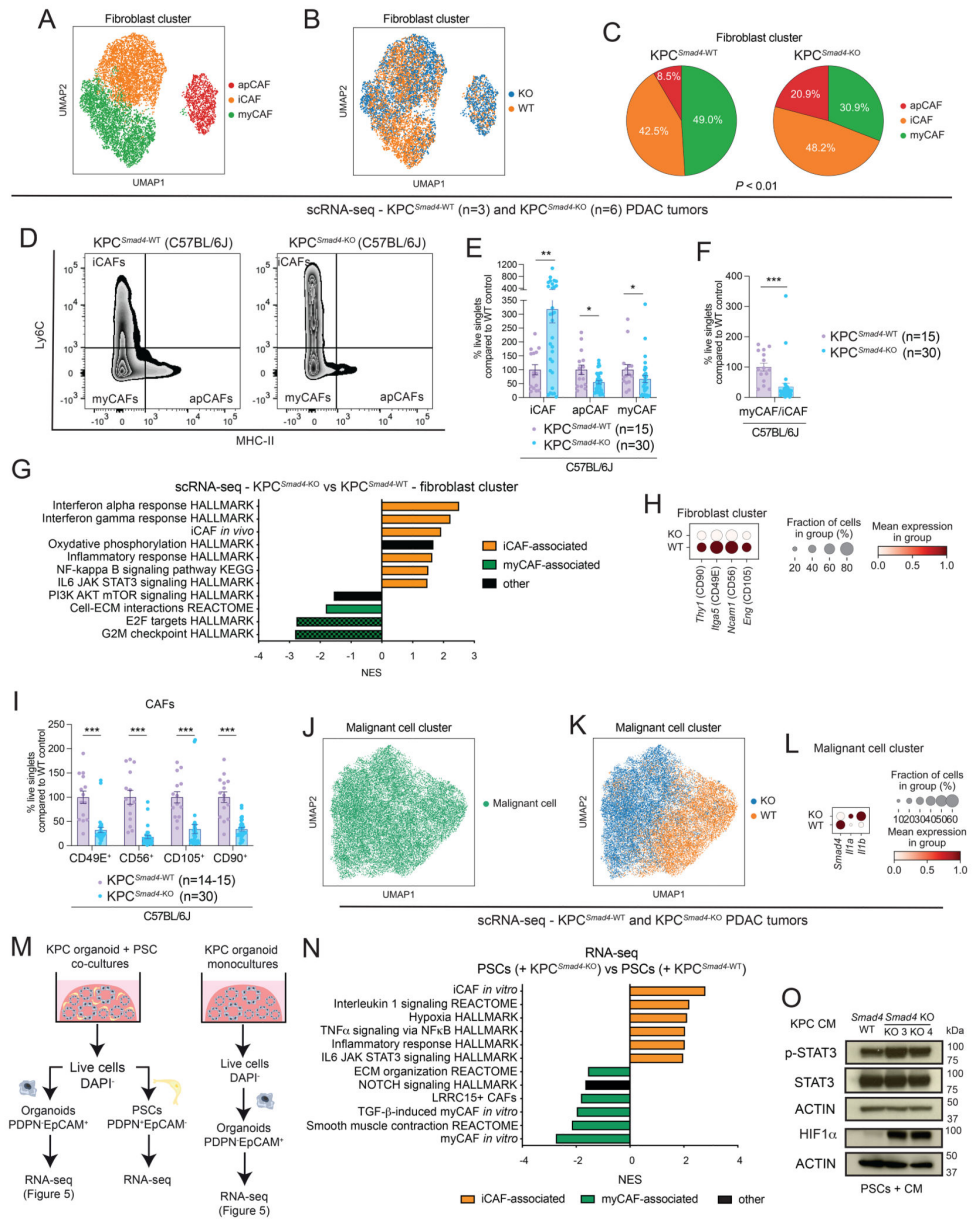
*Smad4* loss drives a fibro-inflammatory stroma in KPC PDAC. **(A-B)** UMAP plots showing the cell cluster of cancer-associated fibroblasts (CAFs) from KPC^*Smad4*-WT^ (n=3) or KPC^*Smad4*-KO^ (n=6) PDAC analyzed by scRNA-seq, color-coded by sub-cluster **(A)** or genotype **(B). (C)** Pie charts showing proportions of different CAF clusters from KPC^*Smad4*-WT^ or KPC^*Smad4*-KO^ tumors. *P* < 0.01, chi-square test. **(D)** Representative flow plots of Ly6C^-^MHCII^-^ myCAFs, Ly6C^+^MHCII^-^ iCAFs and Ly6C^-^MHCII^+^ apCAFs from KPC^*Smad4*-WT^ or KPC^*Smad4*-KO^ tumors in C57BL/6J mice. **(E-F)** Flow cytometric analyses of **(E)** myCAFs (Ly6C^-^MHCII^-^ CAFs), iCAFs (Ly6C^+^MHCII^-^ CAFs) and apCAFs (Ly6C^-^MHCII^+^ CAFs) and **(F)** myCAF/iCAF ratio from live singlets in KPC^*Smad4*-WT^ or KPC^*Smad4*-KO^ tumors in C57BL/6J mice. Results show mean ± SEM from 3 separate experiments, each with 1 WT group and 2 groups of KO pools from 2 different guides. *, *P* < 0.05, **, *P* < 0.01, ***, *P* < 0.001, Mann-Whitney test. **(G)** Selected significantly upregulated (i.e. NES > 1.50, FDR < 0.25; apart for the IL6 JAK STAT3 signaling HALLMARK with NES=1.47) and downregulated (i.e. NES < -1.50, FDR < 0.25) pathways identified by GSEA of CAFs from KPC^*Smad4*-KO^ compared to KPC^*Smad4*-WT^ tumors, as assessed by pseudobulk analysis from scRNA-seq. The *in vivo* iCAF signature is from Elyada et al (3). **(H)** Dot plot visualization of the scaled average expression of myCAF-enriched markers in CAFs from KPC^*Smad4*-WT^ or KPC^*Smad4*-KO^ tumors, as analyzed by scRNA-seq. Color intensity represents expression level and dot size represents the percentage of expressing cells. **(I)** Flow cytometric analyses of CD90^+^, CD49E^+^, CD56^+^ and CD105^+^ CAFs from live singlets in KPC^*Smad4*-WT^ or KPC^*Smad4*-KO^ tumors in C57BL/6J mice. Results show mean ± SEM from 3 separate experiments, each with 1 WT group and 2 groups of KO pools from 2 different guides. ***, *P* < 0.001, Mann-Whitney test. **(J-K)** UMAP plots showing the malignant cell cluster from KPC^*Smad4*-WT^ or KPC^*Smad4*-KO^ tumors analyzed by scRNA-seq **(J)**. Different genotypes are color-coded **(K). (L)** Dot plot visualization of the scaled average expression of *Smad4, Il1a* and *Il1b* in malignant cells from KPC^*Smad4*-WT^ or KPC^*Smad4*-KO^ tumors, as analyzed by scRNA-seq. Color intensity represents expression level and dot size represents the percentage of expressing cells. **(M)** Schematic of flow-sorting strategy of pancreatic stellate cells (PSCs) and KPC^*Smad4*-WT^ or KPC^*Smad4*-KO^ PDAC organoids from monocultures or co-cultures for RNA-sequencing (RNA-seq). **(N)** Selected significantly upregulated and downregulated pathways identified by GSEA of PSCs cultured with KPC^*Smad4*-KO^ organoids (n=10) compared to PSCs cultured with KPC^*Smad4*-WT^ organoids (n=5). The *in vitro* iCAF and myCAF signatures are from Öhlund et al (4). The TGF-β-induced myCAF *in vitro* signature is from Mucciolo and Araos Henríquez et al (11). The LRRC15^+^ CAF signature is from Dominguez et al (7). **(O)** Western blot analysis of phospho-STAT3 (p-STAT3), STAT3 and hypoxia inducible factor 1 alpha (HIF-1α) in PSCs cultured for 4 days in PDAC organoid conditioned media (CM) from KPC^*Smad4*-WT^ or KPC^*Smad4*-KO^ organoids. ACTIN, loading controls.

While fibrosis and epithelial/stroma proportion were not affected, collagen deposition was reduced in KPC^*Smad4*-KO^ tumors, further suggesting that malignant cell SMAD4 status impacts CAF composition in murine PDAC (Supplementary Fig. 4A-E). Indeed, proportions of iCAFs, myCAFs and apCAFs defined by scRNA-seq profiles significantly differ between KPC^*Smad4*-KO^ and KPC^*Smad4*-WT^ tumors (Fig. 4A-C; Supplementary Fig. 4F). Of note, scRNA-seq analysis inferred a decrease in the myCAF/iCAF ratio in KPC^*Smad4*-KO^ tumors, which was confirmed by flow cytometry (Fig. 4C-F; Supplementary Fig. 4G-H). In keeping with these findings, iCAF-associated pathways were upregulated while proliferation-associated pathways, which were shown to be enriched in myCAFs (5), were downregulated in KPC^*Smad4*-KO^ tumors (Fig. 4G). Additionally, abundance of MHCII-expressing apCAFs was lower in KPC^*Smad4*-KO^ tumors (Fig. 4D-E; Supplementary Fig. 4G). PDAC apCAFs have been identified by both transcriptome signature and the expression of MHCII protein (3,10). Interestingly, SMAD4 loss in malignant cells resulted in the presence of CAFs that upregulate the iCAF signature but retain an apCAF transcriptome, while being negative for MHCII protein (Supplementary Fig. 4I).

We further interrogated the heterogeneity of myCAFs by using CD90 (encoded by *Thy1*), which marks a myCAF subset (11), as well as CD105 (*Eng*, and a CAF-lineage marker (6)), CD49E (*Itga5*) and CD56 (*Ncam1*). CD105, CD49E and CD56 have not been described previously as myCAF markers but were enriched in myCAFs by scRNA-seq (Supplementary Fig. 4F). Each of these markers were significantly downregulated in CAFs in KPC^*Smad4*-KO^ tumors, while tumor-promoting CD90^-^ myCAFs were increased (Fig. 4H-I; Supplementary Fig. 4J-N). Furthermore, malignant cell profiles in KPC^*Smad*-KO^ tumors expressed high levels of *Il1a* and *Il1b*, which direct iCAF formation (5) (Fig. 4J-L). Thus, SMAD4 loss in KPC cells potentially shapes PDAC CAF composition through IL-1 signaling.

To further deconvolute changes in malignant cell-fibroblast crosstalk upon SMAD4 loss, we leveraged a co-culture model of PDAC organoids and pancreatic stellate cells (PSCs) (4). PSCs are precursors of CAFs and model iCAFs and myCAFs *in vitro* (4,5,9,11). KPC^*Smad4*-KO^ and KPC^*Smad4*-WT^ organoids cultured either alone or with PSCs were flow-sorted and analyzed by RNA-seq (Fig. 4M; Supplementary Fig. 4O; Supplementary Tables S5-S6). In keeping with our *in vivo* observations, iCAF markers and associated pathways were increased, while myCAF markers and associated pathways were decreased in KPC^*Smad4*-KO^/PSC co-cultures (Fig. 4N; Supplementary Fig. 4P). Furthermore, the JAK/STAT pathway and hypoxia signature, which are iCAF features (5,53), were enriched in PSCs co-cultured with KPC^*Smad4*-KO^ organoids (Fig. 4N). These results were validated by upregulation of phospho-STAT3 (p-STAT3) and hypoxia-inducible factor 1-alpha (HIF-1α) levels in PSCs cultured with conditioned media (CM), which induces the iCAF phenotype (4,5), from KPC^*Smad4*-KO^ relative to KPC^*Smad4*-WT^ organoids (Fig. 4O). Thus, *Smad4* deletion in KRAS^G12D^ p53-mutant PDAC malignant cells induces an inflammatory phenotype in CAFs.

### *Smad4* loss upregulates IL-1 and JAK/STAT signaling in KPC PDAC

**Figure 5:**
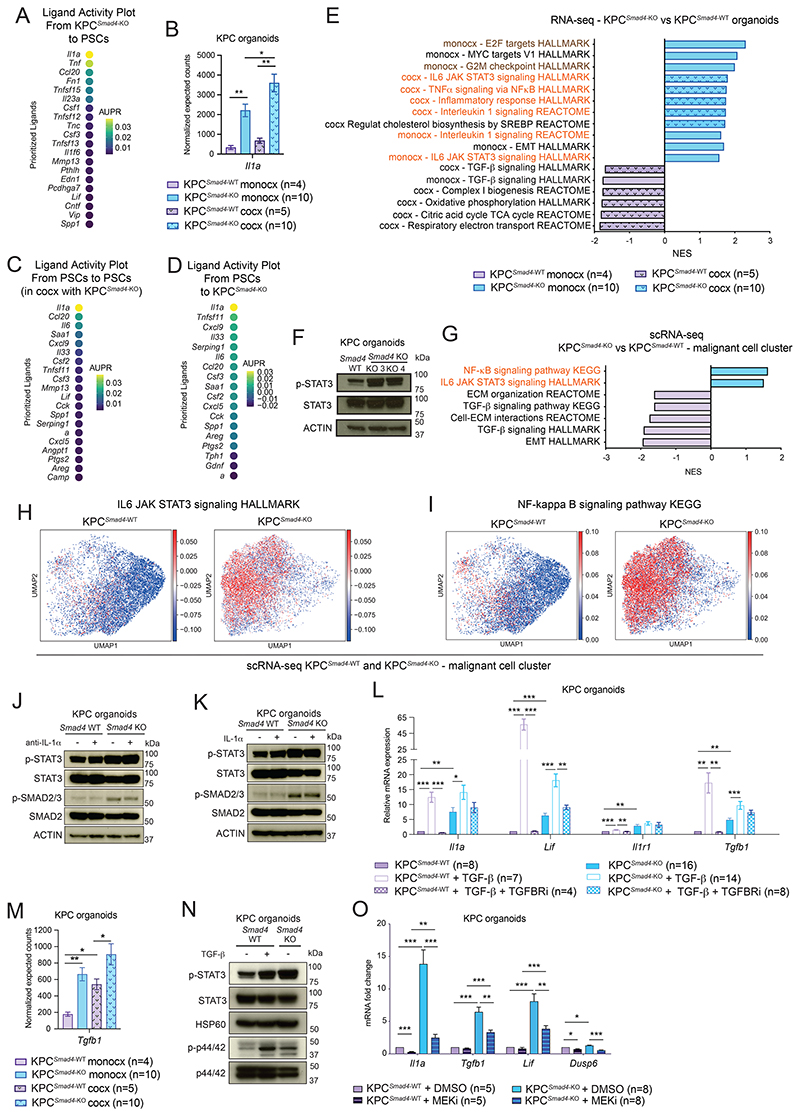
*Smad4* loss upregulates IL-1 and JAK/STAT signaling in KPC PDAC. **(A)** Ligand activity plot shows the top ligands of KPC^*Smad4*-KO^ PDAC organoids inferred to regulate target genes in co-cultured PSCs, as assessed by NicheNet analysis of RNA-seq. **(B)** RNA-seq expression of *Il1a* in KPC^*Smad4*-WT^ or KPC^*Smad4*-KO^ organoids flow-sorted from monocultures or co-cultures with PSCs. Results show mean ± SEM. *, *P* < 0.05, **, *P* < 0.01, Mann-Whitney test. **(C-D)** Ligand activity plots show the top ligands of PSCs in co-culture with KPC^*Smad4*-KO^ organoids inferred to regulate target genes in **(C)** PSCs or **(D)** co-cultured organoids, as assessed by NicheNet analysis. **(E)** Selected GSEA pathways significantly enriched or depleted in KPC^*Smad4*-KO^ compared to KPC^*Smad4*-WT^ malignant cells flow-sorted from monocultures or co-cultures with PSCs. The smooth pattern indicates the monocultures. Inflammatory pathways are in orange. Proliferation-associated pathways are in brown. Cocx, co-culture; monocx, monoculture. **(F)** Western blot analysis of p-STAT3 and STAT3 in KPC^*Smad4*-WT^ or KPC^*Smad4*-KO^ organoids cultured in reduced media for 2 days. ACTIN, loading control. **(G)** Selected significantly upregulated or downregulated pathways identified by GSEA of malignant cells from KPC^*Smad4*-KO^ tumors (n=6) compared to malignant cells from KPC^*Smad4*-WT^ tumors (n=3), as assessed by pseudobulk analysis of scRNA-seq. Inflammatory pathways are in orange. **(H-I)** UMAP plots of malignant cells from KPC^*Smad4*-WT^ or KPC^*Smad4*-KO^ tumors colored by the NES of the HALLMARK IL6 JAK STAT3 signaling **(H)** or the KEGG NF-κB signaling **(I)** pathways, as analyzed by scRNA-seq. **(J-K)** Western blot analysis of p-STAT3, STAT3, phospho-SMAD2/3 (p-SMAD2/3) and SMAD2 in KPC^*Smad4*-WT^ or KPC^*Smad4*-KO^ organoids cultured for 3 days in reduced media with **(J)** 5 μg/mL anti-IL1α or isotype control, or **(K)** 10 ng/mL IL1-α. ACTIN, loading controls. **(L)** qPCR analysis of *Il1a, Lif, Il1r1*, and *Tgfb1* in KPC^*Smad4*-WT^ or KPC^*Smad4*-KO^ organoids cultured for 2 days in reduced media with or without 5-20 ng/mL TGF-β in the presence or absence of 2 μM TGFBR1 inhibitor A83-01 (TGFBRi). Results show mean ± SEM. *, *P* < 0.05; **, *P* < 0.01; ***, *P* < 0.001, paired and unpaired Student’s *t* test. **(M)** RNA-seq expression of *Tgfb1* in KPC^*Smad4*-WT^ or KPC^*Smad4*-KO^ organoids flow-sorted from monocultures or co-cultures with PSCs. Results show mean ± SEM. *, *P* < 0.05; **, *P* < 0.01, Mann-Whitney test. **(N)** Western blot analysis of p-STAT3, STAT3, phospho-p44/42 (p-p44/42) and p44/42 in KPC^*Smad4*-WT^ or KPC^*Smad4*-KO^ organoids cultured for 2 days in reduced media with 5 ng/mL TGF-β. HSP60, loading control. **(O)** qPCR analysis of *Il1a, Tgfb1, Lif*, and *Dusp6* (i.e. a MAPK MEK/ERK target) in KPC^*Smad4*-WT^ or KPC^*Smad4*-KO^ organoids cultured for 3 days in reduced media with DMSO or 1-2 nM MEK inhibitor trametinib (MEKi). Results show mean ± SEM. *, *P* < 0.05; **, *P* < 0.01; ***, *P* < 0.001, paired and unpaired Student’s *t* test.

To identify mediators of malignant cell-fibroblast crosstalk in *Smad4*-deleted KRAS^G12D^ PDAC, we evaluated bi-directional signaling in PDAC organoid/PSC co-cultures. NicheNet analysis pinpointed *Il1a* as the top malignant cell-produced mediator of malignant cell-CAF crosstalk in KPC^*Smad4*-KO^/PSC co-cultures (Fig. 5A; Supplementary Fig. 5A; Supplementary Table S7) (42). Indeed, IL-1α levels were upregulated in KPC^*Smad4*-KO^ organoids compared to KPC^*Smad4*-WT^ controls (Fig. 5B; Supplementary Fig. 5B). Corroborating previous work showing that IL-1 signaling drives the iCAF phenotype (5), IL-1α neutralization impaired the iCAF phenotype (Supplementary Fig. 5C). Thus, these data strongly suggest that increased IL-1 expression following *Smad4* loss in PDAC malignant cells enhances iCAF generation.

Of note, *Il1a* levels in organoids were further upregulated in KPC^*Smad4*-KO^/PSC co-cultures compared to monocultures, suggesting a positive feedback loop in the presence of an iCAF-rich environment (Fig. 5B). Indeed, *Il1a* also appeared to be the top mediator of both CAF-CAF crosstalk and CAF-malignant cell crosstalk in KPC^*Smad4*-KO^/PSC co-cultures (Fig. 5C-D; Supplementary Fig. 5D-E). Furthermore, *Tnf* scored as the top mediator of malignant cell-malignant cell crosstalk in KPC^*Smad4*-KO^/PSC co-cultures and monocultures (Supplementary Fig. 5F-I). In agreement with these results, *Tnf* expression was upregulated upon SMAD4 loss in PDAC cells and was not further upregulated in co-culture with PSCs (Supplementary Fig. 5J-K).

In line with these findings, IL-1 and NF-κB signaling were enriched in KPC^*Smad4*-KO^ relative to KPC^*Smad4*-WT^ monocultures and further enhanced when co-cultured with PSCs (Fig. 5E). Additionally, proliferation-associated pathways were upregulated in KPC^*Smad4*-KO^ organoids, in line with their higher proliferation rate compared to KPC^*Smad4*-WT^ controls (Fig. 5E and 2B; Supplementary Fig. 2F). JAK/STAT signaling and p-STAT3 levels were also upregulated in KPC^*Smad4*-KO^ (Fig. 5E-F). Finally, gene signatures associated with NF-κB and JAK/STAT signaling were upregulated in KPC^*Smad4*-KO^ malignant cells and whole tumors in PDAC mouse models (Fig. 5G-I; Supplementary Fig. 5L-M; Supplementary Table S8).

Our analyses show that SMAD4 loss in murine KRAS^G12D^ p53-mutant PDAC malignant cells enhances JAK/STAT activation and upregulates IL-1, promoting iCAF formation.

### SMAD4-independent TGF-β signaling pathways are upregulated in *Smad4*-deleted PDAC malignant cells

We previously found that when TGF-β signaling is reduced in PDAC CAFs, IL-1 signaling upregulates *Lif* and JAK/STAT activation, which drive iCAF formation (5). Moreover, JAK/STAT activation boosts IL-1 signaling in iCAFs, while JAK/STAT inhibition downregulates *Il1a* expression. On the contrary, addition of TGF-β to iCAFs downregulates the expression of IL1R1 and IL-1, as well as JAK/STAT activation, which are instead boosted by TGFBR1 inhibition (5). We thus investigated whether similar mechanisms occur in PDAC malignant cells upon SMAD4 loss and impairment of TGF-β signaling. Contrary to what was seen in iCAFs, treatment with the JAK inhibitor (JAKi) AZD1480 did not downregulate *Il1a* expression in KPC organoids (Supplementary Fig. 5N). Moreover, addition and neutralization of IL-1α had no impact on JAK/STAT activation in KPC organoids (Fig. 5J-K). These results suggest that IL-1 and JAK/STAT signaling are activated by different mechanisms in iCAFs and KPC^*Smad4*-KO^ malignant cells. In line with this, treatment with TGF-β upregulated the expression of *Il1a, Il1r1* and *Lif* in KPC organoids (Fig. 5L). Moreover, *Tgfb1* and phospho-SMAD2/3 (p-SMAD2/3) levels were upregulated in KPC^*Smad4*-KO^ organoids compared to KPC^*Smad4*-WT^ controls (Fig. 5J-M; Supplementary Fig. 5O). Altogether, these data indicated that TGF-β upregulation may boost SMAD4-independent TGF-β signaling in *Smad4*-deleted PDAC malignant cells, leading to *Il1a* upregulation.

A recent study showed that activation of the p38 MAPK pathway (54) can be upstream *Il1a* expression in PDAC (55). However, while phospho-p38 (p-p38) levels were higher in KPC^*Smad4*-KO^ organoids compared to KPC^*Smad4*-WT^ controls, p38 inhibition did not impair STAT3 activation or *Il1a* expression in either organoid line (Supplementary Fig. 5P-R). Thus, to identify candidate pathways activated in KPC^*Smad4*-KO^ organoids, we performed transcription factor (TF) analysis, which confirmed JAK/STAT and NF-κB signaling as main pathways active in KPC^*Smad4*-KO^ organoids (Supplementary Fig. 5S; Supplementary Table S9). This analysis also suggested increased activation of MEK/ERK MAPK signaling, and its downstream target *Jun* (56), in KPC^*Smad4*-KO^ organoids (Supplementary Fig. 5S). Since JAK/STAT and MAPK signaling have been described as SMAD4-independent TGF-β pathways (57), we hypothesized that the increased TGF-β production by KPC^*Smad4*-KO^ organoids may be responsible for their activation. In line with this, addition of TGF-β increased both Erk1/2 (i.e. p44/42) and STAT3 activation in KPC^*Smad4*-WT^ organoids (Fig. 5N). Finally, while MEK inhibition did not affect STAT3 activation, it significantly downregulated *Il1a* levels (Fig. 5O; Supplementary Fig. 5T). This suggests that increased MEK/ERK signaling in KPC^*Smad4*-KO^ organoids boosts IL-1α production.

Therefore, SMAD4 loss in murine KRAS^G12D^ p53-mutant PDAC cells leads to activation of SMAD4-independent TGF-β pathways that impact malignant signaling and the TME.

### *Smad4* loss tunes TME crosstalk and signaling dependencies in PDAC with distinct KRAS status

The *KRAS*^G12V^ mutation also occurs frequently in human PDAC (23). Therefore, we assessed whether *Smad4* loss would impact KRAS^G12V^ PDAC similarly to that observed in KRAS^G12D^ PDAC. To do this, we used PDAC organoids generated from the *Kras*^*FRT-LSL*-G12V*-FRT/+*^; *Trp53*^*LSL*-R172H^; *Pdx1-Cre*; *Rosa26-FlpO*^*ERT2*^ (hereafter, KvPC) mouse model (31) and generated isogenic *Smad4* KO (KvPC^*Smad4*-KO^) and *Smad4* WT (KvPC^*Smad4*-WT^) organoids (Supplementary Fig. S6A-D).

**Figure 6:**
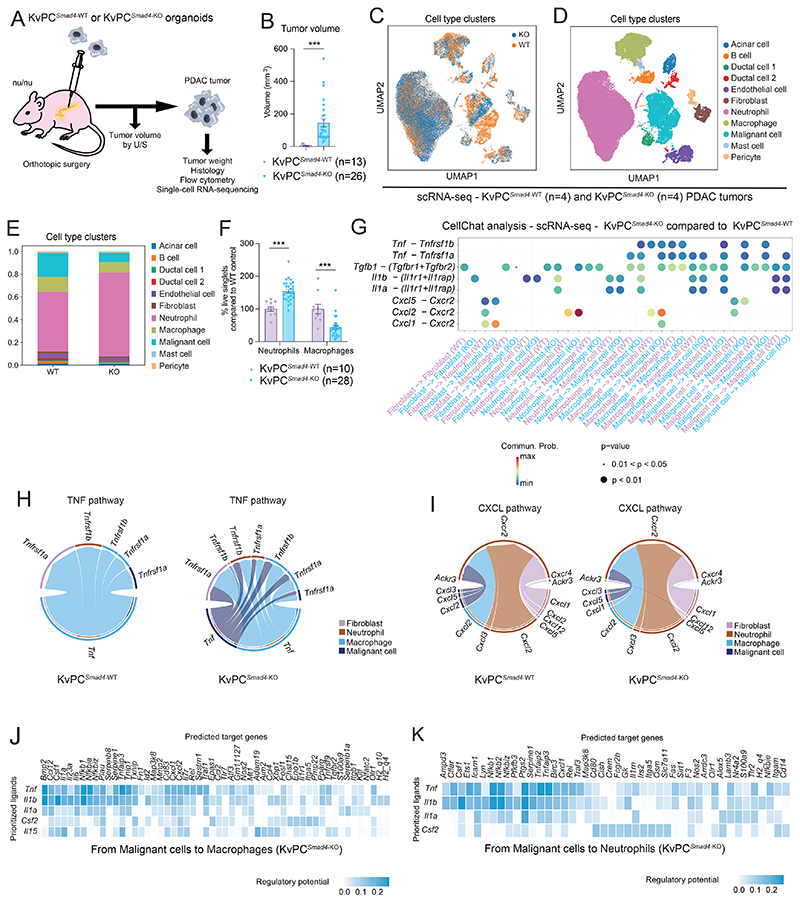
*Smad4* loss impacts the immune TME and malignant-stromal crosstalk in KvPC PDAC. **(A)** Schematic of analyses of KvPC (i.e. *Kras*^G12V^ p53-mutant) orthotopically-grafted organoid-derived PDAC models in nu/nu mice. **(B)** Volumes of tumors derived from the transplantation of *Smad4* WT or *Smad4* KO KvPC (i.e. *Kras*^G12V^ p53 mutant) PDAC organoids, measured by U/S. Results show mean ± SEM from 3 separate experiments, each with 1 WT group and 2 groups of KO pools from 2 different guides (29 days (cohort 1), 30 days (cohort 2) or 65 days (cohort 3) post-transplant). ***, *P* < 0.001, Mann-Whitney test. **(C-D)** UMAP plot of all cell types from KvPC^*Smad4*-WT^ (n=4) or KvPC^*Smad4*-KO^ (n=4) PDAC tumors analyzed by scRNA-seq, color-coded by genotype **(C)** or cell type clusters **(D). (E)** Cell type contribution in KvPC^*Smad4*-WT^ or KvPC^*Smad4*-KO^ tumors. **(F)** Flow cytometric analysis of neutrophils (CD45^+^CD11b^+^Gr1^+^) and macrophages (CD45^+^Gr1^-^CD11b^+^F4/80^+^) from live singlets in KvPC^*Smad4*-WT^ or KvPC^*Smad4*-KO^ tumors. Results show mean ± SEM from 3 separate experiments, each with 1 WT group and 2 groups of KO pools from 2 different guides. ***, *P* < 0.001, Mann-Whitney test. **(G)** Selected ligand-receptor interactions and their strength based on CellChat analysis between malignant cells, fibroblasts, macrophages, and neutrophils in KvPC^*Smad4*-KO^ compared to KvPC^*Smad4*-WT^ tumors. **(H-I)** Selected pathways with significantly different connections between malignant cells, fibroblasts, macrophages, and neutrophils in KvPC^*Smad4*-KO^ compared to KvPC^*Smad4*-WT^ tumors. **(J-K)** Ligand-target heatmaps show top selected ligands of malignant cells inferred to target genes in macrophages **(J)** and neutrophils **(K)** in KvPC^*Smad4*-KO^ PDAC, as assessed by NicheNet analysis of scRNA-seq.

**Figure 7:**
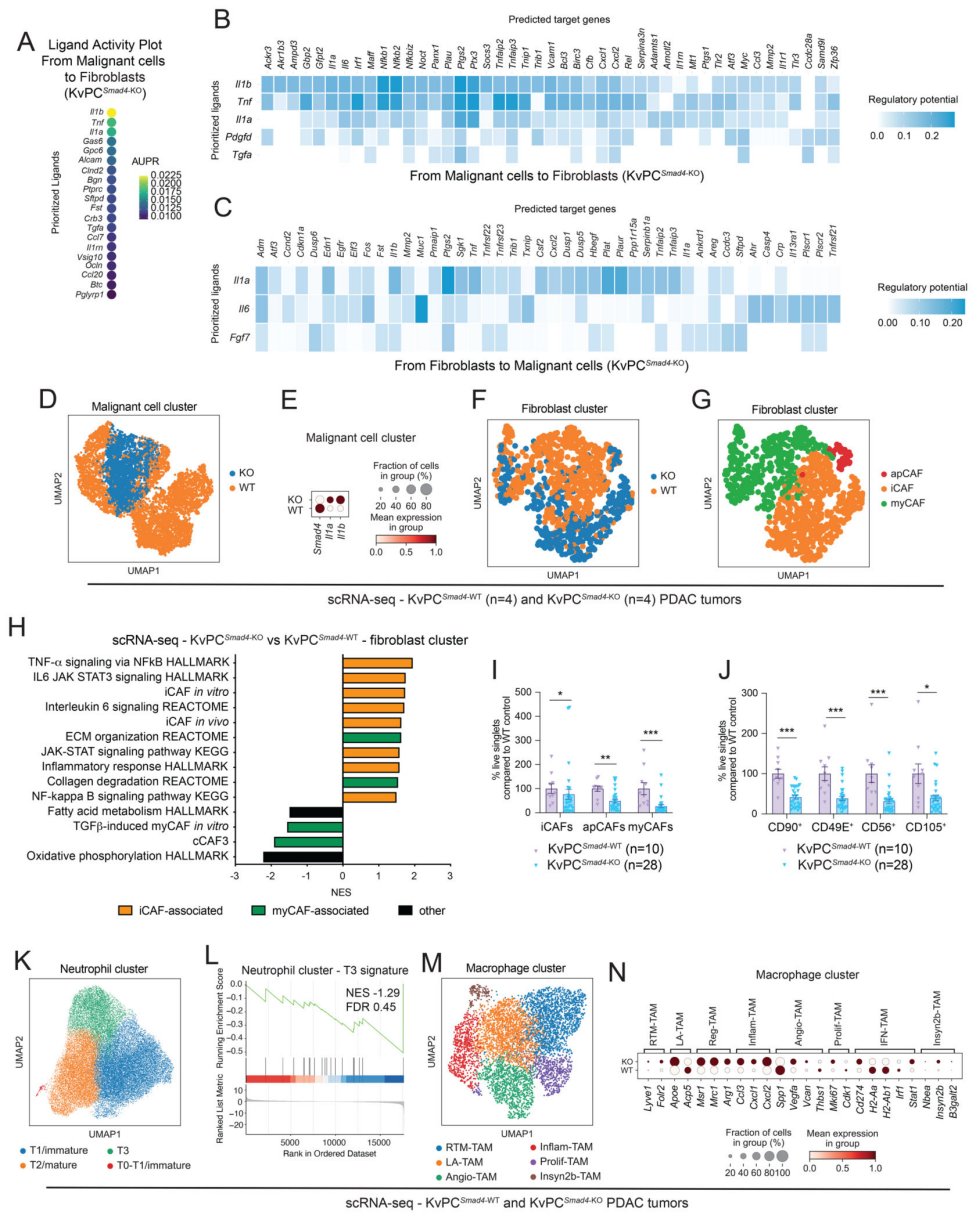
*Smad4* loss drives a fibro-inflammatory stroma in KvPC PDAC. **(A)** Ligand activity plot shows the top ligands of malignant cells inferred to regulate target genes in fibroblasts in KvPC^*Smad4*-KO^ PDAC, as assessed by NicheNet analysis of scRNA-seq. **(B-C)** Ligand-target heatmaps show top selected ligands of **(B)** malignant cells inferred to regulate target genes in fibroblasts and **(C)** fibroblasts inferred to regulate target genes in malignant cells in KPC^*Smad4*-KO^ PDAC, as assessed by NicheNet analysis. **(D)** UMAP plot of malignant cells from KvPC^*Smad4*-WT^ (n=4) or KvPC^*Smad4*-KO^ (n=4) tumors analyzed by scRNA-seq, color-coded by genotype. **(E)** Dot plot visualization of the scaled average expression of *Smad4, Il1a* and *Il1b* in malignant cells of KvPC^*Smad4*-WT^ or KvPC^*Smad4*-KO^ tumors, as analyzed by scRNA-seq. Color intensity represents expression level and dot size represents the percentage of expressing cells. **(F-G)** UMAP plots of CAFs from KvPC^*Smad4*-WT^ or KvPC^*Smad4*-KO^ tumors analyzed by scRNA-seq, color-coded by genotype **(F)** or sub-cluster **(G). (H)** Selected significantly upregulated (i.e. NES > 1.50 and FDR < 0.25; apart from the NF-kappa B signaling pathway with NES = 1.49) and downregulated (i.e. NES < -1.50 and FDR < 0.25; apart from the fatty acid metabolism with NES = -1.48) pathways identified by GSEA of CAFs from KvPC^*Smad4*-KO^ compared to KvPC^*Smad4*-WT^ tumors, as assessed by pseudobulk analysis from scRNA-seq. The *in vivo* iCAF signature is from Elyada et al (3). The *in vitro* iCAF signature is from Öhlund et al (4). The TGF-β-induced myCAF *in vitro* signature is from Mucciolo and Araos Henríquez et al (11). The cCAF3 signature is from McAndrews et al (8). **(I-J)** Flow cytometric analyses of **(I)** myCAFs (Ly6C^-^MHCII^-^ CAFs), iCAFs (Ly6C^+^MHCII^-^ CAFs) and apCAFs (Ly6C^-^MHCII^+^ CAFs), and **(J)** CD90^+^, CD49E^+^, CD56^+^ and CD105^+^ CAFs from live singlets in KvPC^*Smad4*-WT^ or KvPC^*Smad4*-KO^ tumors. Results show mean ± SEM from 3 separate experiments, each with 1 WT group and 2 groups of KO pools from 2 different guides. *, *P* < 0.05; **, *P* < 0.01; ***, *P* < 0.001, Mann-Whitney test. **(K)** UMAP plot of neutrophils from KvPC^*Smad4*-WT^ or KvPC^*Smad4*-KO^ PDAC analyzed by scRNA-seq. Different sub-clusters are color-coded. **(L)** GSEA of T3 neutrophil signature in neutrophils from KvPC^*Smad4*-KO^ compared to KvPC^*Smad4*-WT^ PDAC. The signature from Ng et al (13) is not significantly altered. **(M)** UMAP plot of macrophages from KvPC^*Smad4*-WT^ or KvPC^*Smad4*-KO^ tumors analyzed by scRNA-seq. Different sub-clusters are color-coded. **(N)** Dot plot visualization of the scaled average expression of macrophage markers in macrophages from KvPC^*Smad4*-WT^ or KvPC^*Smad4*-KO^ tumors analyzed by scRNA-seq. Color intensity represents expression level and dot size represents the percentage of expressing cells.

Allografted KvPC^*Smad4*-KO^ organoids formed tumors that grew significantly faster (Fig. 6A-B; Supplementary Fig. 6E). KvPC^*Smad4*-KO^ PDAC also contained more neutrophils and fewer macrophages than KvPC^*Smad4*-WT^ tumors of similar size (Fig. 6C-F; Supplementary Fig. 6F-N; Supplementary Table S10). Furthermore, certain malignant cell-stromal interactions were similarly altered in both KRAS mutant-driven models upon SMAD4 loss. These included the potential role of malignant cell-produced TNF in shaping macrophage and neutrophil composition and of macrophage-produced CXCL1 and CXCL2 in recruiting neutrophils in KvPC^*Smad4*-KO^ PDAC (Fig. 6G-K; Supplementary Fig. 6O-S; Supplementary Table S11). IL-1 signaling mediated malignant cell-fibroblast crosstalk also in KvPC^*Smad4*-KO^ PDAC (Fig. 7A-C; Supplementary Fig. 7A-H). Accordingly, *Il1a* and *Il1b* expression were upregulated in KvPC^*Smad4*-KO^ malignant cells (Fig. 7D-E). Furthermore, scRNA-seq profiles of KvPC^*Smad4*-KO^ PDAC-derived CAFs revealed a decrease in myCAF-associated pathways and an increase in iCAF-associated pathways (Fig. 7F-H; Supplementary Fig. 7I). Additionally, flow cytometry analysis confirmed loss of myCAFs, apCAFs and CD90^+^, CD49E^+^, CD56^+^ and CD105^+^ myCAF populations, as well as significant downregulation of the myCAF/iCAF ratio, in KvPC^*Smad4*-KO^ PDAC tumors compared to WT controls (Fig. 7I-J; Supplementary Fig. 7J-K).

**Figure 8:**
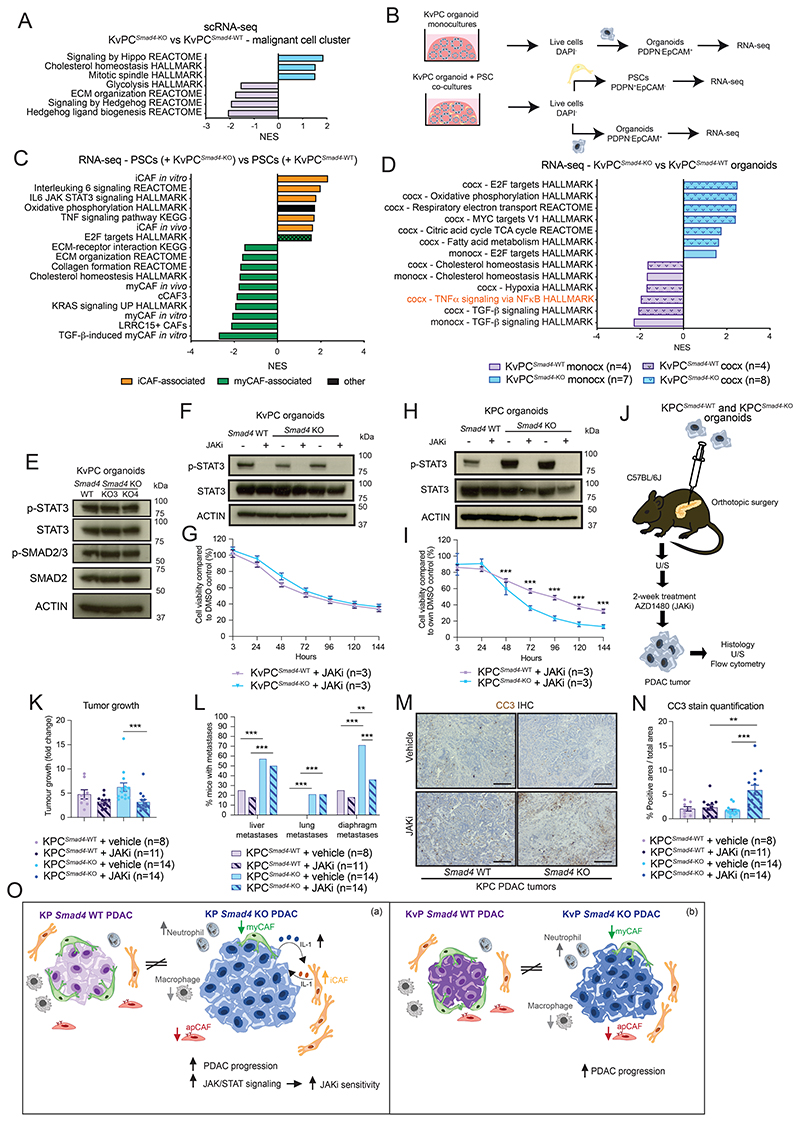
*Smad4* loss tunes signaling dependencies in PDAC with distinct KRAS status. **(A)** Selected significantly upregulated or downregulated pathways identified by GSEA of malignant cells from KvPC^*Smad4*-KO^ PDAC (n=4) compared to malignant cells from KvPC^*Smad4*-WT^ tumors (n=4), as assessed by pseudobulk analysis of the scRNA-seq dataset. **(B)** Schematic of flow-sorting strategy of PSCs and KvPC^*Smad4*-WT^ or KvPC^*Smad4*-KO^ PDAC organoids from monocultures or co-cultures for RNA-seq analysis. **(C)** Selected significantly upregulated and downregulated pathways identified by GSEA of PSCs cultured with KvPC^*Smad4*-KO^ organoids (n=8) compared to PSCs cultured with KvPC^*Smad4*-WT^ organoids (n=4). The *in vivo* iCAF and myCAF signatures are from Elyada et al (3). The *in vitro* iCAF and myCAF signatures are from Öhlund et al (4). The TGF-β-induced myCAF *in vitro* signature is from Mucciolo and Araos Henríquez et al (11). The LRRC15^+^ CAF and cCAF3 signatures are from Dominguez et al (7) and McAndrews et al (8), respectively. **(D)** Selected pathways found significantly enriched or depleted by GSEA in KvPC^*Smad4*-KO^ malignant cells compared to KvPC^*Smad4*-WT^ malignant cells flow-sorted from monocultures or co-cultures with PSCs. The smooth pattern indicates the monocultures. Inflammatory pathways are highlighted in orange. **(E)** Western blot analysis of p-STAT3, STAT3, p-SMAD2/3 and SMAD2 in KvPC^*Smad4*-WT^ or KvPC^*Smad4*-KO^ organoids cultured in reduced media for 2 days. ACTIN, loading control. **(F)** Western blot analysis of p-STAT3 and STAT3 in KvPC^*Smad4*-WT^ or KvPC^*Smad4*-KO^ organoids cultured for 2 days in reduced media with or without 8 μM of the JAK inhibitor (JAKi, AZD1480). ACTIN, loading control. **(G)** Proliferation curves of KvPC^*Smad4*-WT^ and KvPC^*Smad4*-KO^ organoids cultured for 144 hours in reduced media with or without 8 μM JAKi. Data were normalized to the first measurement (3 hours post-plating) and to the DMSO control. Results show mean ± SEM of n=3 biological replicates (with n=6 technical replicates each). No statistical difference was found by Mann-Whitney test. **(H)** Western blot analysis of p-STAT3 and STAT3 in KPC^*Smad4*-WT^ or KPC^*Smad4*-KO^ organoids cultured for 2 days in reduced media with or without 8 μM of the JAKi. ACTIN, loading control. **(I)** Proliferation curves of KPC^*Smad4*-WT^ and KPC^*Smad4*-KO^ organoids cultured for 144 hours in reduced media with or without 8 μM JAKi. Data were normalized to the first measurement (3 hours post-plating) and to the DMSO control. Results show mean ± SEM of n=3 biological replicates (with n=6 technical replicates each). ***, *P* < 0.001, Mann-Whitney test. **(J)** Schematic of experimental design and downstream analyses of a 2-week JAKi treatment of KPC^*Smad4*-WT^ and KPC^*Smad4*-KO^ organoid-derived PDAC models in C57BL/6J mice. **(K)** Tumor growth (i.e. ratio of tumor volume at day 14 and tumor volume at day -1), measured by U/S, of 2-week vehicle- and JAKi-treated KPC^*Smad4*-WT^ or KPC^*Smad4*-KO^ PDAC tumors. Results show mean ± SEM from 3 separate experiments. ***, *P* < 0.001, Mann-Whitney test. **(L)** Percentage of KPC^*Smad4*-WT^ and KPC^*Smad4*-KO^ tumor-bearing mice with metastases in the liver, lungs and diaphragm following 2 weeks of treatment with vehicle or JAKi. Results show data from 3 experiments. **, *P* < 0.01, ***, *P* < 0.001, chi-square test. **(M)** Representative cleaved caspase 3 (CC3) immunohistochemistry (IHC) stains in 2-week vehicle- or JAKi- treated KPC^*Smad4*-WT^ and KPC^*Smad4*-KO^ PDAC tumors. Scale bars, 50 μm. **(N)** Quantification of CC3 stain in 2-week vehicle- or JAKi-treated KPC^*Smad4*-WT^ and KPC^*Smad4*-KO^ PDAC tumors. Results show mean ± SEM from 3 separate experiments. **, *P* < 0.01; ***, *P* < 0.001, Mann-Whitney test. **(O)** Model illustrating how *Smad4* loss differently shapes murine KRAS^G12D^ (a) or KRAS^G12V^ (b) p53 mutant PDAC tumors, including their progression, tumor microenvironment and therapeutic sensitivity.

Despite these similarities with KPC tumors, abundance of iCAFs, total CAFs and tumor-promoting CD90^-^ myCAFs was reduced in KvPC^*Smad4*-KO^ PDAC (Fig. 7I; Supplementary Fig. 7L-M). Moreover, the T3 neutrophil signature was not significantly upregulated in KvPC^*Smad4*-KO^ PDAC (Fig. 7K-L; Supplementary Fig. 7N-O). Furthermore, while the expression of genes associated with Inflam-TAMs was upregulated in KvPC^*Smad4*-KO^ PDAC, as observed in KPC^*Smad4*-KO^ tumors, genes associated with Prolif-TAMs were not clearly downregulated, and markers of IFN-TAMs or LA-TAMs were not clearly increased in KvPC^*Smad4*-KO^ PDAC compared to WT controls (Fig. 7M-N; Supplementary Fig. 7P-Q). Thus, SMAD4 loss has different effects on the stroma composition of murine PDAC tumors with distinct KRAS mutations. Moreover, SMAD4 loss was not sufficient to drive an increase in KvPC organoid proliferation *in vitro*, suggesting that the increase in tumor growth *in vivo* is mediated by changes in the TME and their crosstalk with malignant cells (Supplementary Fig. 7R). Finally, most strikingly, the JAK/STAT and NF-κB pathways were not upregulated in KvPC^*Smad4*-KO^ malignant cells *in vivo* (Fig. 8A).

To further explore how SMAD4 loss directly impacts KvPC malignant cells, we established co-cultures of PSCs and KvPC organoids and analyzed both flow-sorted populations by RNA-seq (Fig. 8B; Supplementary Fig. 7S; Supplementary Tables S12-S13). Validating our *in vivo* observations, iCAF-associated pathways were upregulated, and myCAF-associated pathways were downregulated, when PSCs were co-cultured with KvPC^*Smad4*-KO^ organoids, albeit with some differences compared to KPC^*Smad4*-KO^ co-cultures (Fig. 8C; Supplementary Fig. 8A). Of note, as observed *in vivo*, JAK/STAT signaling and p-STAT3 levels were not upregulated in KvPC malignant cells following SMAD4 loss (Fig. 8D-E). Additionally, neither *Tgfb1* nor p-SMAD2/3 levels were upregulated in KvPC^*Smad4*-KO^ organoids compared to KvPC^*Smad4*-WT^ controls (Fig. 8E; Supplementary Fig. 8B).

Since JAK/STAT signaling was upregulated upon *Smad4* deletion in KPC, but not KvPC, malignant cells, we evaluated whether inhibition of this pathway led to an increased therapeutic sensitivity only in KPC^*Smad4*-KO^ PDAC. No significant difference was observed in the proliferation of KvPC^*Smad4*-KO^ organoids compared to KvPC^*Smad4*-WT^ controls when exposed to the JAKi (5,58) (Fig. 8F-G). In contrast, KPC^*Smad4*-KO^ organoids were more sensitive to JAK inhibition than KPC^*Smad4*-WT^ controls (Fig. 8H-I). To test if this difference in JAK signaling dependency also exists *in vivo*, we treated mice harboring KPC^*Smad4*-KO^ or KPC^*Smad4*-WT^ PDACs for two weeks with the JAKi (Fig. 8J; Supplementary Fig. 8C-E). Remarkably, JAK inhibition impaired tumor growth and diaphragm metastases of KPC^*Smad4*-KO^, but not KPC^*Smad4*-WT^, models (Fig. 8K-L; Supplementary Fig. 8F-G). Moreover, levels of the apoptotic marker cleaved caspase 3 (CC3) were upregulated in JAKi-treated KPC^*Smad*-KO^ tumors (Fig. 8M-N; Supplementary Fig. 8H-K). This suggests a selective increase in cell death upon JAK inhibition in KRAS^G12D^ p53-mutant PDAC with SMAD4 loss. In line with this, live singlets were reduced in JAKi-treated KPC^*Smad*-KO^ tumors (Supplementary Fig. 8L). Moreover, although the myCAF/iCAF ratio was not significantly altered, collagen deposition was increased in KPC^*Smad4*-KO^ PDAC following JAK/STAT inhibition, suggesting a change in CAF composition towards a more myofibroblastic phenotype (Supplementary Fig. 8M-P). Additionally, in JAKi-treated KPC^*Smad4*-KO^ PDAC, macrophages, apCAFs and FOXP3^+^ T regulatory T cells were reduced, and non-exhausted total T cells and CD8^+^ T cells were increased (59), which may contribute to impairing tumor growth (Supplementary Fig. 8N and 8Q-U).

Together, these analyses suggest that SMAD4 loss differently impacts malignant-stromal crosstalk and therapeutic sensitivities in murine PDAC with distinct KRAS status.

## Discussion

Cancers are rarely driven by single mutations. Distinct combinations of mutations can have both malignant cell intrinsic and extrinsic effects. Understanding how different combinations of mutations cooperate to drive the malignant phenotype is key if we aim to develop more effective cancer therapies. Here, we show how SMAD4 loss in PDAC malignant cells shapes tumor biology differently in the presence of two distinct KRAS mutations (Fig. 8O). While loss of SMAD4 generated a more fibro-inflammatory TME in both KPC and KvPC murine PDAC, it also led to distinct differences depending on the KRAS status. In addition, SMAD4 loss was associated with an increase in JAK/STAT dependency in KPC, but not KvPC, PDAC compared to *Smad4* WT controls. Impaired tumor growth of JAK inhibitor-treated KPC^*Smad4*-KO^ tumors was associated with an increase in apoptosis, as well as CD8^+^ T cell abundance. Considering recent studies demonstrating enhanced efficacy of JAK/STAT inhibition with immunotherapy in cancer (60,61), *Smad4*-deleted PDAC may show increased sensitivity to this combination regimen. SMAD4 and KRAS mutation-specific differences in JAK/STAT dependency may start to explain why JAK inhibitors showed promise in pre-clinical studies but failed to display benefit when used to treat patients with genetically heterogeneous PDAC (5,62–64). Although these clinical trials were done in the presence of chemotherapy, and such combinations could be explored in the future, our data suggest that better understanding of how PDAC genetics impact malignant-stromal crosstalk is needed if we are to deploy more effective targeted therapies in the clinic. Of note, MAPK signaling was also upregulated in KPC^*Smad4*-KO^ organoids, and *Smad4*-deficient PDAC cells have been shown to be more susceptible than *Smad4* WT cells to MEK inhibition (65).

Our study also highlights the power of comparing isogenic murine organoid-derived models to begin to deconvolute the complexity of human PDAC and pinpoint the impact of combinations of mutations on malignant cells and TME. Further modeling of the genetic complexity observed in patients will enable to better understand its impact on bi-directional malignant-stromal crosstalk, PDAC progression and therapeutic vulnerabilities. Due to the heterogeneous nature of the TME, scRNA-seq studies coupled with genetic information will be key in future efforts to more effectively target the biology of distinct groups of PDAC.

Upregulation of STAT3 signaling in the epithelial compartment of KPC GEMMs was also observed following TGFBR2 loss and was associated with increased tissue tension and collagen fiber changes (28,52,66). Here, we found increased STAT3 activation in both malignant cells and the iCAF-rich microenvironment associated with KPC^*Smad4*-KO^, but not KvPC^*Smad4*-KO^, PDAC compared to *Smad4* WT tumors. Furthermore, contrary to TGFBR2 loss (28), SMAD4 deletion in KPC and KvPC PDAC drove primary tumor growth *in vivo*. Accordingly, among mutations associated with impaired TGF-β signaling, only SMAD4 loss was associated with worse survival of PDAC patients (52). Of note, our pre-clinical study suggested that growth kinetics of established KPC^*Smad4*-KO^ and KPC^*Smad4*-WT^ tumors is comparable.

Evidence suggests that inflammatory CAFs play tumor-promoting and immunosuppressive roles in cancer (2,5,67,68). Therefore, since iCAFs increase in *Smad4*-deleted PDAC, it is tempting to speculate that they may be involved in the aggressive phenotype of these tumors. However, this remains to be assessed. Similarly, whether the decrease in apCAFs or increase in metastasis-promoting CD90^-^ EGFR-activated myCAFs (11) participate in driving the aggressive phenotype of KPC^*Smad4*-KO^ PDAC remains to be determined. Fibroblast-specific GEMMs will be required to address these questions. Finally, the reduction in NK cells, which typically play anti-tumor roles (69,70), as well as changes in macrophage and neutrophil phenotype and abundance may also contribute to the faster progression of *Smad4*-deleted PDAC. Indeed, macrophages contribute to PDAC development and therapies targeting neutrophils in PDAC mouse models hindered tumor progression (71,72).

As KRAS mutant-specific and pan-RAS inhibitors are being developed (73–75), understanding differences between KRAS mutations, and their associated TMEs, could be key to design effective combinatorial strategies. While our analyses revealed similar effects caused by SMAD4 loss in the context of KPC and KvPC tumors, they also highlighted important differences in malignant cell features and stroma composition. Whether these are at least partially dependent on intrinsic differences of distinct KRAS mutations remains to be determined. Similarly, further investigation will be pivotal to determine the roles of other mutations in shaping the stroma and therapy response of PDAC.

## Supplementary Material

Supp Figures and legends

Table 1

Table 2

Table 3

Table 4

Table 5

Table 6

Table 7

Table 8

Table 9

Table 10

Table 11

Table 12

Table 13

## Data Availability

For RNA-seq and scRNA-seq datasets, the data generated in this study are publicly available at the GEO under the accession numbers GSE263080, GSE263081, GSE262879, GSE262878 and GSE274684. For ultrasound-based tumor volume, western blot, qPCR, organoid proliferation assay, flow cytometry, histology and immunochemistry data, the raw data are available on request from the corresponding author. No publicly available data was reused. This paper does not report original code.

## Statement of Significance

SMAD4 loss differentially impacts malignant-stromal cell signaling and treatment sensitivity of pancreatic tumors driven by KRAS^G12D^ or KRAS^*G12V*^, highlighting the importance of understanding genotype-phenotype relationships for precision therapy.

## References

[1] Siegel RL, Giaquinto AN, Jemal A (2024). Cancer statistics, 2024. CA: A Cancer Journal for Clinicians.

[2] Biffi G, Tuveson DA (2021). Diversity and Biology of Cancer-Associated Fibroblasts. Physiol Rev.

[3] Elyada E, Bolisetty M, Laise P, Flynn WF, Courtois ET, Burkhart RA (2019). Cross-species single-cell analysis of pancreatic ductal adenocarcinoma reveals antigen-presenting cancer-associated fibroblasts. Cancer Discov.

[4] Ohlund D, Handly-Santana A, Biffi G, Elyada E, Almeida AS, Ponz-Sarvise M (2017). Distinct populations of inflammatory fibroblasts and myofibroblasts in pancreatic cancer. J Exp Med.

[5] Biffi G, Oni TE, Spielman B, Hao Y, Elyada E, Park Y (2019). IL1-Induced JAK/STAT Signaling Is Antagonized by TGFbeta to Shape CAF Heterogeneity in Pancreatic Ductal Adenocarcinoma. Cancer Discov.

[6] Hutton C, Heider F, Blanco-Gomez A, Banyard A, Kononov A, Zhang X (2021). Single-cell analysis defines a pancreatic fibroblast lineage that supports anti-tumor immunity. Cancer Cell.

[7] Dominguez CX, Muller S, Keerthivasan S, Koeppen H, Hung J, Gierke S (2020). Single-Cell RNA Sequencing Reveals Stromal Evolution into LRRC15(+) Myofibroblasts as a Determinant of Patient Response to Cancer Immunotherapy. Cancer Discov.

[8] McAndrews KM, Chen Y, Darpolor JK, Zheng X, Yang S, Carstens JL (2022). Identification of Functional Heterogeneity of Carcinoma-Associated Fibroblasts with Distinct IL6-Mediated Therapy Resistance in Pancreatic Cancer. Cancer Discov.

[9] Helms EJ, Berry MW, Chaw RC, DuFort CC, Sun D, Onate MK (2022). Mesenchymal Lineage Heterogeneity Underlies Nonredundant Functions of Pancreatic Cancer-Associated Fibroblasts. Cancer Discov.

[10] Huang H, Wang Z, Zhang Y, Pradhan RN, Ganguly D, Chandra R (2022). Mesothelial cell-derived antigen-presenting cancer-associated fibroblasts induce expansion of regulatory T cells in pancreatic cancer. Cancer Cell.

[11] Mucciolo G, Araos Henríquez J, Jihad M, Pinto Teles S, Manansala JS, Li W (2023). EGFR-activated myofibroblasts promote metastasis of pancreatic cancer. Cancer Cell.

[12] Ma R-Y, Black A, Qian B-Z (2022). Macrophage diversity in cancer revisited in the era of single-cell omics. Trends Immunol.

[13] Ng MSF, Kwok I, Tan L, Shi C, Cerezo-Wallis D, Tan Y (2024). Deterministic reprogramming of neutrophils within tumors. Science.

[14] Lloyd EG, Henríquez JA, Biffi G (2024). Modelling the micro- and macro- environment of pancreatic cancer: from patients to pre-clinical models and back. Disease Models & Mechanisms.

[15] Collisson EA, Sadanandam A, Olson P, Gibb WJ, Truitt M, Gu S (2011). Subtypes of pancreatic ductal adenocarcinoma and their differing responses to therapy. Nature Medicine.

[16] Moffitt RA, Marayati R, Flate EL, Volmar KE, Loeza SGH, Hoadley KA (2015). Virtual microdissection identifies distinct tumor- and stroma-specific subtypes of pancreatic ductal adenocarcinoma. Nature Genetics.

[17] Bailey P, Chang DK, Nones K, Johns AL, Patch AM, Gingras MC (2016). Genomic analyses identify molecular subtypes of pancreatic cancer. Nature.

[18] Somerville TD, Biffi G, Daßler-Plenker J, Hur SK, He X-Y, Vance KE, Sawyers CL, Ojala PM, Oliver T (2020). Squamous trans-differentiation of pancreatic cancer cells promotes stromal inflammation. eLife.

[19] Raghavan S, Winter PS, Navia AW, Williams HL, DenAdel A, Lowder KE (2021). Microenvironment drives cell state, plasticity, and drug response in pancreatic cancer. Cell.

[20] Chan-Seng-Yue M, Kim JC, Wilson GW, Ng K, Figueroa EF, O’Kane GM (2020). Transcription phenotypes of pancreatic cancer are driven by genomic events during tumor evolution. Nat Genet.

[21] Williams HL, Dias Costa A, Zhang J, Raghavan S, Winter PS, Kapner KS (2022). Spatially-resolved single-cell assessment of pancreatic cancer expression subtypes reveals co-expressor phenotypes and extensive intra-tumoral heterogeneity. Cancer Research.

[22] Aguirre AJ, Nowak JA, Camarda ND, Moffitt RA, Ghazani AA, Hazar-Rethinam M (2018). Real-time genomic characterization of advanced pancreatic cancer to enable precision medicine. Cancer Discovery.

[23] Raphael BJ, Hruban RH, Aguirre AJ, Moffitt RA, Yeh JJ, Stewart C (2017). Integrated Genomic Characterization of Pancreatic Ductal Adenocarcinoma. Cancer Cell.

[24] Hingorani SR, Wang L, Multani AS, Combs C, Deramaudt TB, Hruban RH (2005). Trp53R172H and KrasG12D cooperate to promote chromosomal instability and widely metastatic pancreatic ductal adenocarcinoma in mice. Cancer Cell.

[25] Maddalena M, Mallel G, Nataraj NB, Shreberk-Shaked M, Hassin O, Mukherjee S (2021). TP53 missense mutations in PDAC are associated with enhanced fibrosis and an immunosuppressive microenvironment. Proc Natl Acad Sci U S A.

[26] Shaashua L, Ben-Shmuel A, Pevsner-Fischer M, Friedman G, Levi-Galibov O, Nandakumar S (2022). BRCA mutational status shapes the stromal microenvironment of pancreatic cancer linking clusterin expression in cancer associated fibroblasts with HSF1 signaling. Nat Commun.

[27] Vennin C, Mélénec P, Rouet R, Nobis M, Cazet AS, Murphy KJ (2019). CAF hierarchy driven by pancreatic cancer cell p53-status creates a pro-metastatic and chemoresistant environment via perlecan. Nat Commun.

[28] Laklai H, Miroshnikova YA, Pickup MW, Collisson EA, Kim GE, Barrett AS (2016). Genotype tunes pancreatic ductal adenocarcinoma tissue tension to induce matricellular fibrosis and tumor progression. Nature Medicine.

[29] Iacobuzio-Donahue CA, Fu B, Yachida S, Luo M, Abe H, Henderson CM (2009). DPC4 Gene Status of the Primary Carcinoma Correlates With Patterns of Failure in Patients With Pancreatic Cancer. JCO.

[30] Malinova A, Schreyer D, Fiorini E, Pasini D, Bevere M, D’Agosto S (2023). ecDNA amplification of MYC drives intratumor copy-number heterogeneity and adaptation to stress in PDAC. BioRxiv.

[31] Humpton TJ, Alagesan B, DeNicola GM, Lu D, Yordanov GN, Leonhardt CS (2019). Oncogenic KRAS Induces NIX-Mediated Mitophagy to Promote Pancreatic Cancer. Cancer Discovery.

[32] Oni TE, Biffi G, Baker LA, Hao Y, Tonelli C, Somerville TDD (2020). SOAT1 promotes mevalonate pathway dependency in pancreatic cancer. J Exp Med.

[33] Filippini D, Agosto SD, Delfino P, Simbolo M, Piro G, Rusev B (2019). Immunoevolution of mouse pancreatic organoid isografts from preinvasive to metastatic disease. Sci Rep.

[34] Vassilev LT, Vu BT, Graves B, Carvajal D, Podlaski F, Filipovic Z (2004). In Vivo Activation of the p53 Pathway by Small-Molecule Antagonists of MDM2. Science.

[35] Tiriac H, Belleau P, Engle DD, Plenker D, Deschênes A, Somerville TDD (2018). Organoid profiling identifies common responders to chemotherapy in pancreatic cancer. Cancer Discov.

[36] Boj SF, Hwang C-I, Baker LA, Chio IIC, Engle DD, Corbo V (2015). Organoid models of human and mouse ductal pancreatic cancer. Cell.

[37] Patro R, Duggal G, Love MI, Irizarry RA, Kingsford C (2017). Salmon provides fast and bias-aware quantification of transcript expression. Nat Methods.

[38] Soneson C, Love MI, Robinson MD (2015). Differential analyses for RNA-seq: transcript-level estimates improve gene-level inferences. F1000Res.

[39] Love MI, Huber W, Anders S (2014). Moderated estimation of fold change and dispersion for RNA-seq data with DESeq2. Genome Biology.

[40] Zhu A, Ibrahim JG, Love MI (2019). Heavy-tailed prior distributions for sequence count data: removing the noise and preserving large differences. Bioinformatics.

[41] Subramanian A, Tamayo P, Mootha VK, Mukherjee S, Ebert BL, Gillette MA (2005). Gene set enrichment analysis: A knowledge-based approach for interpreting genome-wide expression profiles. Proceedings of the National Academy of Sciences.

[42] Browaeys R, Saelens W, Saeys Y (2020). NicheNet: modeling intercellular communication by linking ligands to target genes. Nat Methods.

[43] Müller-Dott S, Tsirvouli E, Vazquez M, Ramirez Flores RO, Badia-I-Mompel P, Fallegger R (2023). Expanding the coverage of regulons from high-confidence prior knowledge for accurate estimation of transcription factor activities. Nucleic Acids Res.

[44] Badia-I-Mompel P, Vélez Santiago J, Braunger J, Geiss C, Dimitrov D, Müller-Dott S (2022). decoupleR: ensemble of computational methods to infer biological activities from omics data. Bioinform Adv.

[45] Zheng GXY, Terry JM, Belgrader P, Ryvkin P, Bent ZW, Wilson R (2017). Massively parallel digital transcriptional profiling of single cells. Nat Commun.

[46] Bernstein NJ, Fong NL, Lam I, Roy MA, Hendrickson DG, Kelley DR (2020). Solo: Doublet Identification in Single-Cell RNA-Seq via Semi-Supervised Deep Learning. Cell Syst.

[47] Gayoso A, Lopez R, Xing G, Boyeau P, Valiollah Pour Amiri V, Hong J (2022). A Python library for probabilistic analysis of single-cell omics data. Nat Biotechnol.

[48] Wolf FA, Angerer P, Theis FJ (2018). SCANPY: large-scale single-cell gene expression data analysis. Genome Biology.

[49] Dann E, Henderson NC, Teichmann SA, Morgan MD, Marioni JC (2022). Differential abundance testing on single-cell data using k-nearest neighbor graphs. Nat Biotechnol.

[50] Jin S, Guerrero-Juarez CF, Zhang L, Chang I, Ramos R, Kuan C-H (2021). Inference and analysis of cell-cell communication using CellChat. Nat Commun.

[51] Finak G, McDavid A, Yajima M, Deng J, Gersuk V, Shalek AK (2015). MAST: a flexible statistical framework for assessing transcriptional changes and characterizing heterogeneity in single-cell RNA sequencing data. Genome Biology.

[52] Blackford A, Serrano OK, Wolfgang CL, Parmigiani G, Jones S, Zhang X (2009). SMAD4 gene mutations are associated with poor prognosis in pancreatic cancer. Clin Cancer Res.

[53] Schwoerer S, Cimino FV, Ros M, Tsanov KM, Ng C, Lowe SW (2023). Hypoxia potentiates the inflammatory fibroblast phenotype promoted by pancreatic cancer cell-derived cytokines. Cancer Research.

[54] Luo K (2017). Signaling Cross Talk between TGF-β/Smad and Other Signaling Pathways. Cold Spring Harb Perspect Biol.

[55] Singh SP, Dosch AR, Mehra S, De Castro Silva I, Bianchi A, Garrido VT (2024). Tumor Cell-Intrinsic p38 MAPK Signaling Promotes IL1α-Mediated Stromal Inflammation and Therapeutic Resistance in Pancreatic Cancer. Cancer Res.

[56] Whitmarsh AJ, Davis RJ (1996). Transcription factor AP-1 regulation by mitogen-activated protein kinase signal transduction pathways. J Mol Med (Berl).

[57] Tang L-Y, Heller M, Meng Z, Yu L-R, Tang Y, Zhou M (2017). Transforming Growth Factor-β (TGF-β) Directly Activates the JAK1-STAT3 Axis to Induce Hepatic Fibrosis in Coordination with the SMAD Pathway. J Biol Chem.

[58] Hedvat M, Huszar D, Herrmann A, Gozgit JM, Schroeder A, Sheehy A (2009). The JAK2 Inhibitor, AZD1480, Potently Blocks Stat3 Signaling and Oncogenesis in Solid Tumors. Cancer Cell.

[59] Steele NG, Carpenter ES, Kemp SB, Sirihorachai VR, The S, Delrosario L (2020). Multimodal Mapping of the Tumor and Peripheral Blood Immune Landscape in Human Pancreatic Cancer. Nat Cancer.

[60] Zak J, Pratumchai I, Marro BS, Marquardt KL, Zavareh RB, Lairson LL (2024). JAK inhibition enhances checkpoint blockade immunotherapy in patients with Hodgkin lymphoma. Science.

[61] Mathew D, Marmarelis ME, Foley C, Bauml JM, Ye D, Ghinnagow R (2024). Combined JAK inhibition and PD-1 immunotherapy for non-small cell lung cancer patients. Science.

[62] Hurwitz H, Van Cutsem E, Bendell J, Hidalgo M, Li C-P, Salvo MG (2018). Ruxolitinib + capecitabine in advanced/metastatic pancreatic cancer after disease progression/intolerance to first-line therapy: JANUS 1 and 2 randomized phase III studies. Invest New Drugs.

[63] Wörmann SM, Song L, Ai J, Diakopoulos KN, Kurkowski MU, Görgülü K (2016). Loss of P53 Function Activates JAK2-STAT3 Signaling to Promote Pancreatic Tumor Growth, Stroma Modification, and Gemcitabine Resistance in Mice and Is Associated With Patient Survival. Gastroenterology.

[64] Nagathihalli NS, Castellanos JA, Shi C, Beesetty Y, Reyzer ML, Caprioli R (2015). Signal Transducer and Activator of Transcription 3, Mediated Remodeling of the Tumor Microenvironment Results in Enhanced Tumor Drug Delivery in a Mouse Model of Pancreatic Cancer. Gastroenterology.

[65] Hasselluhn MC, Schlösser D, Versemann L, Schmidt GE, Ulisse M, Oschwald J (2024). An NFATc1/SMAD3/cJUN Complex Restricted to SMAD4-Deficient Pancreatic Cancer Guides Rational Therapies. Gastroenterology.

[66] Yachida S, White CM, Naito Y, Zhong Y, Brosnan JA, Macgregor-Das AM (2012). Clinical significance of the genetic landscape of pancreatic cancer and implications for identification of potential long-term survivors. Clin Cancer Res.

[67] Nicolas AM, Pesic M, Engel E, Ziegler PK, Diefenhardt M, Kennel KB (2022). Inflammatory fibroblasts mediate resistance to neoadjuvant therapy in rectal cancer. Cancer Cell.

[68] Erez N, Truitt M, Olson P, Arron ST, Hanahan D (2010). Cancer-Associated Fibroblasts Are Activated in Incipient Neoplasia to Orchestrate Tumor-Promoting Inflammation in an NF-kappaB-Dependent Manner. Cancer Cell.

[69] Koh E-K, Lee H-R, Son W-C, Park G-Y, Kim J, Bae J-H (2023). Combinatorial immunotherapy with gemcitabine and ex vivo-expanded NK cells induces anti-tumor effects in pancreatic cancer. Sci Rep.

[70] Gürlevik E, Fleischmann-Mundt B, Brooks J, Demir IE, Steiger K, Ribback S (2016). Administration of Gemcitabine After Pancreatic Tumor Resection in Mice Induces an Antitumor Immune Response Mediated by Natural Killer Cells. Gastroenterology.

[71] Nielsen SR, Strøbech JE, Horton ER, Jackstadt R, Laitala A, Bravo MC (2021). Suppression of tumor-associated neutrophils by lorlatinib attenuates pancreatic cancer growth and improves treatment with immune checkpoint blockade. Nat Commun.

[72] Caronni N, La Terza F, Vittoria FM, Barbiera G, Mezzanzanica L, Cuzzola V (2023). IL-1β+ macrophages fuel pathogenic inflammation in pancreatic cancer. Nature.

[73] Kemp SB, Cheng N, Markosyan N, Sor R, Kim I-K, Hallin J (2023). Efficacy of a Small-Molecule Inhibitor of KrasG12D in Immunocompetent Models of Pancreatic Cancer. Cancer Discovery.

[74] Mahadevan KK, McAndrews KM, LeBleu VS, Yang S, Lyu H, Li B (2023). KRASG12D inhibition reprograms the microenvironment of early and advanced pancreatic cancer to promote FAS-mediated killing by CD8+ T cells. Cancer Cell.

[75] Wasko UN, Jiang J, Dalton TC, Curiel-Garcia A, Edwards AC, Wang Y (2024). Tumor-selective activity of RAS-GTP inhibition in pancreatic cancer. Nature.

