# Supplementary material for: SMAD4 and KRAS Status Shape Cancer Cell-Stromal Crosstalk and Therapeutic Response in Pancreatic Cancer
Source: Cancer Res. Author manuscript; Available in PMC 2025 Feb 9. (PMC7617379; doi:10.1158/0008-5472.CAN-24-2330)
Supplement: Supp Figures and legends [file EMS202804-supplement-Supp_Figures_and_legends.pdf]

SUPPLEMENTARY FIGURES AND FIGURE LEGENDS

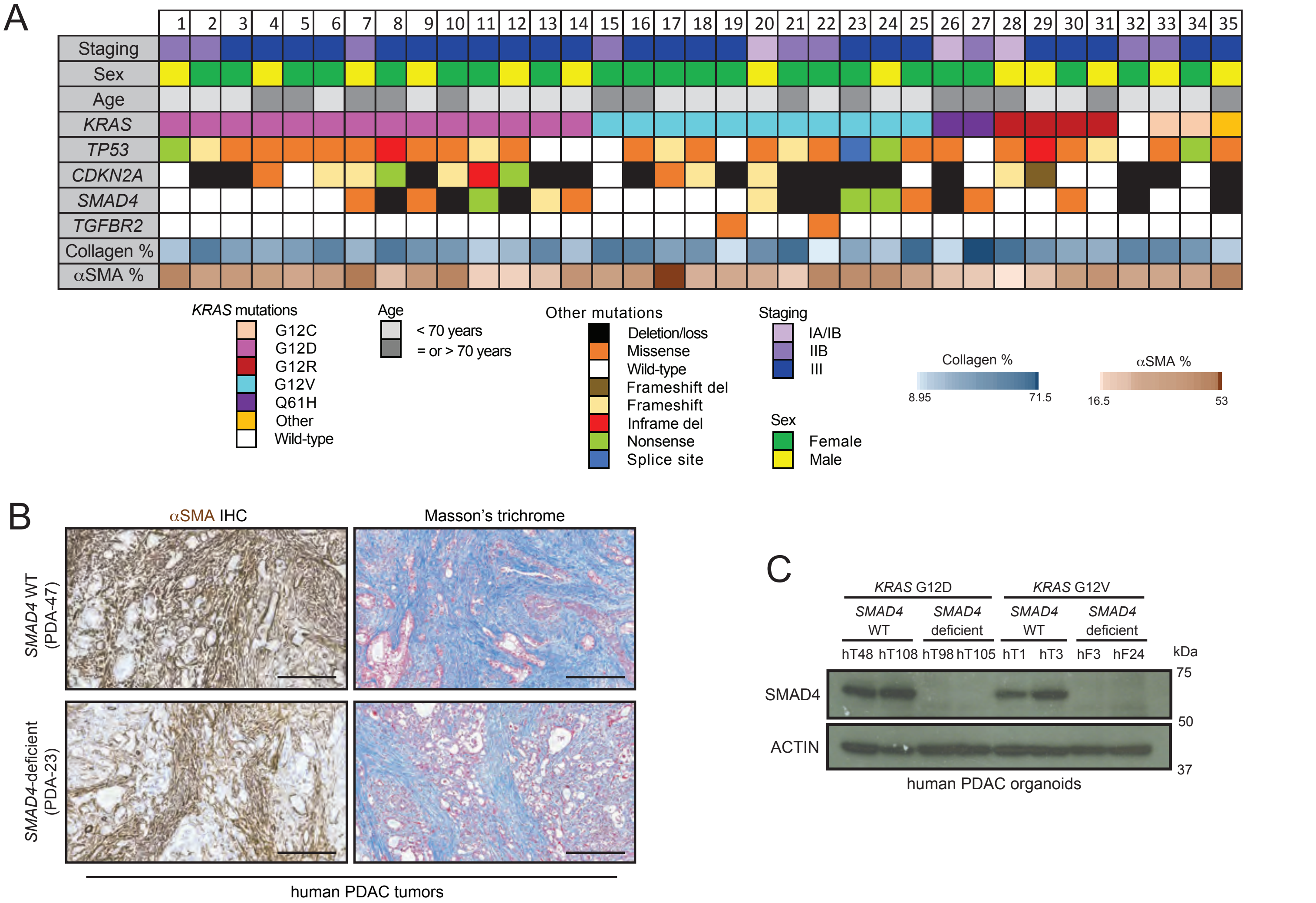

D

Mouse cohorts	Organoid lines	*KRAS* status	*TP53* status	*SMAD4* status	*CDKN2A* status
1-2 ♀/♂ NSG	hT1	G12V	R213*	WT	Deep loss
	hF24	G12V	F113V	Loss	Deep loss
3-4 ♀/♂ NSG	hT108	G12D	P98fs	WT	D108fs
	hT98	G12D	M237I	Deep loss	R80*
5 ♂NSG	hT3	G12V	Loss	WT	Deep loss
	hF3	G12V	R273L	Deep loss	R58*
6 ♂NSG	hT48	G12D	P151T	WT	Loss
	hT105	G12D	P142fs	Deep loss	Deep loss

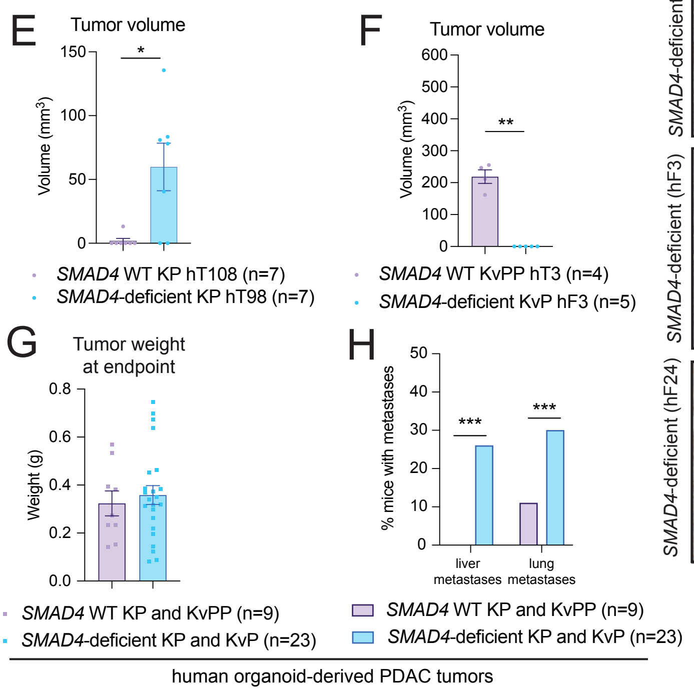

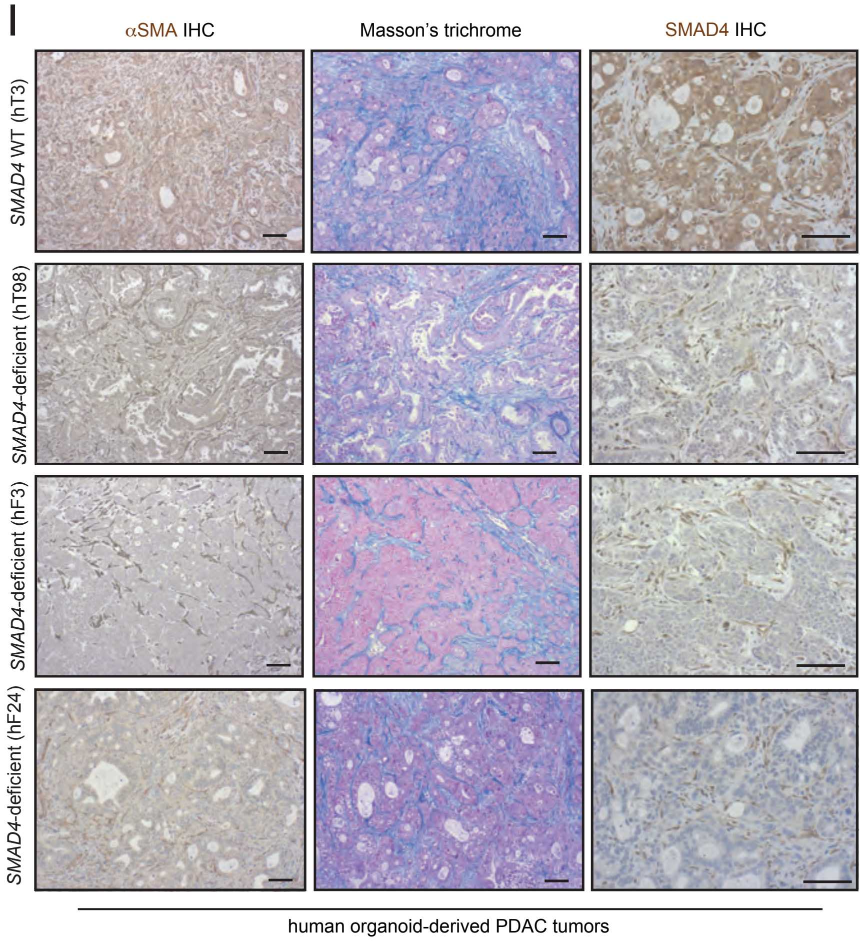

Supplementary Figure 1

Supplementary Figure 1. *SMAD4*-deficient human organoid-derived PDAC tumors have less fibrosis than *SMAD4* WT tumors. (A) Summary of clinical information, genetic alterations, collagen abundance and alpha smooth muscle actin (αSMA) abundance of a panel of human pancreatic ductal adenocarcinoma (PDAC) tumors (n=35). Masson's trichrome (for collagen) and αSMA quantification were calculated as % positive area/total PDAC area following annotation of the tissues by a pathologist. **(B)** Representative Masson's trichrome and αSMA stains (related to collagen and αSMA percentages, respectively, shown in panel A) in *SMAD4* WT or *SMAD4*-deficient human PDAC tumors. Scale bars, 300 μm. **(C)** Western blot analysis of SMAD4 in *SMAD4* WT or *SMAD4*-deficient human PDAC organoids with $KRAS^{G12D}$ or $KRAS^{G12V}$ mutation. ACTIN, loading control. This analysis confirmed presence or lack of protein expression in *SMAD4* WT or *SMAD4*-deficient organoids, respectively, validating what was previously reported in Tiriac et al *Cancer Discov* 2019 (see panel D). **(D)** Table summarizing the experimental cohorts of orthotopic transplantation models of the human PDAC organoids in NOD scid gamma (NSG) mice. The genetic information shown is from Tiriac et al *Cancer Discov* 2019. **(E-F)** Tumor volumes as measured by ultrasound-based imaging of tumors derived from the transplantation of *SMAD4* WT or *SMAD4*-deficient KP at day 83 post-transplant **(E)** or KvP (i.e., with mutant p53)/KvPP (i.e, with p53 null) at day 56 post-transplant **(F)** human PDAC organoids with $KRAS^{G12D}$ or $KRAS^{G12V}$ mutation, respectively. Results show mean $\pm$ SEM. *, $P < 0.05$; **, $P < 0.01$, Mann-Whitney test. **(G)** Weights measured at experimental endpoint of *SMAD4* WT KP and KvPP (70-217 days post-transplant) or *SMAD4*-deficient KP and KvP (62-301 days post-transplant) tumors in NSG mice. Results show mean $\pm$ SEM. No statistical difference was found, as calculated by Mann-Whitney test. *SMAD4* WT hT1 and hT108 organoids did not generate tumors and could not be included. All samples in this graph were analyzed by immunohistochemistry. **(H)** Percentage of *SMAD4* WT or *SMAD4*-deficient tumor-bearing NSG mice with metastases in the liver and lungs at experimental endpoint (same as panel G). No diaphragm metastases were found in either group. Results show mean $\pm$ SEM. ***, $P < 0.001$, chi-square test. *SMAD4* WT hT1 and hT108 organoids did not generate tumors and could not be included. **(I)** Representative Masson's trichrome, SMAD4 and αSMA stains in PDAC tumors derived from the orthotopic transplantation of *SMAD4* WT KvPP or *SMAD4*-deficient KP or KvP

human PDAC organoids. Scale bars, 50 μm. *SMAD4* WT hT1 and hT108 organoids did not generate tumors and could not be analyzed.

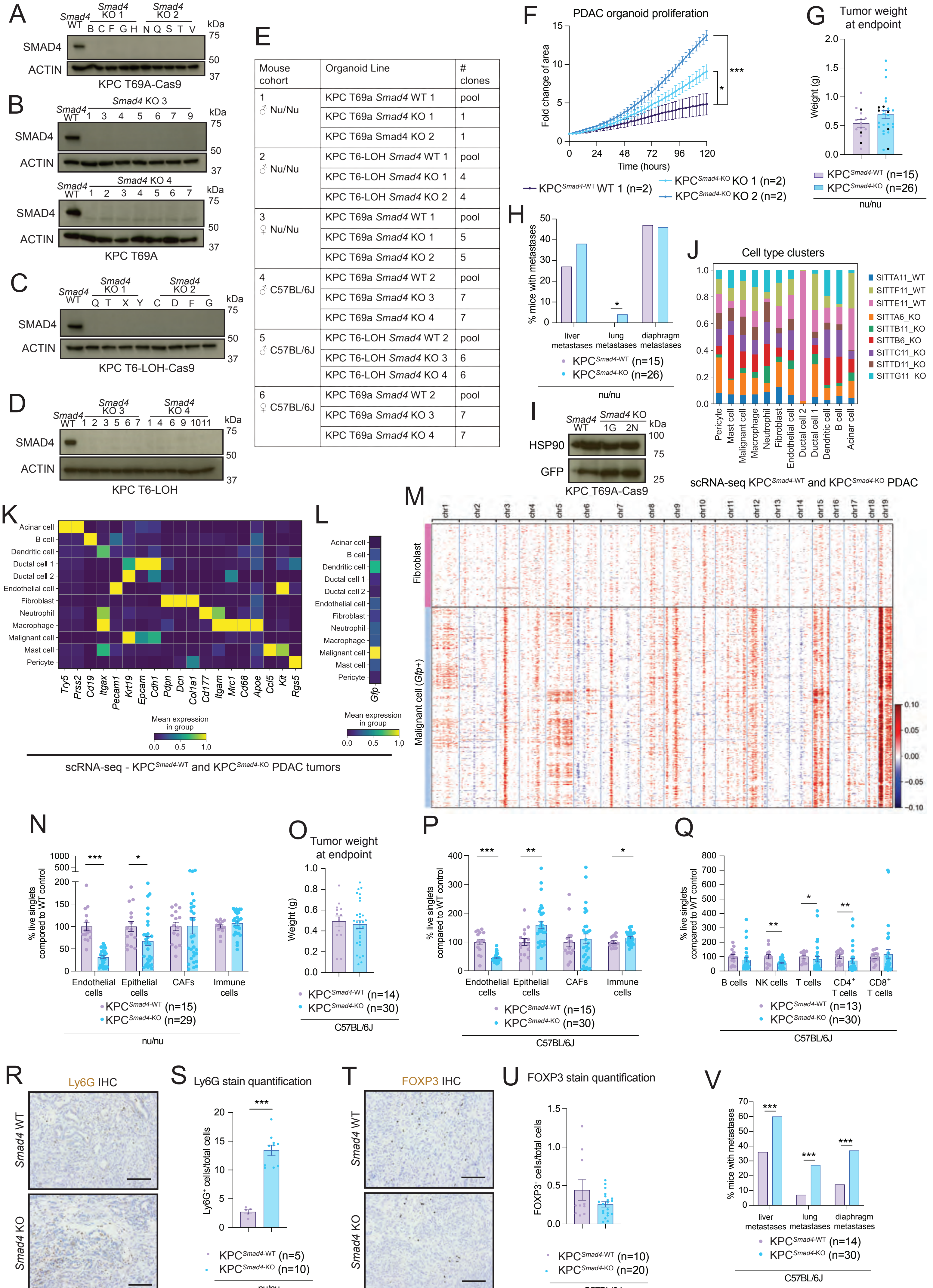

Mouse cohort	Organoid Line	# clones
1 ♂ Nu/Nu	KPC T69a *Smad4* WT 1	pool
	KPC T69a *Smad4* KO 1	1
	KPC T69a Smad4 KO 2	1
2 ♂ Nu/Nu	KPC T6-LOH *Smad4* WT 1	pool
	KPC T6-LOH *Smad4* KO 1	4
	KPC T6-LOH *Smad4* KO 2	4
3 ♀ Nu/Nu	KPC T69a *Smad4* WT 1	pool
	KPC T69a *Smad4* KO 1	5
	KPC T69a *Smad4* KO 2	5
4 ♂ C57BL/6J	KPC T69a *Smad4* WT 2	pool
	KPC T69a *Smad4* KO 3	7
	KPC T69a *Smad4* KO 4	7
5 ♂ C57BL/6J	KPC T6-LOH *Smad4* WT 2	pool
	KPC T6-LOH *Smad4* KO 3	6
	KPC T6-LOH *Smad4* KO 4	6
6 ♀ C57BL/6J	KPC T69a *Smad4* WT 2	pool
	KPC T69a *Smad4* KO 3	7
	KPC T69a *Smad4* KO 4	7

Supplementary Figure 2

Supplementary Figure 2. *Smad4* loss impacts the immune TME in KPC PDAC. (A-D) Validation of *Smad4* KO KPC PDAC organoids (n=2 parental lines) by western blot analysis of SMAD4 in KPC$^{Smad4\text{-WT}}$ (i.e. *Rosa26* KO) pools or KPC$^{Smad4\text{-KO}}$ clones (7-12 clones, from 3 different guides with 2 different CRISPR/Cas9-based methods) cultured in complete organoid media. ACTIN, loading controls. **(E)** Table summarizing the experimental cohorts of orthotopic transplantation models of KPC$^{Smad4\text{-WT}}$ and KPC$^{Smad4\text{-KO}}$ organoids in nu/nu or C57BL/6J mice. **(F)** Proliferation curves of KPC$^{Smad4\text{-WT}}$ or KPC$^{Smad4\text{-KO}}$ (KO1 and KO2 pools) PDAC organoids cultured for 120 hours in Matrigel in reduced media (i.e., 5% FBS DMEM). Data were normalized to the first measurement (at 3 hours post-plating on day 0). Results show mean ± SEM of n=2 biological replicates (with n=4 technical replicates each). *, $P < 0.05$; ***, $P < 0.001$, Mann-Whitney test calculated for the last time point. **(G)** Weights measured at experimental endpoint of KPC$^{Smad4\text{-WT}}$ (29-48 days post-transplant) or KPC$^{Smad4\text{-KO}}$ (21-50 days post-transplant) tumors in nu/nu mice. Results show mean ± SEM from 3 separate experiments, each with 1 WT group and 2 groups of KO pools from 2 different guides. No statistical difference was found, as calculated by Mann-Whitney test. All samples in this graph were analyzed by flow cytometry. Symbols in black denote samples also analyzed by single-cell RNA-sequencing (scRNA-seq). **(H)** Percentage of KPC$^{Smad4\text{-WT}}$ and KPC$^{Smad4\text{-KO}}$ nu/nu mice with metastases in the liver, lungs and diaphragm at experimental endpoint (same as panel G). Results are from 3 separate experiments, each with 1 WT group and 2 groups of KO pools from 2 different guides. *, $P < 0.05$, chi-square test. **(I)** Western blot analysis of green fluorescent protein (GFP) in KPC$^{Smad4\text{-WT}}$ pool or KPC$^{Smad4\text{-KO}}$ clones (from 2 different guides, see panel A) cultured in complete organoid media. HSP90, loading control. **(J)** Tumor sample contribution to different cell types in KPC$^{Smad4\text{-WT}}$ (n=3) or KPC$^{Smad4\text{-KO}}$ (n=6) PDAC, represented as bar plots showing proportions of the different tumor samples in each cell cluster, as analyzed by scRNA-seq. **(K)** Heatmap of scaled expression of cell type-specific markers in each cell cluster from KPC$^{Smad4\text{-WT}}$ or KPC$^{Smad4\text{-KO}}$ PDAC tumors, as analyzed by scRNA-seq. Data are scaled such that the cluster with the lowest average expression = 0 and the highest = 1 for each gene. **(L)** Heatmap of scaled expression of *Gfp* expression in each cell cluster from KPC$^{Smad4\text{-WT}}$ or KPC$^{Smad4\text{-KO}}$ tumors, as analyzed by scRNA-seq. Data are scaled such that the cluster with the lowest average expression = 0 and the highest = 1 for each gene. **(M)** Heatmap showing large-scale copy number variation (CNV) profile of the fibroblast and malignant cell clusters identified by scRNA-

seq. The color coding represents the CNV level based on a sliding window of 250 gene expression. Amplifications are shown in red and deletions are shown in blue. The fibroblast cluster was used as reference cell cluster. **(N)** Flow cytometric analysis of endothelial cells ($CD31^{+}CD45^{-}$), epithelial cells ($CD45^{-}CD31^{-}EpCAM^{+}$), CAFs ($CD45^{-}CD31^{-}EpCAM^{-}PDPN^{+}$) and immune cells ($CD45^{+}$) from live singlets in $KPC^{\mathit{Smad4}\text{-WT}}$ or $KPC^{\mathit{Smad4}\text{-KO}}$ tumors in nu/nu mice. Results show mean ± SEM from 3 separate experiments, each with 1 WT group and 2 groups of KO pools from 2 different guides. *, $P < 0.05$, ***, $P < 0.001$, Mann-Whitney test. **(O)** Weights measured at experimental endpoint of $KPC^{\mathit{Smad4}\text{-WT}}$ (26-111 days post-transplant) or $KPC^{\mathit{Smad4}\text{-KO}}$ (26-56 days post-transplant) tumors in C57BL/6J mice. Results show mean ± SEM from 3 separate experiments, each with 1 WT group and 2 groups of KO pools from 2 different guides. No statistical difference was found, as calculated by Mann-Whitney test. All samples in this graph were analyzed by flow cytometry. **(P)** Flow cytometric analysis of endothelial cells ($CD31^{+}CD45^{-}$), epithelial cells ($CD45^{-}CD31^{-}EpCAM^{+}$), CAFs ($CD45^{-}CD31^{-}EpCAM^{-}PDPN^{+}$) and immune cells ($CD45^{+}$) from live singlets in $KPC^{\mathit{Smad4}\text{-WT}}$ or $KPC^{\mathit{Smad4}\text{-KO}}$ tumors in C57BL/6J mice. Results show mean ± SEM from 3 separate experiments, each with 1 WT group and 2 groups of KO pools from 2 different guides. *, $P < 0.05$; **, $P < 0.01$; ***, $P < 0.001$, Mann-Whitney test. **(Q)** Flow cytometric analysis of B cells ($CD45^{+}CD19^{+}$), Natural Killer (NK) cells ($CD45^{+}NK1.1^{+}$), total T cells ($CD45^{+}CD3^{+}TCR\beta^{+}$), $CD4^{+}$ T cells ($CD45^{+}CD3^{+}TCR\beta^{+}CD8^{-}CD4^{+}$) and $CD8^{+}$ T cells ($CD45^{+}CD3^{+}TCR\beta^{+}CD4^{-}CD8^{+}$) from live singlets in $KPC^{\mathit{Smad4}\text{-WT}}$ or $KPC^{\mathit{Smad4}\text{-KO}}$ tumors in C57BL/6J mice. Results show mean ± SEM from 3 separate experiments, each with 1 WT group and 2 groups of KO pools from 2 different guides. *, $P < 0.05$; **, $P < 0.01$, Mann-Whitney test. **(R)** Representative immunohistochemistry (IHC) of the neutrophil marker Ly6G in $KPC^{\mathit{Smad4}\text{-WT}}$ and $KPC^{\mathit{Smad4}\text{-KO}}$ PDAC tumors in nu/nu mice. Scale bars, 50 μm. **(S)** Quantification of Ly6G stain in $KPC^{\mathit{Smad4}\text{-WT}}$ and $KPC^{\mathit{Smad4}\text{-KO}}$ PDAC tumors in nu/nu mice. Results show mean ± SEM. ***, $P < 0.001$, Mann-Whitney test. **(T)** Representative IHC of the T regulatory cell (Tregs) marker FOXP3 in $KPC^{\mathit{Smad4}\text{-WT}}$ and $KPC^{\mathit{Smad4}\text{-KO}}$ PDAC tumors in C57BL/6J mice. Scale bars, 50 μm. **(U)** Quantification of FOXP3 stain in $KPC^{\mathit{Smad4}\text{-WT}}$ and $KPC^{\mathit{Smad4}\text{-KO}}$ PDAC tumors in C57BL/6 mice. Results show mean ± SEM. No statistical difference was found, as calculated by Mann-Whitney test. **(V)** Percentage of $KPC^{\mathit{Smad4}\text{-WT}}$ and $KPC^{\mathit{Smad4}\text{-KO}}$ tumor-bearing C57BL/6J mice with metastases in the liver, lungs and diaphragm at experimental endpoint (same as panel O).

Results are from 3 separate experiments, each with 1 WT group and 2 groups of KO pools from 2 different guides. ***, $P < 0.001$, chi-square test.

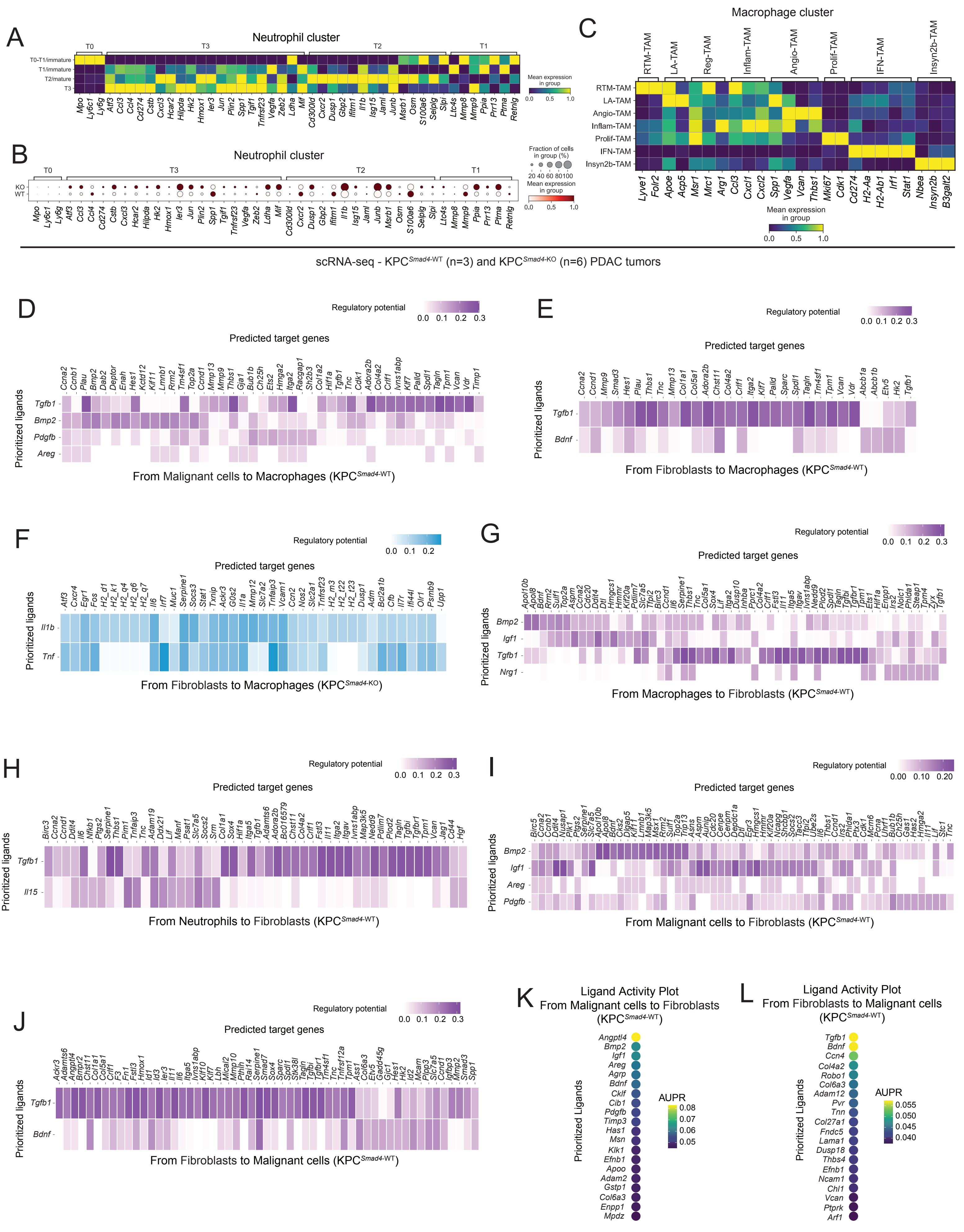

Supplementary Figure 3

Supplementary Figure 3. *Smad4* loss impacts malignant-stromal crosstalk in KPC PDAC. **(A)** Heatmap of scaled expression of neutrophil markers in distinct neutrophil sub-clusters from KPC$^{Smad4\text{-WT}}$ (n=3) and KPC$^{Smad4\text{-KO}}$ (n=6) tumors analyzed by scRNA-seq. Data are scaled such that the cluster with the lowest average expression = 0 and the highest = 1 for each gene. **(B)** Dot plot visualization of the scaled average expression of neutrophil subset markers in neutrophils from KPC$^{Smad4\text{-WT}}$ or KPC$^{Smad4\text{-KO}}$ tumors analyzed by scRNA-seq. The color intensity represents the expression level, and the size of the dots represents the percentage of expressing cells. **(C)** Heatmap of scaled expression of macrophage markers in distinct macrophage sub-clusters from KPC$^{Smad4\text{-WT}}$ and KPC$^{Smad4\text{-KO}}$ tumors analyzed by scRNA-seq. RTM-TAM, resident-tissue macrophage-like tumor-associated macrophage; LA-TAM, lipid-associated TAM; Angio-TAM, pro-angiogenic TAM; Inflam-TAM, inflammatory cytokine-enriched TAM; Prolif-TAM, proliferating TAM; IFN-TAM, interferon-primed TAM. Data are scaled such that the cluster with the lowest average expression = 0 and the highest = 1 for each gene. **(D)** Ligand-target heatmap shows top selected ligands of malignant cells inferred to regulate target genes in macrophages in KPC$^{Smad4\text{-WT}}$ PDAC, as assessed by NicheNet analysis of scRNA-seq. **(E)** Ligand-target heatmap shows top selected ligands of fibroblasts inferred to regulate target genes in macrophages in KPC$^{Smad4\text{-WT}}$ PDAC, as assessed by NicheNet analysis of scRNA-seq. **(F)** Ligand-target heatmap shows top selected ligands of fibroblasts inferred to regulate target genes in macrophages in KPC$^{Smad4\text{-KO}}$ PDAC, as assessed by NicheNet analysis of scRNA-seq. **(G)** Ligand-target heatmap shows top selected ligands of macrophages inferred to regulate target genes in fibroblasts in KPC$^{Smad4\text{-WT}}$ PDAC, as assessed by NicheNet analysis of scRNA-seq. **(H)** Ligand-target heatmap shows top selected ligands of neutrophils inferred to regulate target genes in fibroblasts in KPC$^{Smad4\text{-WT}}$ PDAC, as assessed by NicheNet analysis of scRNA-seq. **(I)** Ligand-target heatmap shows top selected ligands of malignant cells inferred to regulate target genes in fibroblasts in KPC$^{Smad4\text{-WT}}$ PDAC, as assessed by NicheNet analysis of scRNA-seq. **(J)** Ligand-target heatmap shows top selected ligands of fibroblasts inferred to regulate target genes in malignant cells in KPC$^{Smad4\text{-WT}}$ PDAC, as assessed by NicheNet analysis of scRNA-seq. **(K)** Ligand activity plot shows the top ligands of malignant cells inferred to regulate target genes in fibroblasts in KPC$^{Smad4\text{-WT}}$ PDAC, as assessed by NicheNet analysis of scRNA-seq. AUPR, area under the precision-recall curve. **(L)** Ligand activity plot shows the top ligands of fibroblasts

inferred to regulate target genes in malignant cells in KPC$^{\textit{Smad4}\text{-WT}}$ PDAC, as assessed by NicheNet analysis of scRNA-seq.

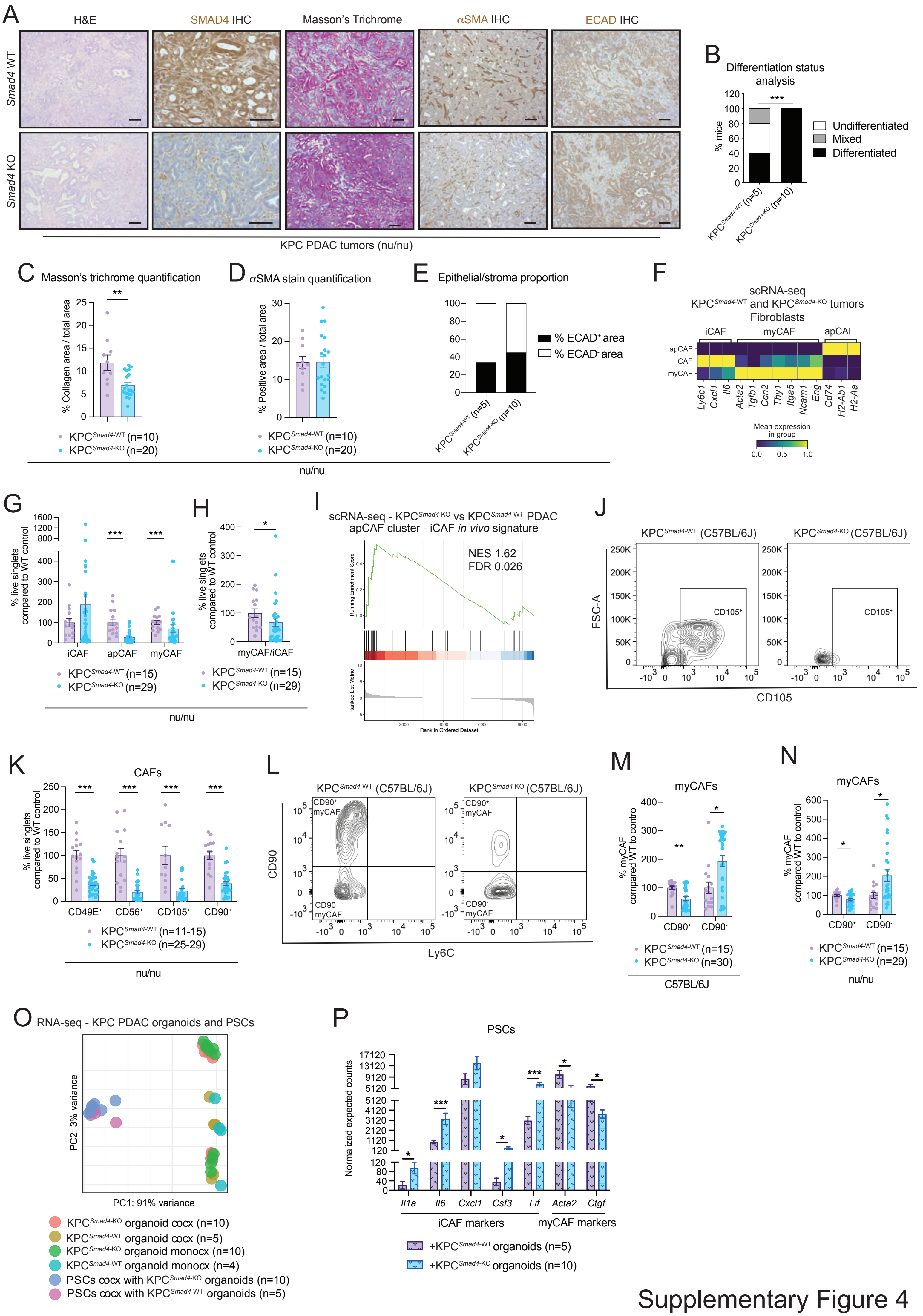

Supplementary Figure 4

Supplementary Figure 4. *Smad4* loss drives a fibro-inflammatory stroma in KPC PDAC. (A) Representative Hematoxylin & Eosin (H&E), SMAD4, Masson's trichrome, αSMA and E-cadherin (ECAD) stains in KPC$^{Smad4\text{-WT}}$ or KPC$^{Smad4\text{-KO}}$ PDAC tumors in nu/nu mice. Scale bars, 50 μm. **(B)** Analysis of tumor differentiation status in KPC$^{Smad4\text{-WT}}$ or KPC$^{Smad4\text{-KO}}$ tumors in nu/nu mice. ***, $P < 0.001$, chi-square test. **(C)** Quantification of Masson's trichrome stain in KPC$^{Smad4\text{-WT}}$ or KPC$^{Smad4\text{-KO}}$ tumors in nu/nu mice. Results show mean ± SEM from 2 separate experiments, each with 1 WT group and 2 groups of KO pools from 2 different guides. **, $P < 0.01$, Mann-Whitney test. **(D)** Quantification of αSMA stain in KPC$^{Smad4\text{-WT}}$ or KPC$^{Smad4\text{-KO}}$ tumors in nu/nu mice. Results show mean ± SEM from 2 separate experiments, each with 1 WT group and 2 groups of KO pools from 2 different guides. No statistical difference was found, as calculated by Mann-Whitney test. **(E)** Quantification of epithelial/stroma proportion (done by calculating the percentage of ECAD^{+} area) in KPC$^{Smad4\text{-WT}}$ or KPC$^{Smad4\text{-KO}}$ tumors in nu/nu mice. No statistical difference was found, as calculated by chi-square test. **(F)** Heatmap of scaled expression of CAF subtype markers in distinct CAF populations from KPC$^{Smad4\text{-WT}}$ (n=3) and KPC$^{Smad4\text{-KO}}$ (n=6) tumors analyzed by scRNA-seq. Data are scaled such that the cluster with the lowest average expression = 0 and the highest = 1 for each gene. **(G)** Flow cytometric analysis of myCAFs (Ly6C^{-}MHCII^{-} CAFs), iCAFs (Ly6C^{+}MHCII^{-} CAFs) and apCAFs (Ly6C^{-}MHCII^{+} CAFs) from live singlets in KPC$^{Smad4\text{-WT}}$ or KPC$^{Smad4\text{-KO}}$ tumors in nu/nu mice. Results show mean ± SEM from 3 separate experiments, each with 1 WT group and 2 groups of KO pools from 2 different guides. ***, $P < 0.001$, Mann-Whitney test. **(H)** Flow cytometric analysis of myCAF/iCAF ratio from live singlets in KPC$^{Smad4\text{-WT}}$ or KPC$^{Smad4\text{-KO}}$ tumors in nu/nu mice. Results show mean ± SEM from 3 separate experiments, each with 1 WT group and 2 groups of KO pools from 2 different guides. *, $P < 0.05$, Mann-Whitney test. **(I)** Gene set enrichment analysis (GSEA) of iCAF *in vivo* signature in apCAFs from KPC$^{Smad4\text{-KO}}$ PDAC compared to apCAFs from KPC$^{Smad4\text{-WT}}$ PDAC, as assessed by scRNA-seq. The signature is significantly upregulated. NES, normalized enrichment score. FDR, false discovery rate. The iCAF *in vivo* signature is from Elyada et al *Cancer Discov* 2019. **(J)** Representative flow plots of CD105^{+} CAFs from KPC$^{Smad4\text{-WT}}$ or KPC$^{Smad4\text{-KO}}$ tumors in C57BL/6J mice. **(K)** Flow cytometric analyses of CD90^{+}, CD49E^{+}, CD56^{+} and CD105^{+} CAFs from live singlets in KPC$^{Smad4\text{-WT}}$ or KPC$^{Smad4\text{-KO}}$ tumors in nu/nu mice. Results show mean ± SEM from 3 separate experiments, each with 1 WT group and 2 groups of KO pools from 2 different guides. ***, $P < 0.001$, Mann-Whitney test. **(L)** Representative flow

plots of $CD90^-$ and $CD90^+$ myCAFs ($Ly6C^-MHCII^-$ CAFs) from $KPC^{Smad4\text{-}WT}$ or $KPC^{Smad4\text{-}KO}$ tumors in C57BL/6J mice. **(M)** Flow cytometric analysis of $CD90^-$ and $CD90^+$ myCAFs ($Ly6C^-MHCII^-$ CAFs) from the parental myCAF gate in $KPC^{Smad4\text{-}WT}$ or $KPC^{Smad4\text{-}KO}$ tumors in C57BL/6J mice. Results show mean ± SEM from 3 separate experiments, each with 1 WT group and 2 groups of KO pools from 2 different guides. *, $P < 0.05$; **, $P < 0.01$, Mann-Whitney test. **(N)** Flow cytometric analysis of $CD90^-$ and $CD90^+$ myCAFs ($Ly6C^-MHCII^-$ CAFs) from the parental myCAF gate in $KPC^{Smad4\text{-}WT}$ or $KPC^{Smad4\text{-}KO}$ tumors in nu/nu mice. Results show mean ± SEM from 3 separate experiments, each with 1 WT group and 2 groups of KO pools from 2 different guides. *, $P < 0.05$, Mann-Whitney test. **(O)** Principal component analysis (PCA) of pancreatic stellate cells (PSCs) and KPC PDAC organoids flow-sorted from co-cultures and monocultures analyzed by RNA-sequencing (RNA-seq). Cocx, co-culture; monocx, monoculture. **(P)** RNA-seq expression of iCAF and myCAF markers in PSCs flow-sorted from co-cultures with $KPC^{Smad4\text{-}WT}$ or $KPC^{Smad4\text{-}KO}$ organoids. Results show mean ± SEM. *, $P < 0.05$; ***, $P < 0.001$, Mann-Whitney test.

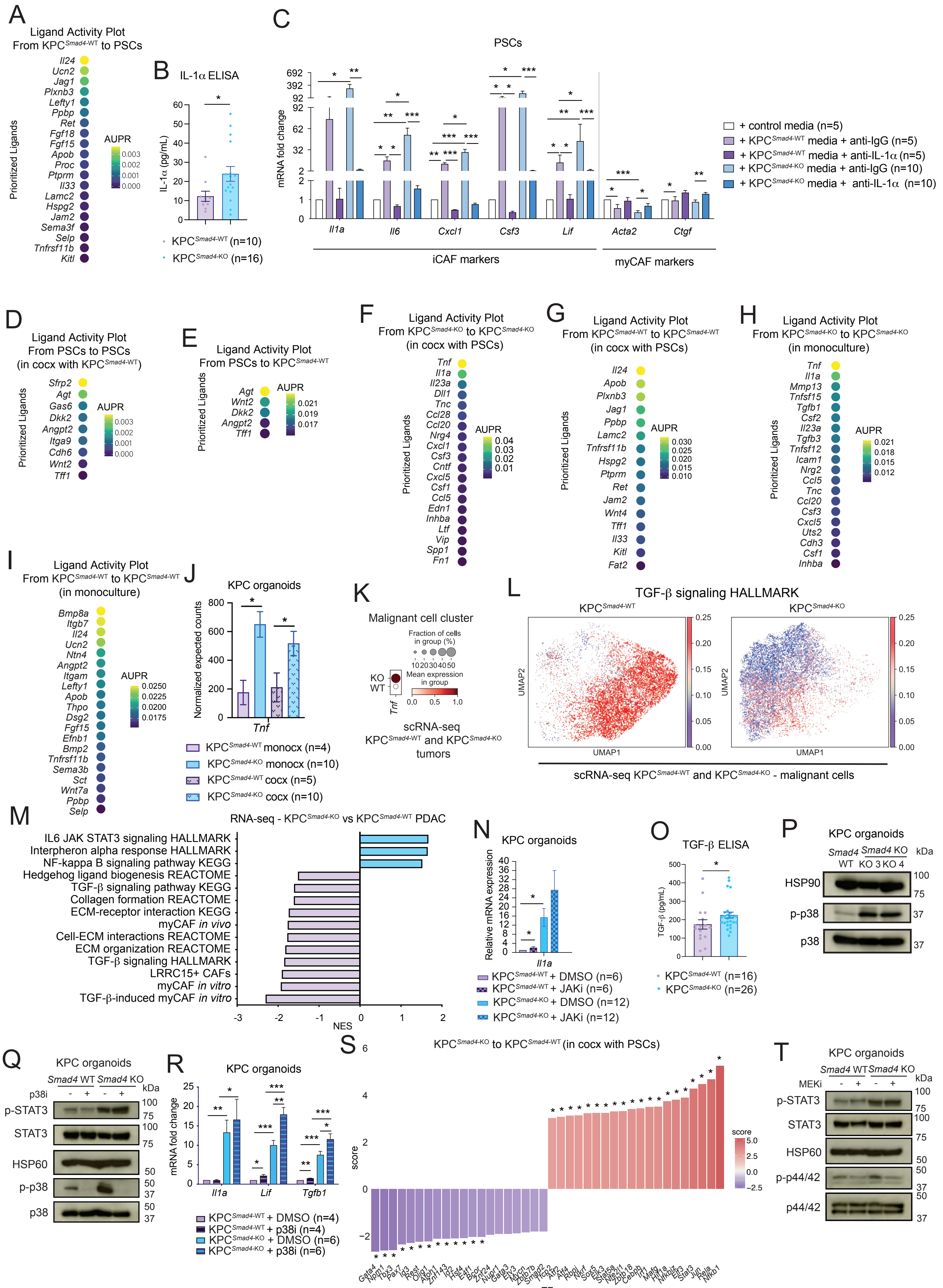

Supplementary Figure 5

Supplementary Figure 5. *Smad4* loss upregulates IL-1 and JAK/STAT signaling in KPC PDAC. (A) Ligand activity plot shows the top ligands from KPC$^{\mathit{Smad4}\text{-WT}}$ organoids inferred to regulate target genes in co-cultured PSCs, as assessed by NicheNet analysis of RNA-seq. **(B)** ELISA of IL-1α in KPC$^{\mathit{Smad4}\text{-WT}}$ or KPC$^{\mathit{Smad4}\text{-KO}}$ organoid-conditioned media. Results show mean ± SEM of n=5-8 biological replicates, each in 2 technical replicates. *, $P < 0.05$, Mann-Whitney test. **(C)** qPCR analysis of iCAF (*Il1a*, *Lif*, *Il6*, *Cxcl1*, *Csf3*) and myCAF (*Acta2, Ctgf)* markers in PSCs cultured for 4 days in Matrigel in reduced media or conditioned media from KPC$^{\mathit{Smad4}\text{-WT}}$ or KPC$^{\mathit{Smad4}\text{-KO}}$ organoids and in the presence of 5 μg/mL anti-IL1-α or isotype control. Results show mean ± SEM. *, $P < 0.05$; **, $P < 0.01$; ***, $P < 0.001$, paired and unpaired Student's *t* test. **(D)** Ligand activity plot shows the top ligands from PSCs co-cultured with KPC$^{\mathit{Smad4}\text{-WT}}$ organoids inferred to regulate target genes in PSCs, as assessed by NicheNet analysis of RNA-seq. **(E)** Ligand activity plot shows the top ligands from PSCs inferred to regulate target genes in co-cultured KPC$^{\mathit{Smad4}\text{-WT}}$ PDAC organoids, as assessed by NicheNet analysis of RNA-seq. **(F)** Ligand activity plot shows the top ligands from KPC$^{\mathit{Smad4}\text{-KO}}$ organoids in co-culture with PSCs inferred to regulate target genes in organoids, as assessed by NicheNet analysis of RNA-seq. **(G)** Ligand activity plot shows the top ligands from KPC$^{\mathit{Smad4}\text{-WT}}$ organoids in co-culture with PSCs inferred to regulate target genes in organoids, as assessed by NicheNet analysis of RNA-seq. **(H)** Ligand activity plot shows the top ligands from KPC$^{\mathit{Smad4}\text{-KO}}$ organoids in monoculture inferred to regulate target genes in organoids, as assessed by NicheNet analysis of RNA-seq. **(I)** Ligand activity plot shows the top ligands from KPC$^{\mathit{Smad4}\text{-WT}}$ organoids in monoculture inferred to regulate target genes in organoids, as assessed by NicheNet analysis of RNA-seq. **(J)** RNA-seq expression of *Tnf* in KPC$^{\mathit{Smad4}\text{-WT}}$ and KPC$^{\mathit{Smad4}\text{-KO}}$ PDAC organoids flow-sorted from monocultures and co-cultures with PSCs. Results show mean ± SEM. *, $P < 0.05$, Mann-Whitney test. **(K)** Dot plot visualization of the scaled average expression of *Tnf* in malignant cells of KPC$^{\mathit{Smad4}\text{-WT}}$ (n=3) or KPC$^{\mathit{Smad4}\text{-KO}}$ (n=6) PDAC tumors, as analyzed by scRNA-seq. The color intensity represents the expression level, and the size of the dots represents the percentage of expressing cells. **(L)** Uniform manifold approximation and projection (UMAP) plots of malignant cells from KPC$^{\mathit{Smad4}\text{-WT}}$ or KPC$^{\mathit{Smad4}\text{-KO}}$ tumors colored by the normalized expression score of the TGF-β signaling HALLMARK pathway, as analyzed by scRNA-seq. **(M)** Selected significantly upregulated (i.e. NES > 1.50 and FDR < 0.25) and downregulated (i.e. NES < -1.50 and FDR < 0.25) pathways identified by GSEA of KPC$^{\mathit{Smad4}\text{-KO}}$ (n=5) compared to KPC$^{\mathit{Smad4}\text{-WT}}$ (n=3) PDAC

tumors in C57BL/6J mice, as assessed by RNA-seq. **(N)** qPCR analysis of *Il1a* in KPC$^{Smad4\text{-WT}}$ or KPC$^{Smad4\text{-KO}}$ organoids cultured for 3 days in reduced media with or without 8 μg/mL JAK inhibitor AZD1480 (JAKi). Results show mean ± SEM. *, $P < 0.05$, paired and unpaired Student's *t* test. **(O)** ELISA of TGF-β in KPC$^{Smad4\text{-WT}}$ or KPC$^{Smad4\text{-KO}}$ organoid-conditioned media. Results show mean ± SEM of n=8-13 biological replicates, each in 2 technical replicates. *, $P < 0.05$, Mann-Whitney test. **(P)** Western blot analysis of phospho-p38 (p-p38) and p38 in KPC$^{Smad4\text{-WT}}$ or KPC$^{Smad4\text{-KO}}$ organoids cultured for 3 days in reduced media. HSP90, loading control. **(Q)** Western blot analysis of phospho-STAT3 (p-STAT3), STAT3, p-p38 and p38 in KPC$^{Smad4\text{-WT}}$ or KPC$^{Smad4\text{-KO}}$ organoids cultured for 3 days in reduced media with 1 μM p38 inhibitor pexmetinib (p38i). HSP60, loading control. **(R)** qPCR analysis of *Il1a*, *Lif*, and *Tgfb1* in KPC$^{Smad4\text{-WT}}$ or KPC$^{Smad4\text{-KO}}$ organoids cultured for 3 days in reduced media with or without 1 μM p38i. Results show mean ± SEM. *, $P < 0.05$; **, $P < 0.01$; ***, $P < 0.001$, paired and unpaired Student's *t* test. **(S)** Bar plot shows the activity score of the top 20 most activated and most deactivated transcription factors (TFs) in KPC$^{Smad4\text{-KO}}$ co-cultured with PSCs (vs KPC$^{Smad4\text{-WT}}$ co-cultured with PSCs). TFs with significant activity (*, $p < 0.05$) are annotated. **(T)** Western blot analysis of p-STAT3, STAT3, phospho-p44/42 (p-p44/42) and p44/42 in KPC$^{Smad4\text{-WT}}$ or KPC$^{Smad4\text{-KO}}$ organoids cultured for 3 days in reduced media with 2 nM MEK inhibitor trametinib (MEKi). HSP60, loading control.

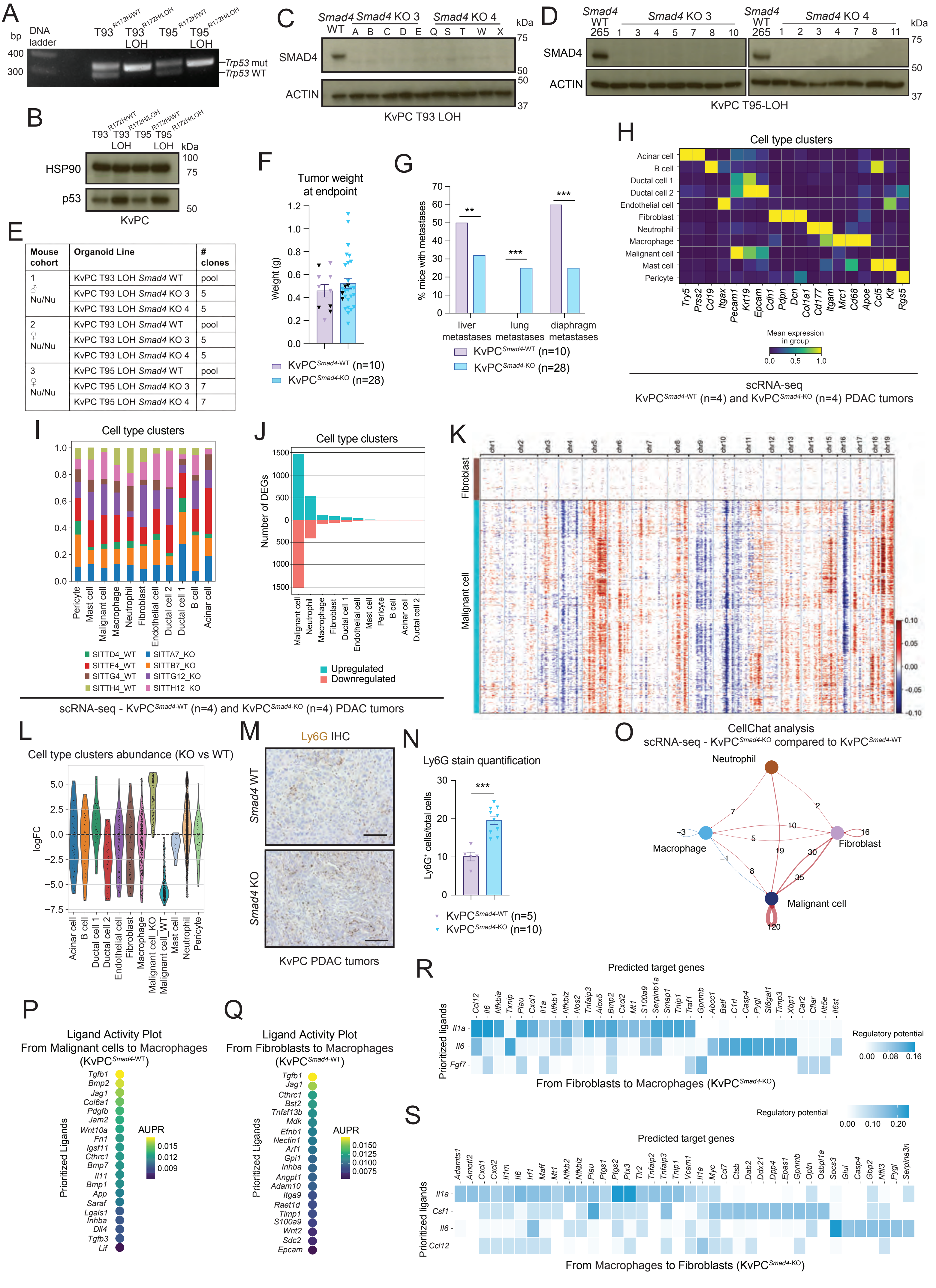

Mouse cohort	Organoid Line	# clones
1 ♂ Nu/Nu	KvPC T93 LOH *Smad4* WT	pool
	KvPC T93 LOH *Smad4* KO 3	5
	KvPC T93 LOH *Smad4* KO 4	5
2 ♀ Nu/Nu	KvPC T93 LOH *Smad4* WT	pool
	KvPC T93 LOH *Smad4* KO 3	5
	KvPC T93 LOH *Smad4* KO 4	5
3 ♀ Nu/Nu	KvPC T95 LOH *Smad4* WT	pool
	KvPC T95 LOH *Smad4* KO 3	7
	KvPC T95 LOH *Smad4* KO 4	7

Supplementary Figure 6

Supplementary Figure 6. *Smad4* loss impacts the immune TME and malignant-stromal crosstalk in KvPC PDAC. **(A)** DNA gel showing *Trp53* status in T93 and T95 KvPC PDAC organoids with or without p53 loss of heterozygosity (LOH). WT, wild-type. mut, mutant. **(B)** Western blot analysis of p53 in T93 and T95 KvPC PDAC organoids with or without p53 LOH. HSP90, loading control. **(C-D)** Validation of *Smad4* KO KvPC PDAC organoids by western blot analysis of SMAD4 in $KvPC^{Smad4\text{-}WT}$ (i.e. *Rosa26* KO pools) or $KvPC^{Smad4\text{-}KO}$ clones from 2 parental lines (10-14 clones from 2 different guides) cultured in complete organoid media. ACTIN, loading controls. **(E)** Table summarizing the experimental cohorts of orthotopic transplantation models of KvPC PDAC organoids in nu/nu mice. **(F)** Weights measured at experimental endpoint of $KvPC^{Smad4\text{-}WT}$ (76-154 days post-transplant) or $KvPC^{Smad4\text{-}KO}$ (35-122 days post-transplant) tumors in nu/nu mice. Results show mean ± SEM from 3 separate experiments, each with 1 WT group and 2 groups of KO pools from 2 different guides. No statistical difference was found, as calculated by Mann-Whitney test. All samples in this graph were analyzed by flow cytometry. Symbols in black denote samples also analyzed by scRNA-seq. **(G)** Percentage of $KvPC^{Smad4\text{-}WT}$ and $KvPC^{Smad4\text{-}KO}$ tumor-bearing nu/nu mice with metastases in the liver, lungs and diaphragm at experimental endpoint (same as panel F). Results are from 3 separate experiments, each with 1 WT group and 2 groups of KO pools from 2 different guides. **, $P < 0.01$; ***, $P < 0.001$, chi-square test. **(H)** Heatmap of scaled expression of cell type-specific markers in each cell cluster of $KvPC^{Smad4\text{-}WT}$ (n=4) or $KvPC^{Smad4\text{-}KO}$ (n=4) PDAC tumors, as analyzed by scRNA-seq. Data are scaled such that the cluster with the lowest average expression = 0 and the highest = 1 for each gene. **(I)** Tumor sample contribution in $KvPC^{Smad4\text{-}WT}$ or $KvPC^{Smad4\text{-}KO}$ tumors, represented as bar plots showing proportions of the different tumor samples in each cell cluster, as assessed by scRNA-seq. **(J)** Upregulated and downregulated differentially expressed genes (DEGs) in each cell type identified by pseudobulk analysis from scRNA-seq of $KvPC^{Smad4\text{-}WT}$ or $KvPC^{Smad4\text{-}KO}$ tumors. FDR < 0.05. **(K)** Heatmap showing large-scale CNV profile of the fibroblast and malignant cell clusters in KvPC tumors, as identified by scRNA-seq. The color coding represents the CNV level based on a sliding window of 250 gene expression. Amplifications are shown in red and deletions are shown in blue. The fibroblast cluster was used as reference cell cluster. **(L)** Violin plots showing the distribution of groups of nearest neighbor cells from different cell type clusters upon the log-fold change between $KvPC^{Smad4\text{-}KO}$ vs $KvPC^{Smad4\text{-}WT}$ conditions computed with Milo, as assessed by scRNA-

seq. The malignant cell cluster was divided in cells from *Smad4* WT or *Smad4* KO tumors to clarify the directionality of abundance (i.e., significantly enriched in WT or KO, respectively). **(M)** Representative Ly6G IHC stain in KvPC$^{Smad4\text{-WT}}$ and KvPC$^{Smad4\text{-KO}}$ PDAC tumors. Scale bars, 50 μm. **(N)** Quantification of Ly6G stain in KvPC$^{Smad4\text{-WT}}$ and KvPC$^{Smad4\text{-KO}}$ PDAC tumors. Results show mean ± SEM. ***, P < 0.001, Mann-Whitney test. **(O)** Cell-cell communication analysis using CellChat showing the number of connections lost (in blue) or gained (in red) between malignant cells, fibroblasts, macrophages and neutrophils in KvPC$^{Smad4\text{-KO}}$ tumors compared to KvPC$^{Smad4\text{-WT}}$ tumors, as assessed by scRNA-seq. **(P)** Ligand activity plot shows the top ligands of malignant cells inferred to regulate target genes in macrophages in KvPC$^{Smad4\text{-WT}}$ PDAC, as assessed by NicheNet analysis of scRNA-seq. **(Q)** Ligand activity plot shows the top ligands of fibroblasts inferred to regulate target genes in macrophages in KvPC$^{Smad4\text{-WT}}$ PDAC, as assessed by NicheNet analysis of scRNA-seq. **(R)** Ligand-target heatmap shows top selected ligands of fibroblasts inferred to regulate target genes in macrophages in KvPC$^{Smad4\text{-KO}}$ PDAC, as assessed by NicheNet analysis of scRNA-seq. **(S)** Ligand-target heatmap shows top selected ligands of macrophages inferred to regulate target genes in fibroblasts in KvPC$^{Smad4\text{-KO}}$ PDAC, as assessed by NicheNet analysis of scRNA-seq.

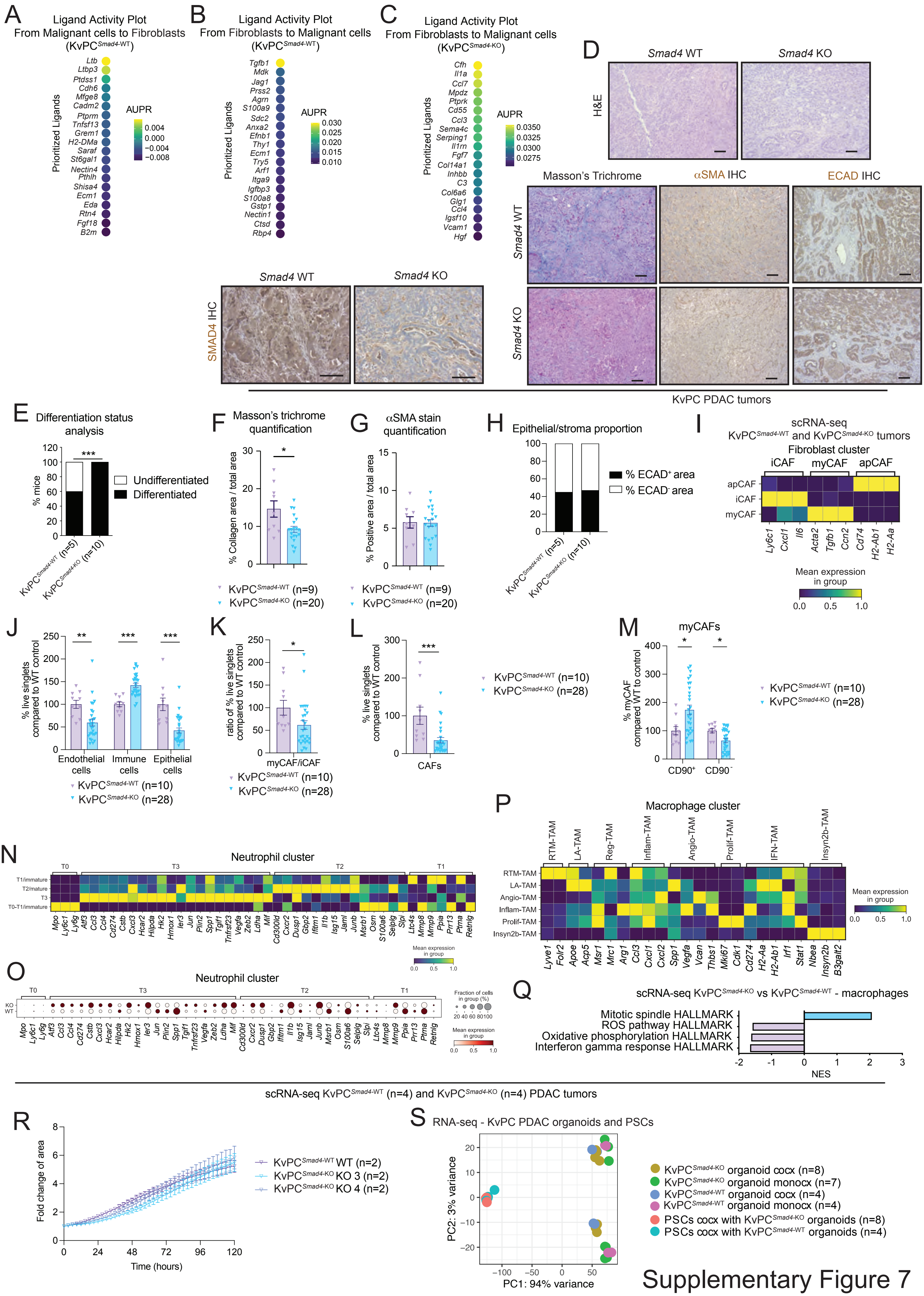

Supplementary Figure 7

Supplementary Figure 7. *Smad4* loss drives a fibro-inflammatory stroma in KvPC PDAC. (A) Ligand activity plot shows the top ligands of malignant cells inferred to regulate target genes in fibroblasts in KvPC$^{Smad4\text{-WT}}$ PDAC, as assessed by NicheNet analysis of scRNA-seq. **(B)** Ligand activity plot shows the top ligands of fibroblasts inferred to regulate target genes in malignant cells in KvPC$^{Smad4\text{-WT}}$ PDAC, as assessed by NicheNet analysis of scRNA-seq. **(C)** Ligand activity plot shows the top ligands of fibroblasts inferred to regulate target genes in malignant cells in KvPC$^{Smad4\text{-KO}}$ PDAC, as assessed by NicheNet analysis of scRNA-seq. **(D)** Representative H&E, SMAD4, Masson's trichrome, αSMA and ECAD stains in KvPC$^{Smad4\text{-WT}}$ or KvPC$^{Smad4\text{-KO}}$ tumors. Scale bars, 50 μm. **(E)** Analysis of tumor differentiation status in KvPC$^{Smad4\text{-WT}}$ or KvPC$^{Smad4\text{-KO}}$ tumors. ***, P < 0.001, chi-square test. **(F)** Quantification of Masson's trichrome stain in KvPC$^{Smad4\text{-WT}}$ or KvPC$^{Smad4\text{-KO}}$ tumors. Results show mean ± SEM from 2 separate experiments, each with 1 WT group and 2 groups of KO pools from 2 different guides. *, P < 0.05, Mann-Whitney test. **(G)** Quantification of αSMA stain in KvPC$^{Smad4\text{-WT}}$ or KvPC$^{Smad4\text{-KO}}$ tumors. Results show mean ± SEM from 2 separate experiments, each with 1 WT group and 2 groups of KO pools from 2 different guides. No statistical difference was found, as calculated by Mann-Whitney test. **(H)** Quantification of epithelial/stroma proportion (done by calculating the percentage of ECAD$^+$ area) in KvPC$^{Smad4\text{-WT}}$ or KvPC$^{Smad4\text{-KO}}$ tumors. No statistical difference was found, as calculated by chi-square test. **(I)** Heatmap of scaled expression of CAF subtype-specific markers in each CAF cluster of KvPC$^{Smad4\text{-WT}}$ (n=4) or KvPC$^{Smad4\text{-KO}}$ (n=4) tumors, as analyzed by scRNA-seq. Data are scaled such that the cluster with the lowest average expression = 0 and the highest = 1 for each gene. **(J)** Flow cytometric analysis of endothelial cells (CD31$^+$CD45$^-$), epithelial cells (CD45$^-$CD31$^-$EpCAM$^+$) and immune cells (CD45$^+$) from live singlets in KvPC$^{Smad4\text{-WT}}$ or KvPC$^{Smad4\text{-KO}}$ tumors. Results show mean ± SEM from 3 separate experiments, each with 1 WT group and 2 groups of KO pools from 2 different guides. **, P < 0.01, ***, P < 0.001, Mann-Whitney test. **(K)** myCAF/iCAF ratio from live singlets in KvPC$^{Smad4\text{-WT}}$ or KvPC$^{Smad4\text{-KO}}$ tumors. Results show mean ± SEM from 2 separate experiments, each with 1 WT group and 2 groups of KO pools from 2 different guides. *, P < 0.05, Mann-Whitney test. **(L)** Flow cytometric analysis of CAFs (CD45$^-$CD31$^-$EpCAM$^-$PDPN$^+$) from live singlets in KvPC$^{Smad4\text{-WT}}$ or KvPC$^{Smad4\text{-KO}}$ tumors. Results show mean ± SEM from 3 separate experiments, each with 1 WT group and 2 groups of KO pools from 2 different guides. ***, P < 0.001, Mann-Whitney test. **(M)** Flow cytometric analysis of CD90$^-$ and CD90$^+$ myCAFs

(Ly6C$^-$MHCII$^-$ CAFs) from the parental myCAF gate in KvPC$^{Smad4\text{-WT}}$ or KvPC$^{Smad4\text{-KO}}$ tumors. Results show mean ± SEM from 3 separate experiments, each with 1 WT group and 2 groups of KO pools from 2 different guides. *, P < 0.05, Mann-Whitney test. **(N)** Heatmap of scaled expression of neutrophil markers in distinct neutrophil sub-clusters from KvPC$^{Smad4\text{-WT}}$ and KvPC$^{Smad4\text{-KO}}$ tumors analyzed by scRNA-seq. Data are scaled such that the cluster with the lowest average expression = 0 and the highest = 1 for each gene. **(O)** Dot plot visualization of the scaled average expression of neutrophil markers in neutrophils from KvPC$^{Smad4\text{-WT}}$ or KvPC$^{Smad4\text{-KO}}$ tumors analyzed by scRNA-seq. The color intensity represents the expression level, and the size of the dots represents the percentage of expressing cells. **(P)** Heatmap of scaled expression of macrophage markers in distinct macrophage sub-clusters from KvPC$^{Smad4\text{-WT}}$ and KvPC$^{Smad4\text{-KO}}$ tumors analyzed by scRNA-seq. Data are scaled such that the cluster with the lowest average expression = 0 and the highest = 1 for each gene. **(Q)** Selected significantly upregulated (i.e. NES > 1.50 and FDR < 0.25) and downregulated (i.e. NES < -1.50 and FDR < 0.25) pathways identified by GSEA of macrophages from KvPC$^{Smad4\text{-KO}}$ compared to KvPC$^{Smad4\text{-WT}}$ tumors, as assessed by pseudobulk analysis from the scRNA-seq dataset. **(R)** Proliferation curves of KvPC$^{Smad4\text{-WT}}$ or KvPC$^{Smad4\text{-KO}}$ PDAC organoids cultured for 96 hours in Matrigel in reduced media. Data were normalized to the first measurement (at 3 hours post-plating on day 0). Results show mean ± SEM of n=2 biological replicates (with n=4 technical replicates each). No statistical difference was found, as calculated by Mann-Whitney test for the last time point. **(S)** PCA of PSCs and organoids flow-sorted from monocultures or co-cultures of KvPC PDAC organoids with PSCs.

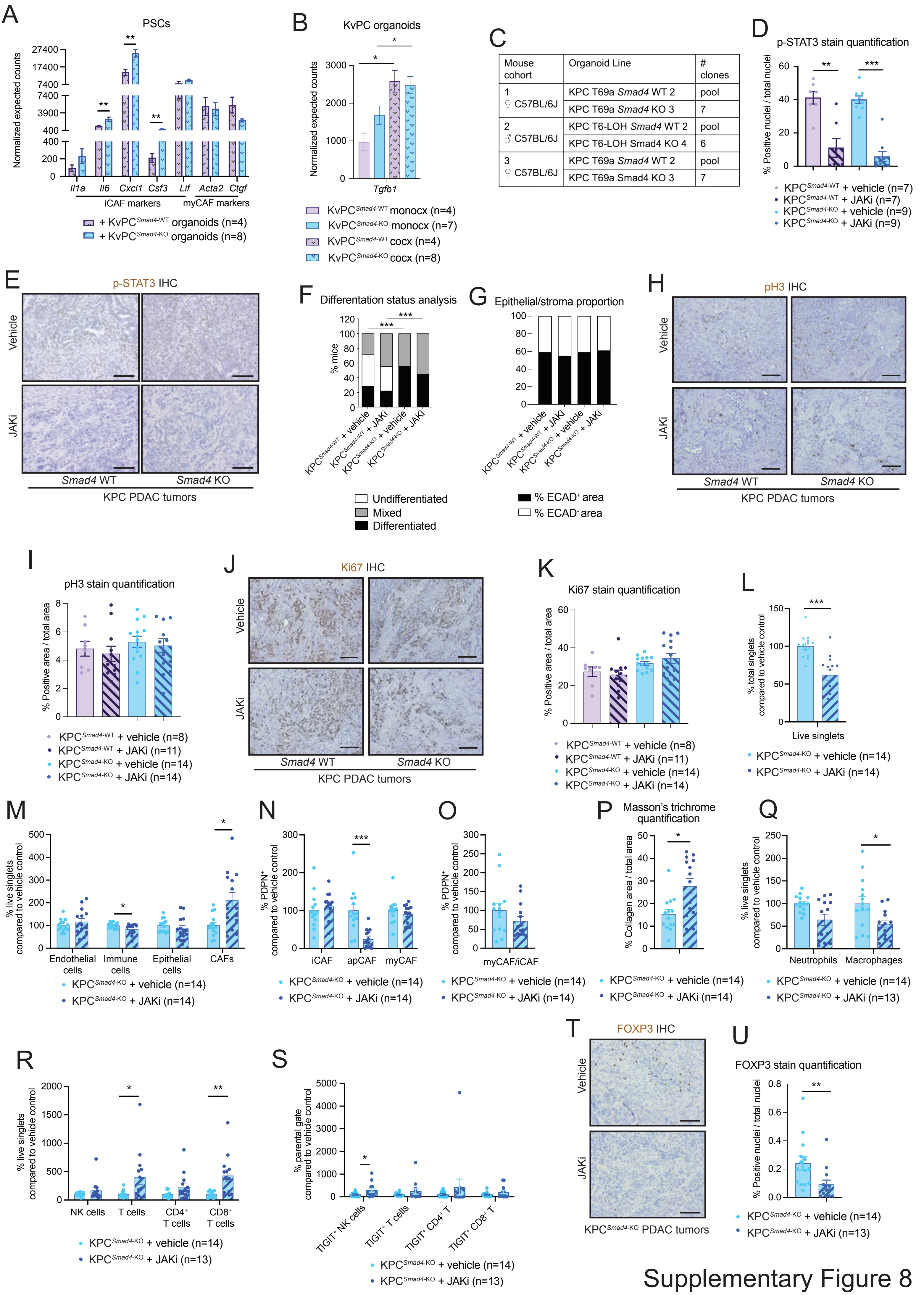

Mouse cohort	Organoid Line	# clones
1 ♀ C57BL/6J	KPC T69a *Smad4* WT 2	pool
	KPC T69a *Smad4* KO 3	7
2 ♂ C57BL/6J	KPC T6-LOH *Smad4* WT 2	pool
	KPC T6-LOH Smad4 KO 4	6
3 ♀ C57BL/6J	KPC T69a *Smad4* WT 2	pool
	KPC T69a Smad4 KO 3	7

Supplementary Figure 8

Supplementary Figure 8. *Smad4* loss tunes signaling dependencies in PDAC with distinct KRAS status. (A) RNA-seq expression of iCAF and myCAF markers in PSCs flow-sorted from co-cultures with KvPC$^{Smad4\text{-WT}}$ or KvPC$^{Smad4\text{-KO}}$ PDAC organoids. Results show mean ± SEM. **, *P* < 0.01, Mann-Whitney test. **(B)** RNA-seq expression of *Tgfb1* in KvPC$^{Smad4\text{-WT}}$ or KvPC$^{Smad4\text{-KO}}$ organoids flow-sorted from monocultures or co-cultures with PSCs. Results show mean ± SEM. *, *P* < 0.05, Mann-Whitney test. **(C)** Table summarizing the experimental cohorts of orthotopic transplantation models of KPC$^{Smad4\text{-WT}}$ and KPC$^{Smad4\text{-KO}}$ organoids for the vehicle- and JAKi- studies in C57BL/6J mice. **(D)** Quantification of p-STAT3 stain in 2-week vehicle- or JAKi-treated KPC$^{Smad4\text{-WT}}$ and KPC$^{Smad4\text{-KO}}$ PDAC tumors taken on the same day of the last dose. Results show mean ± SEM from 2 experiments. **, *P* < 0.01; ***, *P* < 0.001, Mann-Whitney test. The results show that overall p-STAT3 levels are significantly downregulated upon JAK inhibition, validating targeting of the pathway in both KPC$^{Smad4\text{-WT}}$ and KPC$^{Smad4\text{-KO}}$ PDAC tumors. **(E)** Representative p-STAT3 IHC stains in vehicle- or JAKi- treated KPC$^{Smad4\text{-WT}}$ and KPC$^{Smad4\text{-KO}}$ PDAC tumors. Scale bars, 50 μm. **(F)** Analysis of tumor differentiation status in vehicle- and JAKi- treated KPC$^{Smad4\text{-WT}}$ (n=7 vehicle, n=9 JAKi) or KPC$^{Smad4\text{-KO}}$ (n=9 vehicle, n=9 JAKi) tumors. Results show data from 2 experiments. ***, *P* < 0.001, chi-square test. **(G)** Quantification of epithelial/stroma proportion (done by calculating the percentage of ECAD$^+$ area) in vehicle- and JAKi- treated KPC$^{Smad4\text{-WT}}$ (n=7 vehicle, n=9 JAKi) or KPC$^{Smad4\text{-KO}}$ (n=9 vehicle, n=9 JAKi) tumors. Results show data from 2 experiments. No statistical difference was found, as calculated by chi-square test. **(H)** Representative phospho-histone 3 (pH3) stains in 2-week vehicle- or JAKi- treated KPC$^{Smad4\text{-WT}}$ and KPC$^{Smad4\text{-KO}}$ PDAC tumors. Scale bars, 50 μm. **(I)** Quantification of pH3 stain vehicle- or JAKi- treated KPC$^{Smad4\text{-WT}}$ and KPC$^{Smad4\text{-KO}}$ PDAC tumors. Results show mean ± SEM. No statistical difference was found, as calculated by Mann-Whitney test. **(J)** Representative Ki67 stains in vehicle- or JAKi- treated KPC$^{Smad4\text{-WT}}$ and KPC$^{Smad4\text{-KO}}$ PDAC tumors. Scale bars, 50 μm. **(K)** Quantification of Ki67 stain in vehicle- or JAKi- treated KPC$^{Smad4\text{-WT}}$ and KPC$^{Smad4\text{-KO}}$ PDAC tumors. Results show mean ± SEM. No statistical difference was found, as calculated by Mann-Whitney test. **(L)** Flow cytometric analysis of live singlets (DAPI$^-$) from the total cell gate in vehicle- or JAKi- treated KPC$^{Smad4\text{-KO}}$ PDAC tumors. Results show mean ± SEM from 3 separate experiments. ***, *P* < 0.001, Mann-Whitney test. **(M)** Flow cytometric analysis of endothelial cells (CD31$^+$CD45$^-$), epithelial cells (CD45$^-$CD31$^-$EpCAM$^+$), immune cells (CD45$^+$) and CAFs (CD45$^-$CD31$^-$EpCAM$^-$PDPN$^+$) from live

singlets in vehicle- or JAKi- treated KPC$^{Smad4\text{-KO}}$ PDAC tumors. Results show mean ± SEM from 3 separate experiments. *, $P < 0.05$, Mann-Whitney test. **(N)** Flow cytometric analyses of myCAFs (Ly6C$^-$MHCII$^-$ CAFs), iCAFs (Ly6C$^+$MHCII$^-$ CAFs) and apCAFs (Ly6C$^-$MHCII$^+$ CAFs) from parental CAF gate in 2-week vehicle- or JAKi- treated KPC$^{Smad4\text{-KO}}$ PDAC tumors. Results show mean ± SEM from 3 separate experiments. ***, $P < 0.001$, Mann-Whitney test. **(O)** Flow cytometric analyses of myCAF/iCAF ratio from the parental CAF gate in vehicle- or JAKi- treated KPC$^{Smad4\text{-KO}}$ PDAC tumors. Results show mean ± SEM from 3 separate experiments. No statistical difference was found, as calculated by Mann-Whitney test. **(P)** Quantification of Masson's trichrome stain in vehicle- or JAKi- KPC$^{Smad4\text{-KO}}$ PDAC tumors. Results show mean ± SEM from 3 separate experiments. *, $P < 0.05$, Mann-Whitney test. **(Q)** Flow cytometric analysis of neutrophils (CD45$^+$CD11b$^+$Gr1$^+$) and macrophages (CD45$^+$Gr1$^-$CD11b$^+$F4/80$^+$) from live singlets in vehicle- or JAKi- treated KPC$^{Smad4\text{-KO}}$ PDAC tumors. Results show mean ± SEM from 3 separate experiments. *, $P < 0.05$, Mann-Whitney test. **(R)** Flow cytometric analysis of NK cells (CD45$^+$NK1.1$^+$), total T cells (CD45$^+$CD3$^+$TCRβ$^+$), CD4$^+$ T cells (CD45$^+$CD3$^+$TCRβ$^+$CD8$^-$CD4$^+$) and CD8$^+$ T cells (CD45$^+$CD3$^+$TCRβ$^+$CD4$^-$CD8$^+$) from live singlets in vehicle- or JAKi- treated KPC$^{Smad4\text{-KO}}$ PDAC tumors. Results show mean ± SEM from 3 separate experiments. *, $P < 0.05$; **, $P < 0.01$, Mann-Whitney test. **(S)** Flow cytometric analysis of TIGIT$^+$ NK cells, total T cells, CD4$^+$ T cells and CD8$^+$ T cells from the respective parental gate in vehicle- or JAKi- treated KPC$^{Smad4\text{-KO}}$ PDAC tumors. Results show mean ± SEM from 3 separate experiments. *, $P < 0.05$, Mann-Whitney test. **(T)** Representative FOXP3 stains in vehicle- or JAKi- treated KPC$^{Smad4\text{-KO}}$ PDAC tumors. Scale bars, 50 μm. **(U)** Quantification of Foxp3 stain in vehicle- or JAKi- treated KPC$^{Smad4\text{-KO}}$ PDAC tumors. Results show mean ± SEM. **, $P < 0.01$, Mann-Whitney test.

SUPPLEMENTARY TABLES

Supplementary Table S1. Clinical information and characteristics of *SMAD4* WT and *SMAD4*-deficient human PDAC tissues, related to Figure 1.

Supplementary Table S2. Clinical information and characteristics of *SMAD4* WT and *SMAD4*-deficient human PDAC organoids used for the generation of orthotopic transplantation models, related to Figure 1.

Supplementary Table S3. Single-cell RNA-sequencing of murine KPC PDAC tumors – differential expression analysis and GSEA, related to Figures 2, 3, 4 and 5.

Supplementary Table S4. Ligand activity and ligand-target results from NicheNet analyses showing the top ligands that regulate a group of targeted genes. Data are from single-cell RNA-sequencing of murine KPC PDAC tumors, related to Figure 3.

Supplementary Table S5. RNA-sequencing of PSCs flow-sorted from KPC PDAC organoid co-cultures – differential expression analysis, normalized expected counts and GSEA, related to Figure 4.

Supplementary Table S6. RNA-sequencing of *Smad4* KO and *Smad4* WT KPC PDAC organoids flow-sorted from co-cultures with PSCs – differential expression analysis, normalized expected counts and GSEA, related to Figure 5.

Supplementary Table S7. Ligand activity results from NicheNet analyses showing the top ligands that regulate a group of targeted genes. Data are from RNA-sequencing of *Smad4* KO and *Smad4* WT KPC PDAC organoids flow-sorted from monocultures or co-cultures with PSCs, and of PSCs, related to Figure 5.

Supplementary Table S8. RNA-sequencing of *Smad4* KO and *Smad4* WT KPC PDAC tumors – GSEA, related to Figure 5.

Supplementary Table S9. Transcription factor analysis of *Smad4* KO and *Smad4* WT KPC PDAC organoids flow-sorted from co-cultures with PSCs, related to Figure 5.

Supplementary Table S10. Single-cell RNA-sequencing of murine KvPC PDAC tumors – GSEA, related to Figures 6, 7 and 8.

Supplementary Table S11. Ligand activity and ligand-targets results from NicheNet analyses showing the top ligands that regulate a group of targeted genes. Data are from single-cell RNA-sequencing of murine KvPC PDAC tumors, related to Figures 6 and 7.

Supplementary Table S12. RNA-sequencing of PSCs flow-sorted from KvPC PDAC organoid co-cultures – differential expression analysis, normalized expected counts and GSEA, related to Figure 8.

Supplementary Table S13. RNA-sequencing of *Smad4* KO and *Smad4* WT KvPC PDAC organoids flow-sorted from co-cultures with PSCs – differential expression analysis, normalized expected counts and GSEA, related to Figure 8.
